# Alterations of Gut Microbiome and Metabolite Profiling in Mice Infected by *Schistosoma japonicum*

**DOI:** 10.3389/fimmu.2020.569727

**Published:** 2020-10-08

**Authors:** Yue Hu, Jiansong Chen, Yiyue Xu, Hongli Zhou, Ping Huang, Yubin Ma, Minzhao Gao, Shaoyun Cheng, Haiyun Zhou, Zhiyue Lv

**Affiliations:** ^1^Key Laboratory of Tropical Disease Control (Sun Yat-sen University), Ministry of Education, Guangzhou, China; ^2^Key Laboratory of Tropical Translational Medicine of Ministry of Education, Hainan Medical University, Haikou, China; ^3^Joint Program of Pathobiology, Fifth Affiliated Hospital, Zhongshan School of Medicine, Sun Yat-sen University, Guangzhou, China; ^4^Instrumental Analysis and Research Center, Sun Yat-sen University, Guangzhou, China; ^5^Department of Gastroenterology, The Fifth Affiliated Hospital of Sun Yat-sen University, Zhuhai, China; ^6^Provincial Engineering Technology Research Center for Biological Vector Control, Zhongshan School of Medicine, Sun Yat-sen University, Guangzhou, China

**Keywords:** *Schistosoma japonicum*, gut microbiome, metagenomics, metabolomics, *16S* rRNA, UPLC-MS 3

## Abstract

*Schistosoma japonicum* (*S. japonicum*) is one of the etiological agents of schistosomiasis, a widespread zoonotic parasitic disease. However, the mechanism of the balanced co-existence between the host immune system and *S. japonicum* as well as their complex interaction remains unclear. In this study, *16S* rRNA gene sequencing, combined with metagenomic sequencing approach as well as ultraperformance liquid chromatography–mass spectrometry metabolic profiling, was applied to demonstrate changes in the gut microbiome community structure during schistosomiasis progression, the functional interactions between the gut bacteria and *S. japonicum* infection in BALB/c mice, and the dynamic metabolite changes of the host. The results showed that both gut microbiome and the metabolites were significantly altered at different time points after the infection. Decrease in richness and diversity as well as differed composition of the gut microbiota was observed in the infected status when compared with the uninfected status. At the phylum level, the gut microbial communities in all samples were dominated by Firmicutes, Bacteroidetes, Proteobacteria, and Deferribacteres, while at the genus level, *Lactobacillus, Lachnospiraceae NK4A136 group, Bacteroides, Staphylococcus*, and *Alloprevotella* were the most abundant. After exposure, *Roseburia*, and *Ruminococcaceae UCG-014* decreased, while *Staphylococcus, Alistipes*, and *Parabacteroides* increased, which could raise the risk of infections. Furthermore, LEfSe demonstrated several bacterial taxa that could discriminate between each time point of *S. japonicum* infection. Besides that, metagenomic analysis illuminated that the AMP-activated protein kinase (AMPK) signaling pathway and the chemokine signaling pathway were significantly perturbed after the infection. Phosphatidylcholine and colfosceril palmitate in serum as well as xanthurenic acid, naphthalenesulfonic acid, and pimelylcarnitine in urine might be metabolic biomarkers due to their promising diagnostic potential at the early stage of the infection. Alterations of glycerophospholipid and purine metabolism were also discovered in the infection. The present study might provide further understanding of the mechanisms during schistosome infection in aspects of gut microbiome and metabolites, and facilitate the discovery of new targets for early diagnosis and prognostic purposes. Further validations of potential biomarkers in human populations are necessary, and the exploration of interactions among *S. japonicum*, gut microbiome, and metabolites is to be deepened in the future.

## Introduction

Schistosomiasis is a zoonotic parasitic disease mainly caused by the infection of *Schistosoma japonicum* (*S. japonicum*), *Schistosoma mansoni (S. mansoni)*, and *Schistosoma haematobium (S. haematobium)*, which seriously damages human and animal health and hinders socio-economic development. This neglected tropical disease affects ~200 million people, and ~779 million are at risk of infection worldwide (1). *S. japonicum* is distributed principally in East Asia, especially in China, the Philippines, and Indonesia, with more than 1 million people infected and ~46 million people at risk (2). In China, *S. japonicum* is endemic in mainly 12 provinces along the middle and the lower reaches of the Yangtze River and regions south of it. The life cycle of *S. japonicum* is complex and consists of asexual generation in the intermediate host and sexual generation in the definitive host, including the seven developmental stages of egg, miracidium, mother sporocyst, daughter sporocyst, cercaria, juvenile schistosomulum, and adult worm (3). Different stages of *S. japonicum* cause various damages to the host, and complex immune pathological reactions lead to diverse clinical symptoms. Larval worms induce Th1 responses with elevated levels of the inflammatory cytokines IFN-γ, IL-12, and TNF-α in the early phase of schistosomiasis and cause diarrhea, fatigue, and anemia, while adult worms become mature and lay eggs; parasite eggs that deposit in the liver and colon of infected hosts elicit Th2 responses and then upregulate the serum cytokine levels of IL-4, IL-5, IL-13, and TGF-β, leading to portal vein hypertension syndrome, ascites, and liver fibrosis (2, 4, 5). During *S. japonicum* infection, egg deposits in the tissues are a determining factor that shifts the Th1 response to the Th2 response (5). To protect against eggs, delayed-type hypersensitivity reactions of the host are triggered but lead to the formation of circumoval granuloma in livers and colons, followed by fibrosis, which is the main cause of death (5). Immunological downregulation occurs to both, protecting the host from inflammatory damage and preventing the parasites from being eliminated during late chronic infection (4). However, the mechanism of this balanced co-existence between the host's immune system and *S. japonicum* as well as their complex interaction remains unclear.

At present, schistosomiasis is diagnosed by a clinical history of contact with fresh water from endemic foci, followed by both direct methods and indirect tests in the clinical laboratory. The former includes using the Kato–Katz technique to examine the feces under light microscopy for detection of eggs, while the latter includes using immunological approaches, such as detecting soluble antigens secreted from eggs *via* the antigen–antibody reaction (2, 6, 7). Imaging methods, for instance, ultrasonography (US), CT scan and MR, scan, are established to inspect the presence of periportal fibrosis (7). Nevertheless, these diagnostic methods are not sensitive or specific enough and are not suitable for early diagnosis; therefore, a well-suited approach with high sensitivity and specificity is urgently required to detect acute stage infection.

Metabolomics is a quantitative measurement of multi-parametric metabolic responses of multi-cellular systems and aims to identify and quantify numerous small molecules (<1,200 Da) present in various biological samples or specific physiological states; metabolomics can provide a comprehensive systems-level study of the relationships between host genetic and environmental factors with high-density data and multivariate mathematical modeling (8–10). Nuclear magnetic resonance (NMR) spectroscopy, liquid chromatography–mass spectrometry (LC–MS), and gas chromatography–mass spectrometry are the most widely used analytical techniques for metabolomic analyses. Due to the main advantages of much better sensitivity and resolution, more coverage of metabolites, and high-throughput capacity (11), MS-based techniques have been implemented more frequently than NMR. Metabolomics has already been widely applied in parasitological studies, with comprehensive characterizations of the host metabolic responses to infections by several parasites, such as *Plasmodium falciparum* (12–14), *Trypanosoma brucei* (15–17), *Toxoplasma gondii* (18–20), and *S. japonicum* (2, 6, 21). Hence, metabolomics is a suitable diagnostic tool to provide novel insights into the mechanisms underlying the progression of schistosomiasis, clearly revealing the resistance mechanism between the host and *S. japonicum* and thus leading to the discovery of potential metabolic biomarkers that are useful for early diagnosis.

The gut microbiota, consisting of diverse microbial communities, has a profound impact on influencing host physiology by the composition, density, and activities of colonizing microorganisms as well as on animal evolution through the interplay between host and microbial communities (22). Once the balance in proportions among core bacterial communities breaks down, dysbiosis occurs, which may alter host interactions and lead to numerous diseases, such as inflammatory gut disorders, diabetes, and obesity (23). *S. japonicum* adult worms live in the mesenteric veins of the host and can cause intestinal schistosomiasis with symptoms of mucosal granulomatous inflammation, superficial bleeding, and pseudopolyposis (24). However, the interaction between the host, *S. japonicum*, and the microbiota and the potential effects of helminths on shaping the host–microbiota composition and structure are unclear; therefore, further investigation is warranted.

Previous studies have shown that infection with *S. japonicum* modified both bacterial richness and bacterial community composition (25), reduced the levels of tricarboxylic acid cycle intermediates, increased the levels of amino acids, choline, and urinary 3-ureidopropionate, and perturbed lipid metabolism, glycolysis stimulation, tricarboxylic acid cycle, and a series of microbial-related metabolites (2, 6). Nevertheless, the alterations of microbiome and metabolome in the time course of infection progression have not been described yet; therefore, the aim of the current study is to investigate the dynamic alteration of gut microbiome community structure and the metabolite profile of the hosts infected with *S. japonicum*, and the correlations between host metabolism and gut microbiome after the infection by omics-based and systems-level approaches involving metabolic profiling with ultraperformance liquid chromatography–mass spectrometry (UPLC–MS), *16S* rRNA gene sequencing, and shotgun metagenomics sequencing. To our knowledge, employing metagenomic sequencing and untargeted metabolic profiling to investigate the effects of infection with *S. japonicum* in BALB/c mice would allow a better understanding of the mechanisms during schistosomiasis development and potentially reveal new targets for early diagnosis and prognosis.

## Materials and Methods

### Ethics Statement

All animal experiments were conducted strictly in accordance with the guidelines of the National Institutes of Health on animal care and the ethical guidelines. The protocol was approved by the Animal Care and Use Committee of Sun Yat-sen University [permit no. SYXK (Guangdong) 2017-0081]. All efforts were made to minimize the suffering of the animals.

### Mice and Cercariae

Seventy specific-pathogen-free 6- to 8-week-old female BALB/c mice (18 ± 2 g body weight) were purchased from the Animal Experiment Center at Sun Yat-sen University (Guangzhou, China) and were housed in plastic cages with free access to autoclaved chow and water under controlled temperature and humidity and a 12-h light/12-h dark cycle. The animals were randomly divided into seven groups, with 10 mice in each group. After the mice acclimated to the new environment, 60 of them were infected with 30 ± 2 *S. japonicum* cercariae per individual *via* the shaved abdominal skin. The cercariae were obtained from infected *Oncomelania*, which were provided by the National Institute of Parasitic Diseases, Chinese Center for Disease Control and Prevention (Shanghai, China), that were placed in dechlorinated water and exposed to artificial light for more than 2 h. The other mice were left uninfected and served as controls.

### Sample Collection

The mice in each group were sacrificed by chloral hydrate asphyxiation and cervical dislocation either before infection or at 3, 7, 14, 21, 28, and 42 days post-infection (dpi). Blood samples were drawn from orbital veins and centrifuged at 3,000 ×*g* for 10 min to collect the serum after clotting. The serum was stored at−80°C until further analysis. Urine and feces samples were collected the day before the mice were sacrificed by placing them individually in metabolic cages, which can separate feces from urine using different small plastic tubes embedded at the bottom of the cages. Dry ice was placed around the collection tubes to prevent oxidation or degradation of metabolites. At least 0.5 ml of urine and 1 g of feces were obtained; then, the samples were transferred into Eppendorf tubes and stored in a freezer at−80°C for further testing.

### Genomic DNA Extraction and *16S* rRNA Gene Sequencing

Nucleic acid extraction of thirty fecal samples of the 0, 7, 14, 21, 28, and 42 dpi groups (five samples per group) was carried out using a QIAamp Fast DNA Stool Mini kit (cat. no. 51604, QIAGEN, Hilden, Germany), following the manufacturer's instructions. The concentration of DNA was measured on a NanoDrop (Thermo Scientific, Waltham, MA, USA), and 1% agarose gel electrophoresis was used to assess the integrity and the purity of DNA. After that, extracted DNA was diluted to a concentration of 1 ng/μl and used as a template to amplify the V3–V4 regions of the *16S* rRNA gene, utilizing the primers 343F (5′-TACGGRAGGCAGCAG-3′) and 798R (5′-AGGGTATCTAATCCT-3′) with barcodes in combination with HiFi Hot Start Ready Mix (cat. no. KK2501, Kapa Biosystems, Boston, MA, USA). Polymerase chain reaction (PCR) amplicon product quality was demonstrated through agarose gel electrophoresis, and PCR amplicons were purified with Agencourt AMPure XP beads (cat. no. A63881, Beckman Coulter, Brea, CA, USA), followed by another round of PCR amplification. The final amplicons were quantified with a Qubit® dsDNA HS Assay Kit (cat. no. Q32854, Life Technologies, Waltham, MA, USA) after a subsequent clean-up, as described above. Afterwards, the purified amplicons from each sample were pooled in equal amounts for subsequent *16S* rRNA sequencing on the Illumina MiSeq platform.

### Metagenome Sequencing

Two microliters of DNA (10 ng/μl) from nine fecal samples of the 0, 21, and 42 dpi groups (three samples per group) was fragmented to ~300–500 bp with a Covaris S220 (Covaris, Woburn, MA, USA) individually. Subsequently, library construction was performed using a TruSeq Nano DNA LT Sample Preparation Kit (cat. no. FC-121-4001, Illumina, San Diego, CA, USA) according to the manufacturer's instructions, and then a TruSeq PE Cluster Kit v3-cBot-HS (cat. no. PE-401-3001, Illumina, San Diego, CA, USA) was used for bridge PCR. The resulting DNA was then pooled and quantified by Kapa Library Quantification Kits (cat. no. KK4824, Kapa Biosystems, Boston, MA, USA) and sequenced using the Illumina HiSeq platform with a TruSeq SBS Kit v3-HS (cat. no. FC-401-3001, Illumina, San Diego, CA, USA).

### *16S* rRNA Gene Analysis

Paired-end reads were reprocessed using Trimmomatic software (26) to detect and cut off ambiguous bases from the N terminal. Low-quality sequences with an average quality score lower than 20 were removed by the sliding window trimming approach. After trimming, the paired-end reads were assembled using Fast Ligation-based Automatable Solid-phase High-throughput software (version 1.2.11) (27). Only sequences with 10 base pairs (bp) of minimal overlapping, 200 bp of maximum overlapping, and 20% of maximum mismatch rate were assembled according to their overlap sequence. Reads with ambiguous, homologous sequences and a total length of <200 bp were abandoned, while reads with 75% of bases above Q20 were retained. Next, the sequences were checked by Quantitative Insights Into Microbial Ecology (QIIME) software (version 1.8.0) (28) for the following criteria: the maximum length of a homopolymer run was six, the maximum number of mismatches in the primer was two, the maximum number of errors in the barcode was zero, and reads with chimeras were detected and removed. After that, clean reads were obtained, followed by subjecting to primer sequence removal and clustering to generate operational taxonomic units (OTUs) using UPARSE software (version 6.1.351) with 97% sequence similarity cutoff (equal to bacterial species level) (29). All representative reads were chosen from each OTU by selecting the most abundant sequence with the QIIME package. High-quality representative sequences were annotated and blasted against the Silva database (version 123) on the basis of the Ribosomal Database Project classifier (the confidence threshold was set as 70%) (30).

Alpha diversity (within-sample diversity) was estimated for each group using the number of observed species, Chao1 richness estimator, Shannon–Wiener index, and phylogenetic diversity index, while beta diversity (between-sample diversity) was monitored with two-dimensional and three-dimensional principal coordinate analysis (PCoA) plots on the basis of weighted UniFrac distance metrics. Bar plots were generated to visualize the relative abundances and alterations over time in bacterial communities for fecal samples from each group. To distinguish significant differences in microbial communities at different taxonomic levels between healthy mice and *S. japonicum-*infected mice, one-way analysis of variance (ANOVA) was performed; statistical significance level was set at *p* < 0.05. Linear discriminant analysis (LDA) coupled with effect size (LEfSe) measurement^1^ was implemented to illuminate microbial taxa that were differentially represented between the groups, in order to discover the potential markers at different time points. Pearson correlation coefficients between the top 30 dominant gut bacteria at the genus level were calculated, and bacterial genera with high correlations and *p* < 0.05 were used to build an associated network with Cytoscape software (version 3.6.1) to find correlations between gut microbiota changes and explore the ecological significance related to each other. Unless otherwise stated, statistical analyses and plots were carried out using R software (version 3.5.1).

### Metagenomic Analysis

Next-generation sequencing quality control (QC) Toolkit (version 2.3.2) (31) was used to discard raw metagenomic reads with 70% of bases below Q20 and remove reads from the 3′ end until reaching the first nucleotide with a minimum quality score cutoff of 20 and if either read was shorter than 70 bp or contained “N” bases or ambiguous bases. Afterwards, sequences of *Mus musculus*^2^ were filtered out by Burrows–Wheeler Alignment (version 0.7.9a) (32). After the scaffold sequences from all samples were assembled with Short Oligonucleotide Analysis Package *Denovo*^3^ (version 4.5.4) (33), open reading frames (ORFs) were predicted and translated into amino acid sequences by prodigal^4^ (version 2.6.3) (34). Cluster Database at High Identity with Tolerance^5^ (version 4.5.4) was implemented to build non-redundant gene sets for all predicted genes and to cluster ORFs with more than 95% identity and more than 90% coverage. The gene with the longest full length from each cluster was selected as the representative read of each gene set. For further analysis, annotations were performed with the gene set representative reads by using Blastp^6^ (Blast version 2.2.28+) alignment (E-value < 0.00001) between ORFs and the protein databases of the Kyoto Encyclopedia of Genes and Genomes^7^.

### Metabolic Profiling

#### Sample Processing for Metabolomics

Each 10-μl serum sample was thawed on ice and diluted 1:10 in methanol. After that, the mixture was vortexed briefly and incubated overnight at 4°C to precipitate proteins thoroughly, followed by centrifugation at 10,000 ×*g* for 10 min, and ~80-μl aliquots of the supernatant were then transferred to a vial for analysis. The urine samples were prepared by diluting 50 μl of urine with 450 μl of Milli-Q water and centrifuging for 10 min at 10,000 ×*g* at 4°C to remove particulates. A 200-μl volume of supernatant was collected and transferred into a glass vial afterwards. Liver and colon samples were processed following a modified version of the method of Elizabeth et al. (35). Briefly, frozen tissue (50 ± 0.5 mg) was added to 1.5 ml of prechilled methanol/water (1:1, v:v) solvent, which was subsequently completely homogenized in an ice bath. The suspension was centrifuged at 16,000 ×*g* for 10 min, and the resulting supernatant was transferred into an Eppendorf tube and then processed through vacuum freeze-drying to obtain an aqueous extract. The dried residue was redissolved in 120 μl of methanol/water (1:1, v:v) and centrifuged at 13,000 ×*g* for 10 min to remove particulates. Thereafter, the clear supernatant was transferred to a sampling vial. For each type of specimen, QC samples were prepared by mixing an equal volume of all individuals into the vial to ensure the repeatability and the stability of the analysis.

#### Ultraperformance Liquid Chromatography Conditions

An ACQUITY UPLC-I Class System (Waters Ltd., Milford, MA, USA) was used to perform chromatographic separations. Each sample was injected onto an ACQUITY UPLC C18 BEH column (2.1 × 100 mm, 1.7 μm; Waters, Milford, MA, USA) in a random order at 38°C, while QC samples were detected every 10 specimens throughout the injection.

For the serum samples, mobile phase A was water mixed with 0.1% formic acid, while mobile phase B was 70% isopropanol and 30% acetonitrile containing 0.1% formic acid. The serum samples were eluted under gradient conditions at a flow rate of 400 μl/min with 1% B, which was held for 1 min and then was ramped up from 1 to 40% B for 2 min, from 40 to 75% B for 5 min, from 75 to 85% B for 4 min, and from 85 to 99% B for 6 min, held at 99% B over 4 min, and then returned to 1% B for 3 min. The volume of the sample injected onto the column was 1.000 μl.

For the urine samples, the gradient solvent system included water (A) and acetonitrile (B), each containing 0.1% formic acid. The injection volume of the urine samples was set to 0.300 μl; the separation gradient was held at 3% B for 1.2 min, ramped up from 3 to 45% B over 8.8 min and from 45 to 98% B for 4 min, held at 98% B over 2 min, and then returned to 3% B for 3 min, with a flow rate of 400 μl/min.

For the liver supernatant, the injection volume was 0.400 μl; the mobile phase was held at 2% B for 2 min, increased from 2 to 45% B over the next 8 min, followed by 45 to 98% B over 3 min, held at 98% B for 2 min, and decreased to 2% B, which was held for 2 min, at a flow rate of 400 μl/min. However, the injection volume of the colon supernatant was set to 1.500 μl; the separation gradient was held at 25% B for 0.5 min, ramped from 25 to 50% B over 4.5 min, from 50 to 65% B for 7 min, and from 65 to 95% B for 4 min, held 95% B for 2 min, and then returned to 25% B over 3 min, with a flow rate of 400 μl/min. The gradient solvent systems used for the liver and the colon supernatants were the same as that used for the urine samples.

#### Quadrupole-Time-of-Flight Mass Spectrometry Conditions

Mass spectrometry data were collected by a SYNAPT G2-Si High-Definition Mass Spectrometer with an electrospray ionization (ESI) source (Waters Ltd., Milford, MA, USA) in both positive and negative ion modes for the serum and the urine samples, whereas only negative ion mode was used for the liver and colon aqueous extracts due to the limited valuable compounds detected in the positive ion mode. Nitrogen gas was set as desolvation and cone gas. The capillary voltage was set at 2.5 kV, nebulizer gas at 6 bar, cone voltage at 35 kV, cone gas flow at 30 L/h, source temperature at 110°C, desolvation gas temperature at 350°C, and desolvation gas flow at 700 L/h. The eluted compounds were scanned from mass/charge (*m/z*) 50 to *m/z* 1,200 at a rate of 0.3 s per scan for both MS mode and MS^E^ mode. The collision energy was set from 20 to 50 eV for MS^E^ mode. To ensure mass accuracy and reproducibility, leucine enkephalin was used to correct data (*m/z* 556.2720 in positive mode and *m/z* 554.2615 in negative mode) at a concentration of 1 ng/μl and a flow rate of 5 μl/min continuously.

#### Data Analysis

The raw data were acquired by Masslynx (version 4.1, Waters, Manchester, UK) and imported into Progenesis QI (version 2.1, Nonlinear Dynamics, Waters, Manchester, UK) for data preprocessing, including peak alignment, picking, and normalization as well as compound identification. Normalized and scaled datasets were imported into SIMCA-P (version 13.0, Umetrics, Umea, Sweden) and MetaboAnalyst 4.0^8^ (36, 37) to carry out statistical analyses. The statistical significance between experimental groups was determined by one-way ANOVA, with *p* < 0.05. Principal component analysis (PCA) and partial least squares-discriminatory analysis (PLS-DA) were used to visualize natural separation and trends among the groups by score plots, while orthogonal partial least squares-discriminatory analysis (OPLS-DA) was conducted to find the maximum separation between healthy mice and *S. japonicum*-infected mice. Advanced statistical and visualization tools, such as variable importance in projection (VIP) and S-plots, were performed to reveal underlying trends in data. The discriminated metabolites were selected based on significant changes, including VIP scores that were taken from comparisons in OPLS-DA models >2, *p* < 0.05, and QC samples' coefficient of variation (CV) <30 and were putatively identified by searching databases such as the Human Metabolome Database^9^ (38) and METLIN^10^ (39) with accurate mass spectral data and MS/MS spectra. Furthermore, receiver operating characteristic (ROC) curve analysis was carried out to evaluate the early diagnostic capability of identified potential biomarkers. Metabolite set enrichment analysis (MSEA) and pathway analysis were performed by MetaboAnalyst 4.0 to investigate the most significant metabolic pathways involved in *S. japonicum* infection. Finally, correlations between the dominant gut bacteria changes and shifted metabolites were calculated in R software (version 3.5.1) by a hierarchical clustering algorithm to determine the relationships between them.

## Results

### Gut Microbiome Community Structure Changes in Mice Infected by *S. japonicum*

High-throughput sequencing of the *16S* rRNA gene was implemented to illustrate the alterations in the gut bacterial compositions of BALB/c mice associated with *S. japonicum* infection. A total of 1,210,516 valid reads were retained from 30 fecal samples, with an average of 40,350 sequences per sample, for further processing after filtering, which generated 19,868 OTUs at 97% similarity level (Supplementary Table 1) and 56 OTUs shared among all samples (Supplementary Figure 1). Most of the shared OTUs are members of the families Lachnospiraceae, Ruminococcaceae, and Bacteroidales S24-7 group. The four different alpha diversity estimators mentioned above increased over time among all groups but to a lesser extent in the infected mice than in the uninfected mice, whereas the least diversity presented at 21 dpi, indicating that the infection of *S. japonicum* reduced the alpha diversity of host gut bacteria, especially at 21 dpi (Supplementary Figure 2). However, the differences were not statistically significant (*p* > 0.05). A three-dimensional PCoA plot based on weighted UniFrac distances (Supplementary Figure 3) showed the variations of the gut bacterial communities of BALB/c mice, with 49.84, 18.29, and 7.12% variation explained by principal component (PC) 1, PC2, and PC3, respectively. The difference was observed between the 0-dpi group and the 42-dpi group, which demonstrated that the effect of *S. japonicum* infection in the late stage on the gut microbiome composition of the host was relatively stronger.

### Perturbed Gut Bacteria in Mice With *S. japonicum* Infection

**Figure 1:**
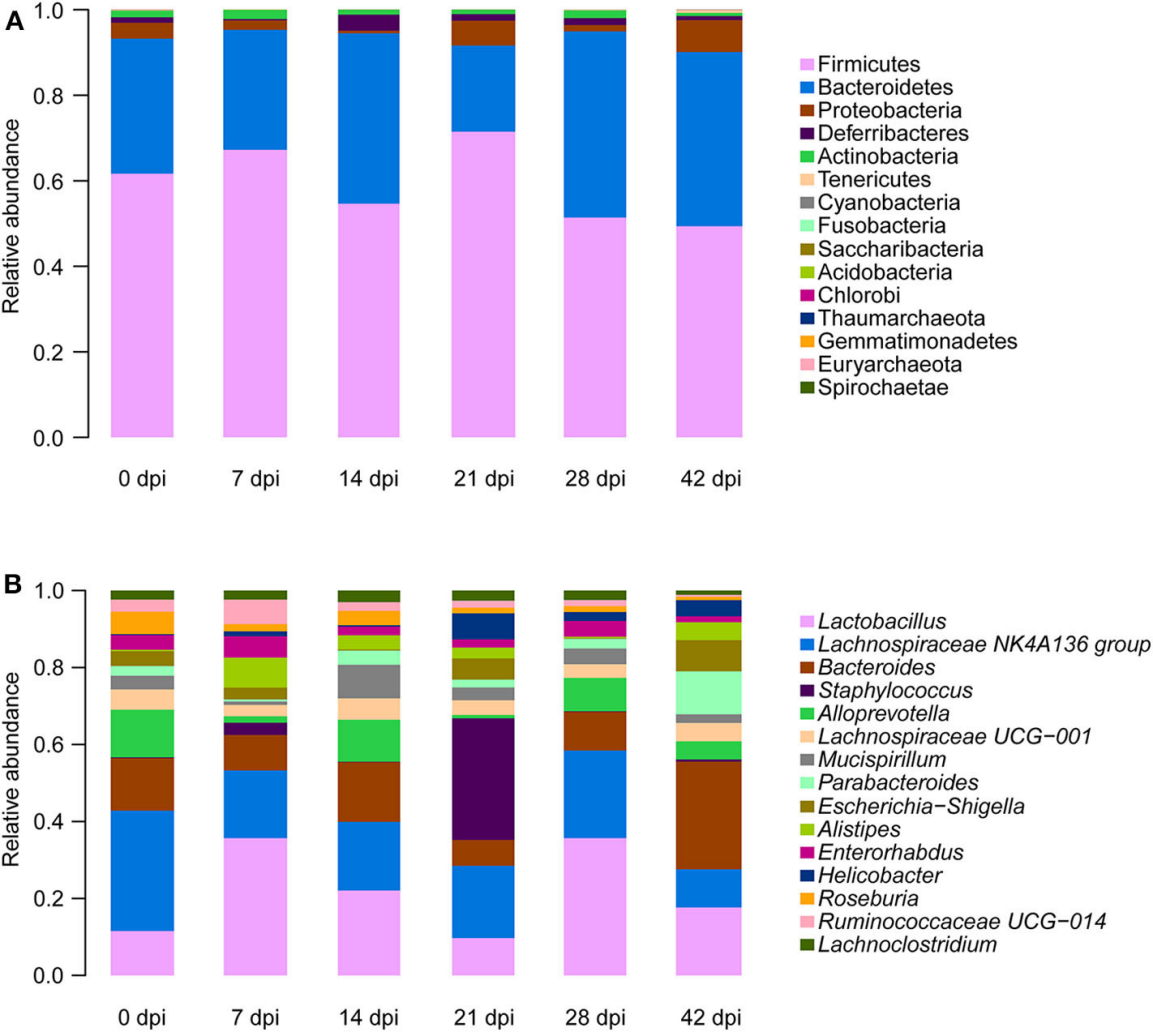
Relative abundances of the top 15 most important gut microbiota constituents at the phylum level **(A)** and genus level **(B)** across different time points as assessed by *16S* rRNA sequencing. Each column represents the composition of the microbial taxa in one group.

**Figure 2:**
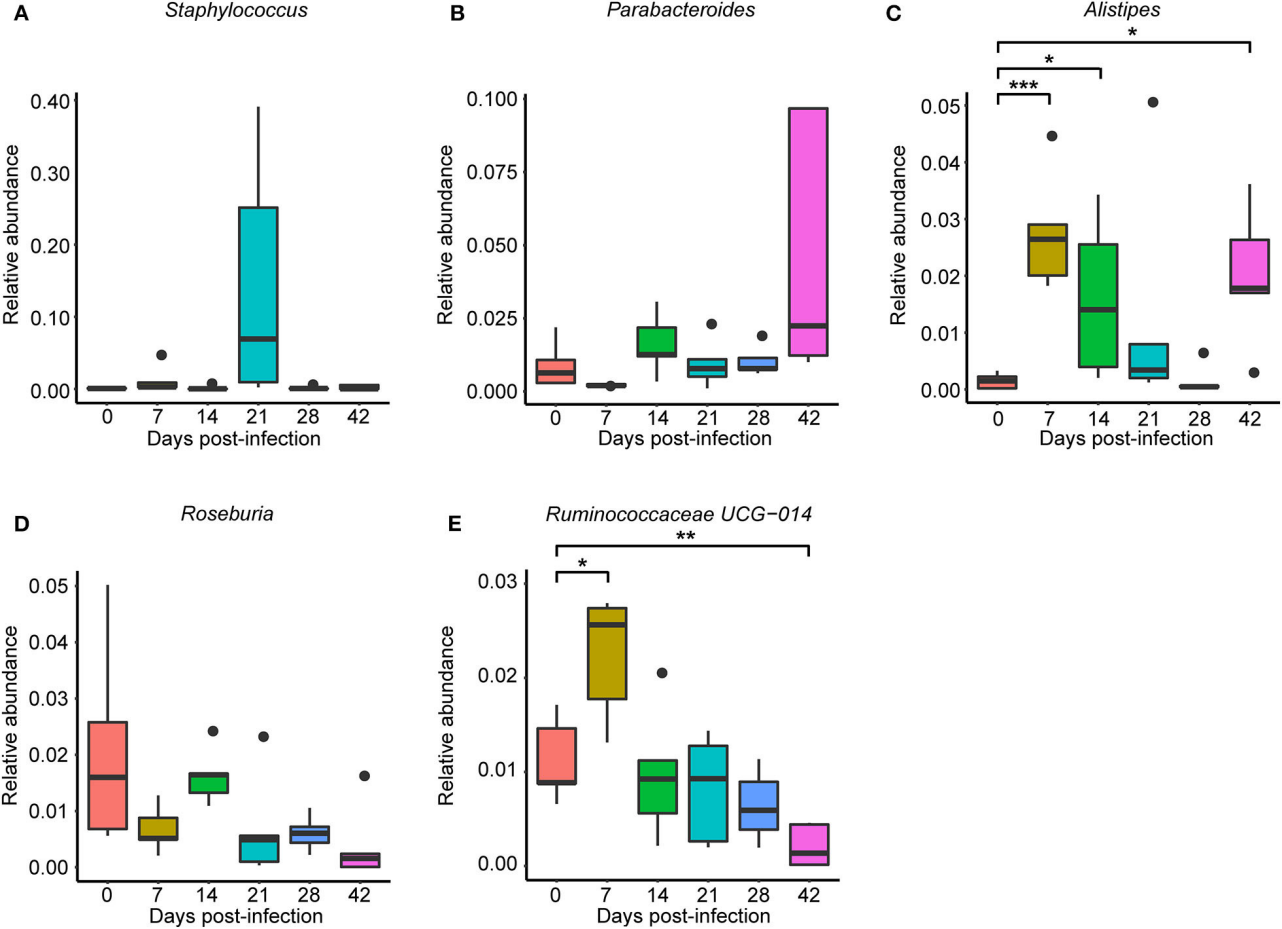
Relative abundances of significantly altered top 15 most important gut genera associated with *Schistosoma japonicum* infection. Each column represents one group. **(A)** *Staphylococcus*. **(B)** *Parabacteroides*. **(C)** *Alistipes*. **(D)** *Roseburia*. **(E)** *Ruminococcaceae UCG-014*. The top and the bottom whiskers indicate the maximum and the minimum values, respectively, and the hyphen represents the median value (*p < 0.05, **p < 0.01, ***p < 0.001).

To observe *S. japonicum* infection effects on the gut microbiome of BALB/c mice, bar plots were generated according to the relative abundance of the 15 most abundant gut bacterial phyla (Figure 1A) and genera in different groups (Figure 1B). Subsequently, one-way ANOVA was applied to identify significant alterations in the composition of the host gut microbiota at the phylum and the genus levels during infection. It is obvious that Firmicutes and Bacteroidetes were the most abundant gut bacterial phyla, with total average relative abundances over 90% in all groups, and the abundances of Proteobacteria and Deferribacteres were altered according to the time of infection but with no statistical significance (*p* > 0.05, Supplementary Table 2). At the genus level, *Lactobacillus, Lachnospiraceae NK4A136* group, *Bacteroides, Staphylococcus*, and *Alloprevotella* were the most prevalent gut microbiome in BALB/c mice, but only alterations of the relative abundances of *Staphylococcus, Parabacteroides, Alistipes, Roseburia*, and *Ruminococcaceae UCG-014* showed statistical significance in the top 15 most important gut genera (*p* < 0.05, Supplementary Table 3). Notably, *Staphylococcus*, with a relative abundance of 14.46% in the 21-dpi group, was almost undetectable in other groups (Figure 2A). Furthermore, the average relative abundance of *Parabacteroides* was decreased before 7 dpi, but it was increased after that (Figure 2B); *Alistipes* was significantly more abundant in the infected groups than in the uninfected group (Figure 2C). In contrast, *Roseburia* and *Ruminococcaceae UCG-014* (Figures 2D,E) were reduced after infection with *S. japonicum*, while the average relative abundance of the latter increased approximately twofold at 7 dpi.

**Figure 3:**
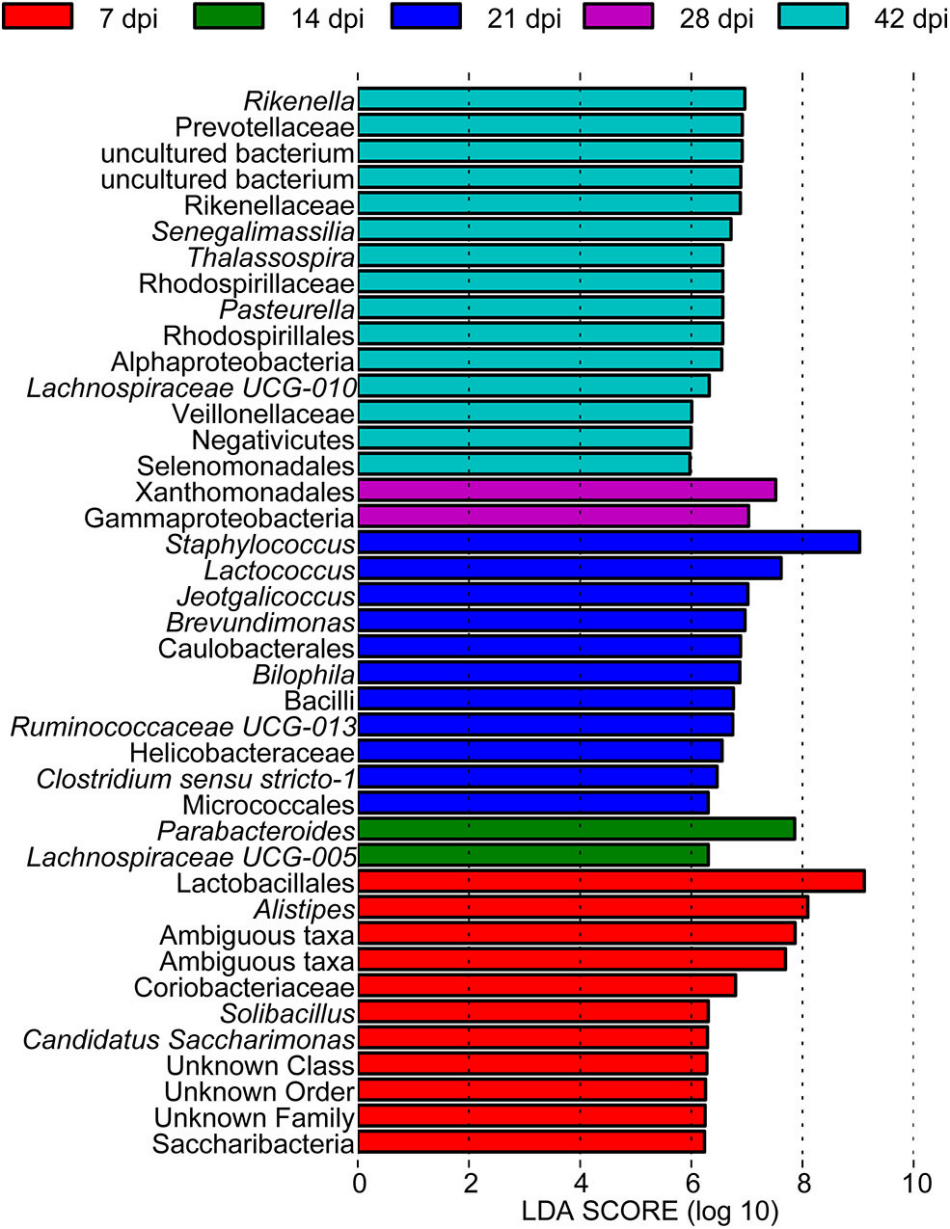
A histogram with linear discriminant analysis scores showing differentially abundant gut bacteria during the course of *Schistosoma japonicum* infection. Taxa highlighted in different colors indicate over-representation in the corresponding groups.

**Figure 4:**
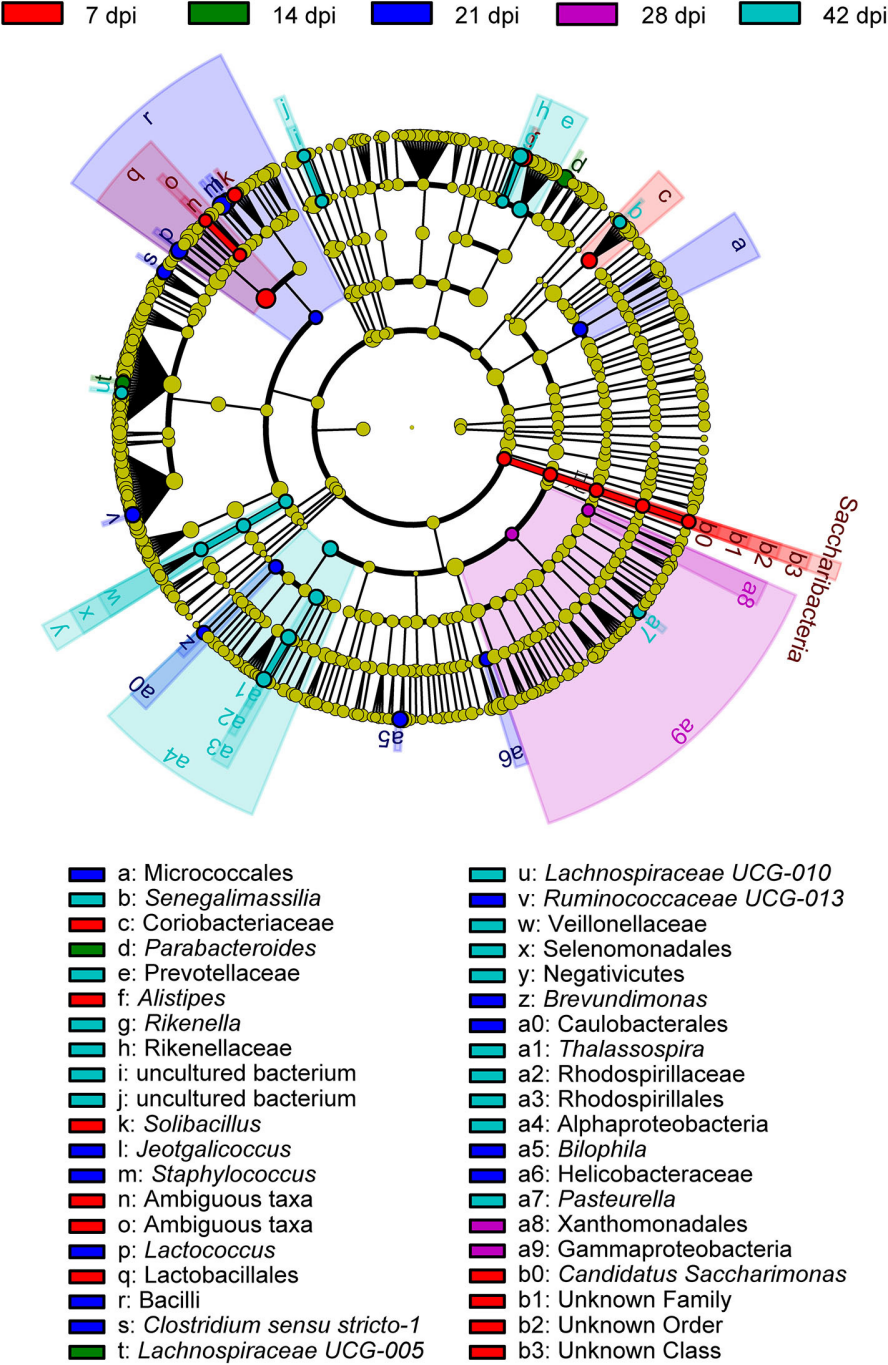
A cladogram showing the discriminated taxa in different groups. Regions with different colors represent different groups. Differently colored nodes in the branches represent the microbial groups that play an important role in the corresponding groups, whereas yellow nodes indicate bacterial groups that are insignificant in all groups.

To determine the taxa ranging from the phylum to the genus level that discriminated between each time point of *S. japonicum* infection, LEfSe, with an adjusted *p* < 0.05 and LDA score threshold >6.0, was performed. Bacteria from the phyla Firmicutes and Saccharibacteria, in addition to *Alistipes* and other taxa, were significantly associated with the 7-dpi group, while only *Parabacteroides* and *Lachnospiraceae UCG-005* were significantly related to the 14-dpi group. Some members of the phyla Firmicutes and Proteobacteria were significantly enriched in the 21-dpi group, but the most significant was *Staphylococcus*. The Gammaproteobacteria class significantly distinguished the 28-dpi group from the other groups. Over-abundances of other members from the phyla Firmicutes, Bacteroidetes, and Proteobacteria were significantly linked to the 42-dpi group. However, no discriminative gut flora was found in the 0-dpi group. It is worth noting that *Lachnospiraceae UCG-005* was related to the 14-dpi group, whereas *Lachnospiraceae UCG-010* was associated with the 42-dpi group (Figure 3). The cladogram in Figure 4 showed the most relevant clades among each group, which was in accordance with the above mentioned results.

**Figure 5:**
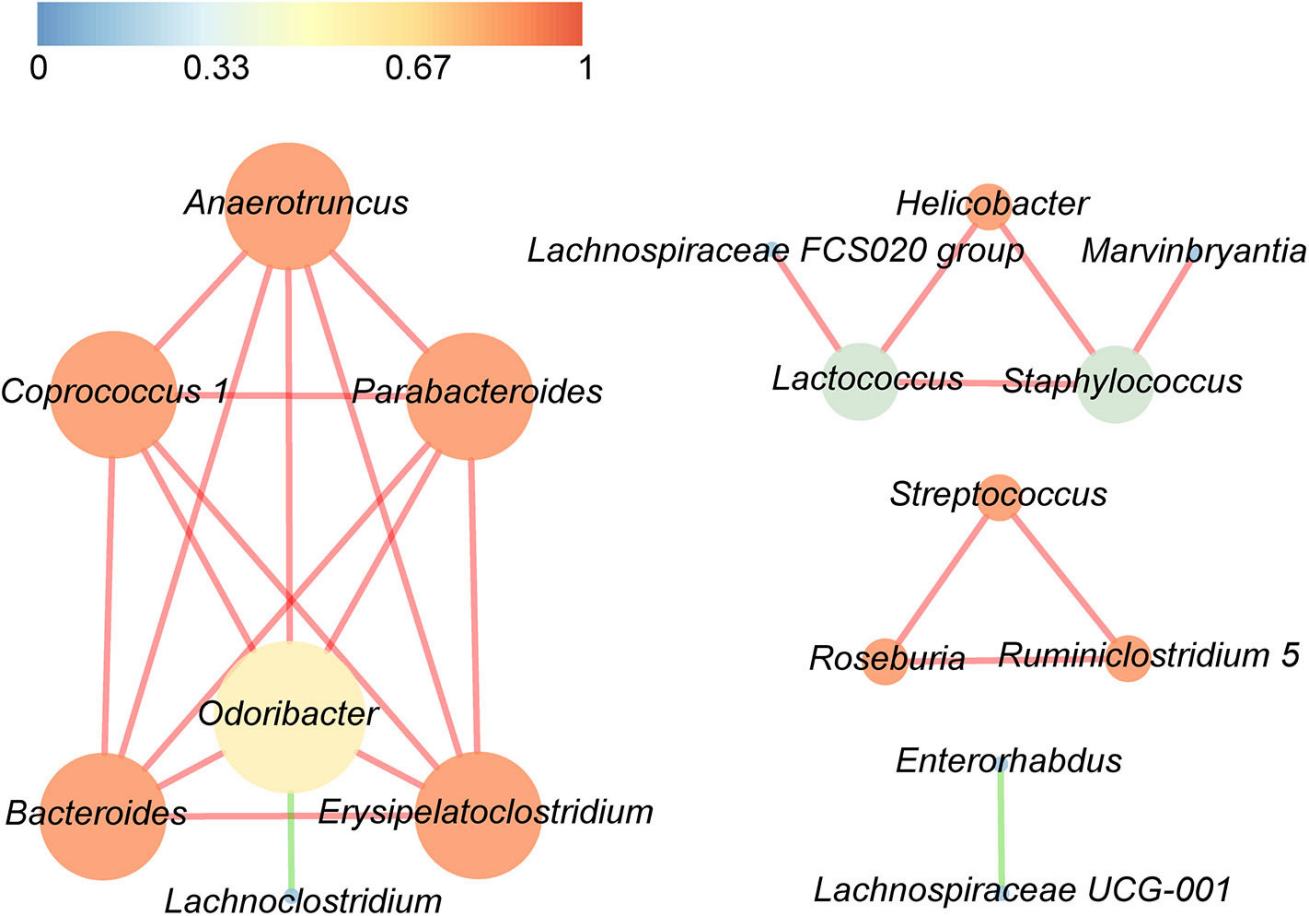
Significant relationships among the top 30 abundant bacterial genera were detected by Pearson correlation coefficient (*p* < 0.05). The nodes in the interaction network represent the dominant genus, the connection between them shows an association between the two genera, and the red line indicates a positive correlation, whereas the green line indicates a negative correlation. The size of a node is proportionally positive to the degree of the node, and the color of the node is related to the clustering coefficient, that is, the color gradient from dark colors to bright colors corresponds to the value of the aggregation coefficient from low to high.

Correlations between the top 30 dominant bacterial genera are demonstrated in Figure 5, with either a positive or a negative Pearson correlation coefficient (*p* < 0.05). The gut bacteria were divided into four clusters, and the most connected bacteria were *Anaerotruncus, Coprococcus 1, Parabacteroides, Bacteroides, Erysipelatoclostridium*, and *Odoribacter*, while *Odoribacter* had the maximum connections with other genera. It is interesting that significant negative correlations were only found in *Odoribacter* and *Lachnoclostridium*, as well as *Enterorhabdus* and *Lachnospiraceae UCG-001*, whereas the remaining bacteria were significantly positively related to each other.

### *S. japonicum* Infection-Induced Metagenome Changes

**Figure 6:**
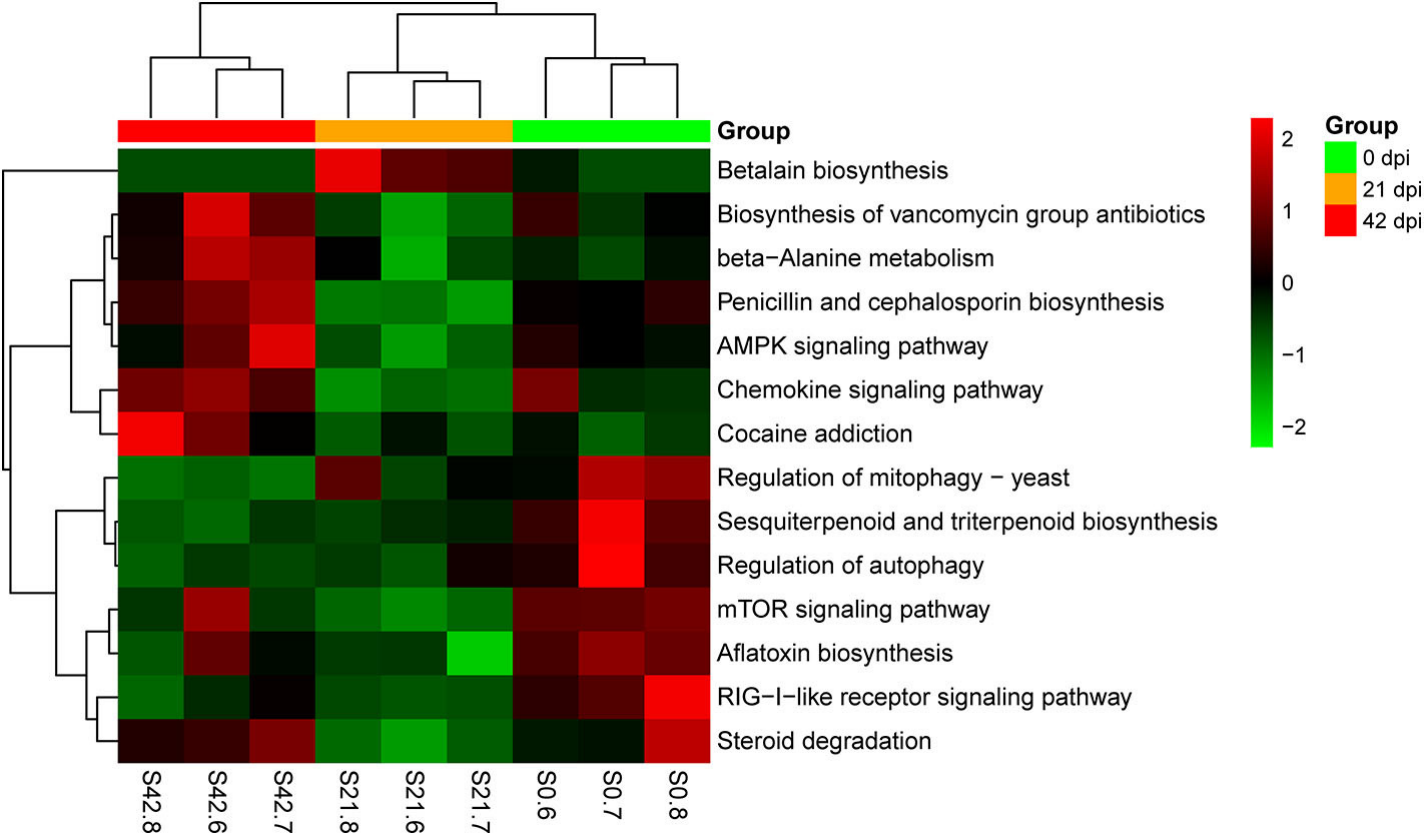
Hierarchical clustering heat map constructed using annotated metagenomic data on the basis of the Kyoto Encyclopedia of Genes and Genomes (KEGG). Samples are shown in columns, and KEGG pathways are shown in rows.

Metagenomic analysis was used in this study to identify genes of host gut microbiota involved in specific pathways or functions during *S. japonicum* infection. An average of 56,686,511 valid reads per sample was obtained after removing sequences of the host, and then a non-redundant gene catalog that contained 1,048,575 clusters assembled into bacterial genes was constructed for further annotation. Afterwards, annotated metagenomic data were obtained by mapping to KEGG orthologs (kos), and the ko number was implemented to correspond to the KEGG pathway to indicate which genes were associated with specific metabolic pathways or functions (Supplementary Table 4). A hierarchical clustering heat map constructed based on the results from analysis of KEGG at level 3 demonstrated a remarkable ability to discriminate between healthy mice and infected mice in 14 different KEGG pathways (Figure 6). The 0-dpi group showed seven enriched pathways including transport and catabolism (regulation of mitophagy—yeast ko04139, regulation of autophagy ko04140), signal transduction (mTOR signaling pathway ko04150), immune system (RIG-I-like receptor signaling pathway ko04622), metabolism of terpenoids and polyketides (sesquiterpenoid and triterpenoid biosynthesis ko00909), xenobiotic biodegradation and metabolism (steroid degradation ko00984), and biosynthesis of other secondary metabolites (aflatoxin biosynthesis ko00254) (*p* < 0.05). Additionally, signal transduction (AMPK signaling pathway ko04152), immune system (chemokine signaling pathway ko04062), metabolism of terpenoids and polyketides (biosynthesis of vancomycin group antibiotics ko01055), metabolism of other amino acids (beta-alanine metabolism ko00410), biosynthesis of other secondary metabolites (penicillin and cephalosporin biosynthesis ko00311), and substance dependence (cocaine addiction ko05030) were significantly perturbed in the 42-dpi group (*p* < 0.05), whereas biosynthesis of other secondary metabolites (betalain biosynthesis ko00965) was altered significantly in the 21-dpi group, with *p* < 0.05.

### Multivariate Statistical Analysis of Metabolite Profiling

In PCA score plots, QC samples that applied to test analytical repeatability and instrument performance and stability were tightly clustered, which demonstrated high reproducibility of the instrument (Supplementary Figure 4). Representative base peak intensity chromatograms of all types of specimens at seven time points are shown in Supplementary Figures 5–8. Differences in peaks and peak heights were observed among all groups, which indicated that the composition of metabolite profiles of BALB/c mice was changed during *S. japonicum* infection.

**Figure 7:**
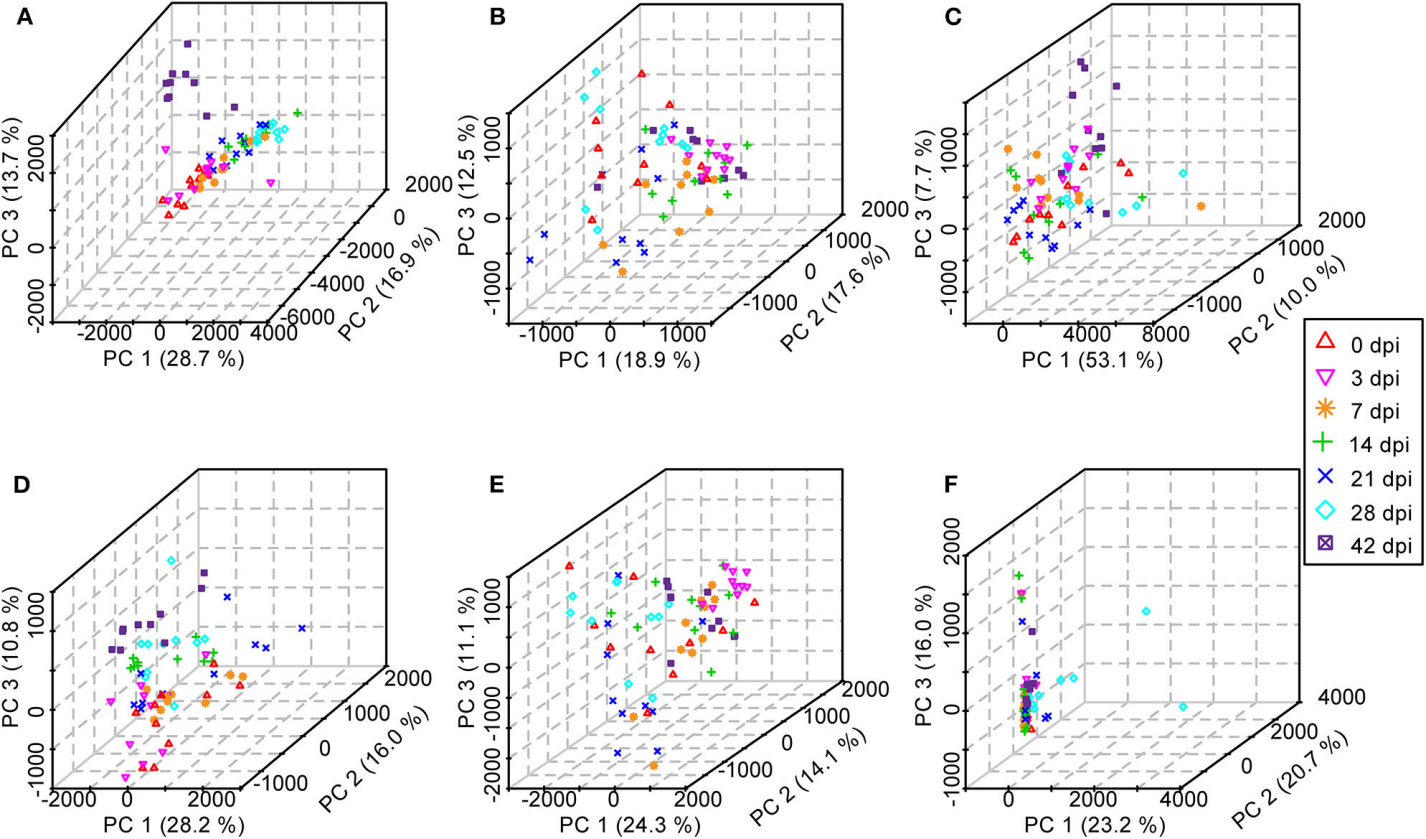
Three-dimensional score plots of principal component analysis from healthy mice and infected mice at different time points. Each point represents an individual. **(A)** Serum samples in positive electrospray ionization (ESI+) mode. **(B)** Urine samples in ESI+ mode. **(C)** Liver aqueous extracts in negative electrospray ionization (ESI-) mode. **(D)** Serum samples in ESI- mode. **(E)** Urine samples in ESI- mode. **(F)** Colon aqueous extracts in ESI- mode.

Initially, PCA, which is an unsupervised method of pattern recognition aiming to identify the overall clustering patterns and trends in a data set without considering groups, was implemented to obtain a global view and determine whether the metabolites from these seven groups of mice differed. Based on the top three principal components, the PCA results showed distinguished classifications between the control mice and the mice with different statuses of *S. japonicum* infection from serum and urine samples separately in both ESI modes, indicating that *S. japonicum* infection had significant effects on mouse metabolism (Figure 7). However, the 0-dpi group and early infection groups appeared to be partially overlapping, and the latter were closer than the late infection groups to the controls in the PCA score plots, whereas the most profound differences were found between the 0-dpi group and the late infection groups, which illustrate that the changes in metabolite profiles were miniscule at the early stage of infection and then became significant in the later stage. However, samples derived from liver and colon aqueous extracts demonstrated unclear classifications between healthy mice and infected mice of different time periods of *S. japonicum* infection with PCA in negative ion mode. This finding is evident in the PCA score plots in Figure 7F, which revealed that the effects of *S. japonicum* infection on mouse metabolism were not as evident in tissue as they were in body fluids.

**Figure 8:**
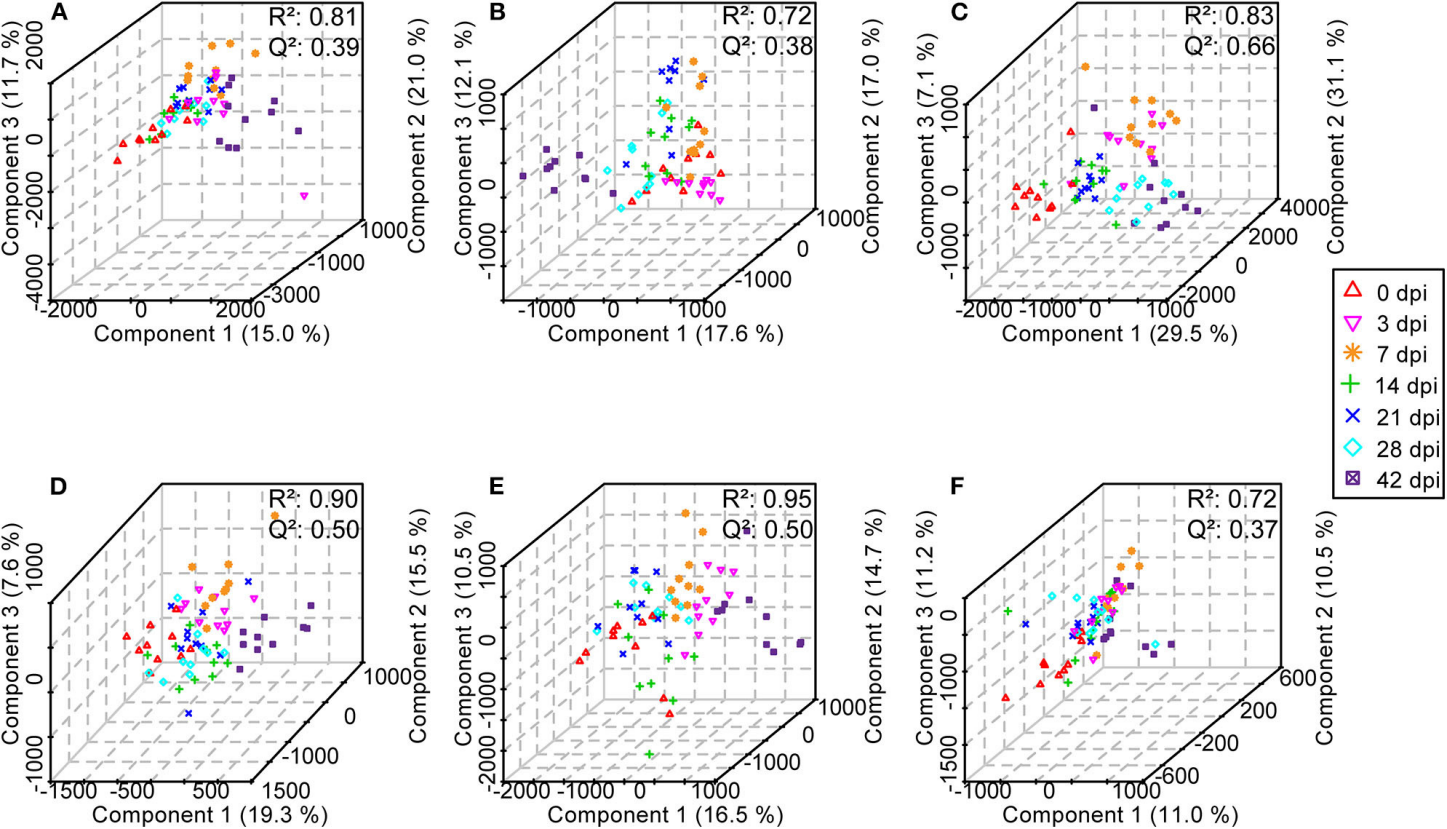
Three-dimensional score plots of partial least squares-discriminatory analysis from healthy mice and infected mice at different time points. Each point represents an individual. **(A)** Serum samples in positive electrospray ionization (ESI+) mode. **(B)** Urine samples in ESI+ mode. **(C)** Liver aqueous extracts in negative electrospray ionization (ESI-) mode. **(D)** Serum samples in ESI- mode. **(E)** Urine samples in ESI- mode. **(F)** Colon aqueous extracts in ESI- mode.

Subsequently, in order to enhance the separation among the different classes of samples, a supervised method, PLS-DA was performed. For serum, urine, and liver aqueous extracts, PLS-DA models were constructed for all time points of *S. japonicum* infection separately, and all groups could be readily clearly differentiated from each other by PLS1, PLS2, and PLS3 in both ESI modes; high values of *R*^2^ and *Q*^2^ of each model reflected the data stability and the good fit of the model parameters (Figure 8, Supplementary Table 5). These results revealed that the biochemical perturbations and the metabolic profiles of the infected groups were distinct from those of the uninfected group. Furthermore, notable separation was found between control mice and infected mice during the process of schistosomiasis. Nevertheless, colon aqueous extract samples from all groups still clustered together in the PLS-DA score plot.

Finally, OPLS-DA was applied to reduce the dimension and produce the clearest separation between two groups as well as identifying metabolites that drive group distinction with one predictive and one orthogonal component (Supplementary Figures 9–14). The S-plots obtained from OPLS-DA were used to find out the meaningful and reliable variables that were attributable to the separation between two groups; the ions farther from the origin in the plot represent higher VIP values and were selected as potential metabolite biomarkers (Supplementary Figures 9–14).

### Biomarker Identification and Analysis

**Figure 9:**
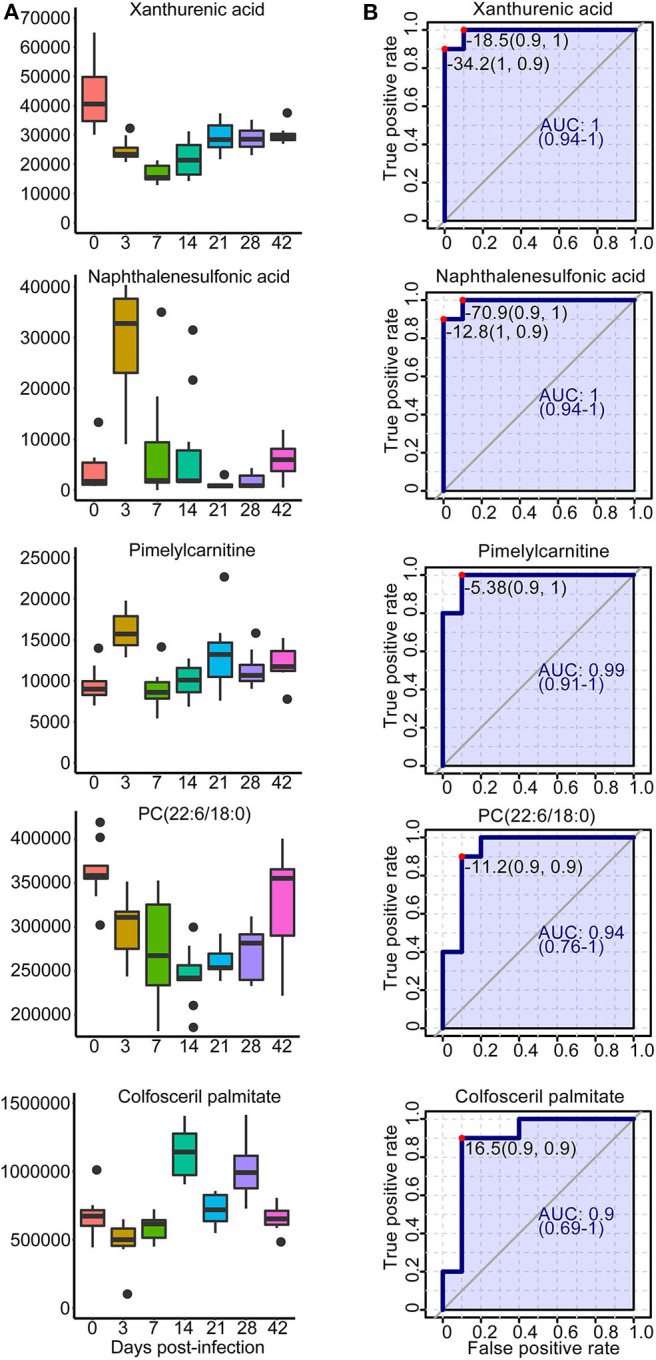
Overview of selected representative metabolites from serum and urine associated with schistosomiasis. **(A)** Box plots showing the abundance of selected biomarkers. Each column represents a time point. **(B)** The receiver operating characteristic curve for selected biomarkers. The x-axis represents the specificity, while the y-axis represents the sensitivity.

A total of 42 unique compounds were identified in serum extracts, 53 unique compounds were identified in urine samples, and 24 unique compounds were identified from liver and colon aqueous extracts on the basis of the S-plot, VIP scores >2, *p* < 0.05, and QC samples' CV <30 (Supplementary Table 6). The majority of these metabolites were lipids, glucose, organic acids, nucleic acids, and amino acids. As shown in Supplementary Figure 15, hierarchical cluster analysis demonstrated the trends of significantly identified compounds for seven time points from all individuals. Most of the lipids were downregulated persistently during *S. japonicum* infection, while some phospholipids were upregulated in medium-term infection or late infection. However, several phospholipids were altered in only early- and medium-term infection, with downregulation. In addition, glucose and some organic acids were increased at 3 dpi but dropped to the uninfected levels after that. We picked out five potential biomarkers related to the early diagnosis of schistosomiasis (at 3 dpi) based on VIP value >5 and the value of the area under the ROC curve (AUC) >0.9. Phosphatidylcholine (PC) (22:6/18:0) and colfosceril palmitate in serum as well as xanthurenic acid, naphthalenesulfonic acid, and pimelylcarnitine in urine demonstrated promising diagnostic potential between the uninfected group and the 3-dpi group, with an AUC value of 0.9–1, while xanthurenic acid and naphthalenesulfonic acid exhibited the most discriminatory power since the sensitivity and the specificity were both 100% (Figure 9B). Therefore, xanthurenic acid and naphthalenesulfonic acid may be the most powerful targets for early diagnosis of schistosomiasis. The abundance of xanthurenic acid, PC (22:6/18:0), and colfosceril palmitate were significantly reduced at 3 dpi, whereas naphthalenesulfonic acid and pimelylcarnitine levels rose extremely at 3 dpi (Figure 9A).

### MSEA and Pathway Analysis

**Figure 10:**
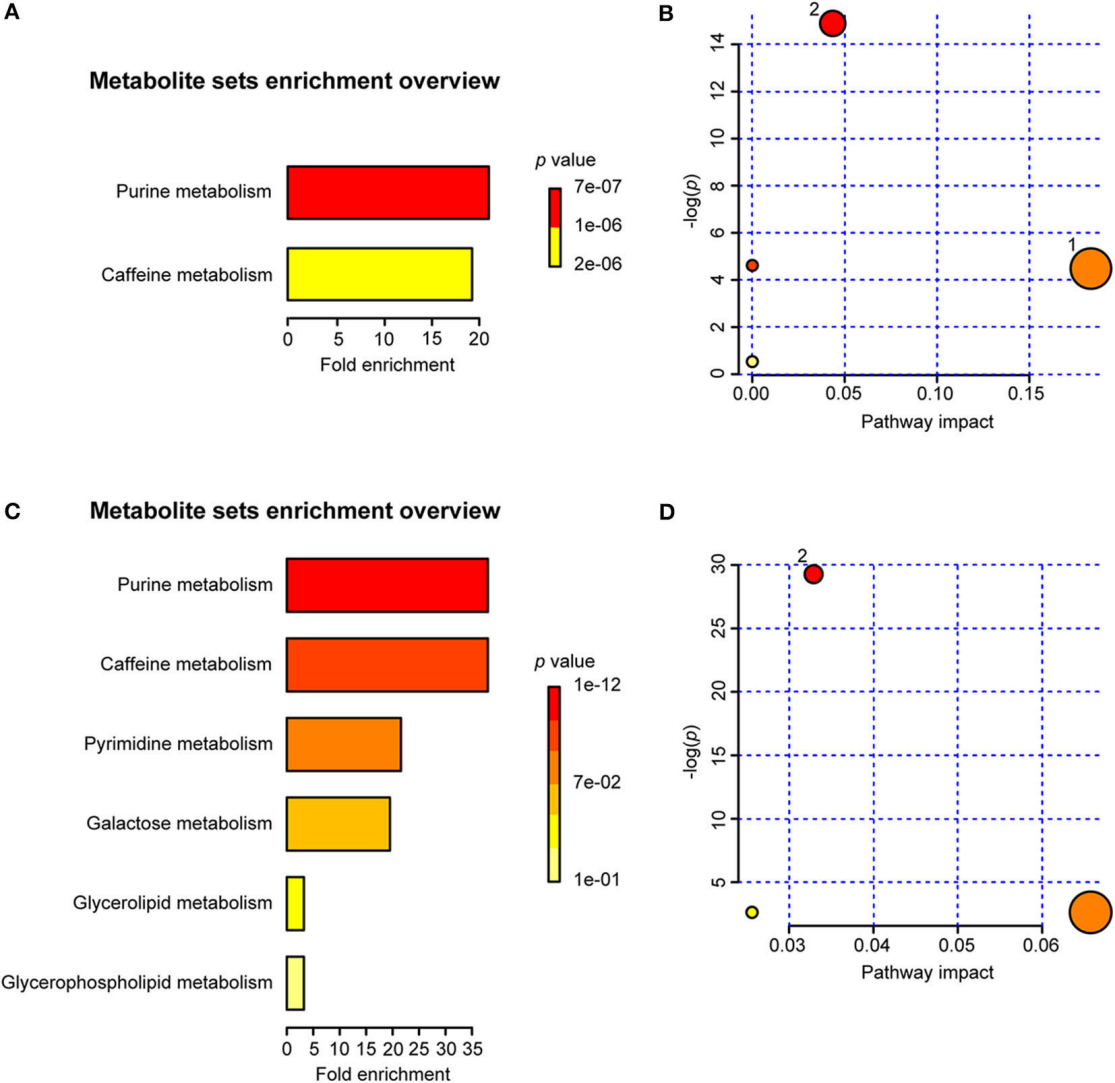
Summary of metabolite set enrichment analysis (MSEA) and pathway analysis. **(A)** Summary plot for MSEA in serum is ranked according to Holm *p*-value. **(B)** Pathway analysis of serum metabolites shows key nodes in metabolic pathways that have been significantly changed during an infection. The x-axis represents increasing metabolic pathway impact from pathway topology analysis, whereas the y-axis represents unadjusted *p*-value by pathway enrichment analysis. Greater pathway enrichment and higher pathway impact values are exhibited in larger sizes and darker colors, respectively. **(C)** Summary plot for MSEA in liver aqueous extracts is ranked according to Holm *p*-value. **(D)** Pathway analysis of liver aqueous metabolite shows key nodes in metabolic pathways that have been significantly changed during an infection. 1, glycerophospholipid metabolism; 2, purine metabolism.

The MSEA results indicated that two sets of identified metabolites extracted from serum (Figure 10A) and six sets of identified metabolites in liver aqueous extracts (Figure 10C) were different between the uninfected group and the infected groups; among these, purine metabolism, caffeine metabolism, pyrimidine metabolism, and galactose metabolism were significantly enriched (*p* < 0.05). Moreover, the metabolites were grouped based on the KEGG database by pathway analysis, while purine metabolism (ko00230), sphingolipid metabolism (ko00600), and glycerophospholipid metabolism (ko00564) were significantly related to the process of schistosomiasis (*p* < 0.05) (Figures 10B,D). Among pathways of serum metabolites, glycerophospholipid metabolism and purine metabolism showed the impact factors of 0.18 and 0.04, respectively (Figure 10B), while purine metabolism demonstrated the impact factor of 0.03 in pathways of liver aqueous metabolites (Figure 10D), which indicated that these two pathways were disturbed mostly during *S. japonicum* infection.

### Relationships Between Host Metabolome and Gut Microbiome

**Figure 11:**
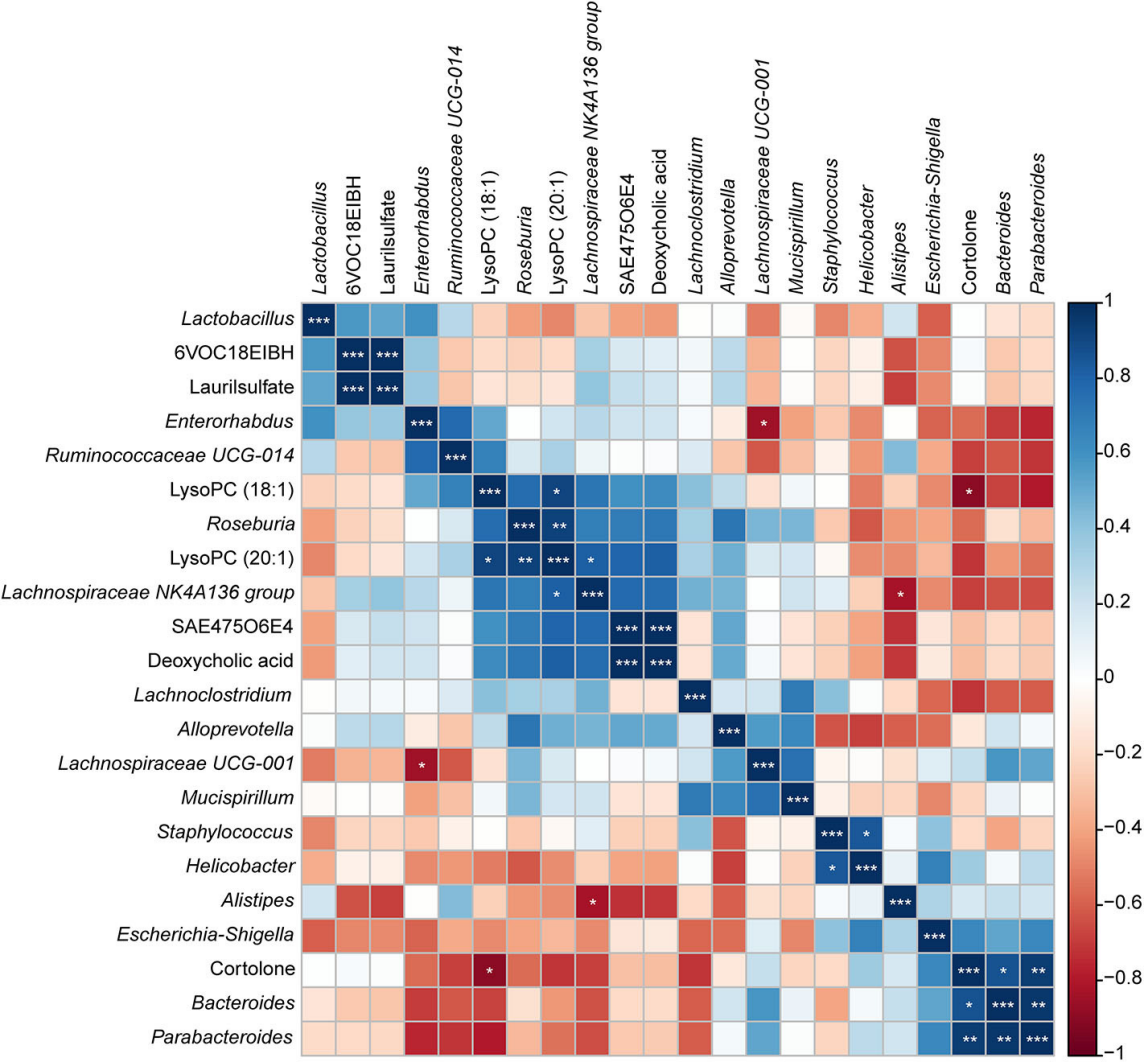
Correlation plot showing the relationship between the altered metabolites from the colon and the top 15 abundant gut bacteria. The correlations between them are exhibited by colors; blue indicates a positive correlation, red indicates a negative correlation, and a darker color illustrates a stronger correlation (*p < 0.05, **p < 0.01, ***p < 0.001).

To investigate the functional correlation between the altered metabolites from the colon and fecal flora alterations, correlation analysis was conducted by calculating the Pearson's correlation coefficient. As shown in Figure 11, clear correlations between altered metabolic profiles and gut microbiome were observed; however, more metabolites were negatively correlated with fecal flora, whereas two compounds exhibited significant positive correlations with some bacterial groups (*p* < 0.05). Of particular note is that LysoPC (20:1), which decreased in the colon of *S. japonicum*-infected BALB/c mice, was positively correlated with the reduction of *Lachnospiraceae NK4A136* group and *Roseburia*. Additionally, cortolone, which increased in the colon of *S. japonicum*-infected BALB/c mice, was positively correlated with the elevation of *Bacteroides* as well as *Parabacteroides*. In summary, *S. japonicum* infection induced significant perturbation in both the gut microbiome and the metabolomic profile of the host, which were interactive during the process of schistosomiasis.

## Discussion

Parasitic infection can impact the gut microbiota composition of the host, and the underlying mechanism is its effect on the host immune system, which could break the balance between the gut microbiome and the host that has already been established (40). In this study, *16S* rRNA sequencing was applied to demonstrate the dynamic alteration in the gut microbiome of BALB/c mice in response to *S. japonicum* infection and to identify bacteria that are crucial in the complex host*-*parasite interplay. The result of diversity analysis showed an overall reduction in alpha diversity and a relative increase in beta diversity of host gut bacteria after *S. japonicum* infection; beta diversity analysis also demonstrated strong associations between the gut microbiota composition and stage of infection, especially in the late stage, which was in conformity with previous studies of *S. japonicum* and *S. mansoni* (25, 41). As we have known, the schistosomula enter the venous blood vessels and are transported to the lungs where they become lung schistosomula at 3 dpi. At 7 dpi, the schistosomula enter the arterial circulation and then migrate to the mesenteric veins of the liver and become mature a week later. At 21 dpi, the worms migrate to the mesenteric veins of the bowel, and gametes of both the female and the male worms occur. Then, the female worm produces immature eggs at 28 dpi. At 42 dpi, mature eggs deposit in the liver of the host through the bloodstream or pass through the intestinal wall and are excreted in the feces. Thus, the gut microbiome community structures of the host are altered variously by *S. japonicum* infection due to the varying parasitic sites of the worm at different time points, and the gut microbiota of each individual respond differently according to the degree of homeostasis disruption. While the decrease in richness of the gut microbiota is harmful to the healthy individual who is infected by the parasite because the gut microbiome is crucial in providing a protective environment (42), for the individual with autoimmune diseases, such as Crohn's disease, ulcerative colitis (UC), and coeliac disease, parasites may actively contribute to reinstating gut homeostasis with quantitative and qualitative modifications of the gut microbiota, which can profoundly influence immune cell development and function in the intestine (43–46).

Firmicutes, Bacteroidetes, Proteobacteria, and Deferribacteres, which are common in the mouse (47), were also the dominant gut bacterial phyla in both infected and uninfected BALB/c mice, but no statistically significant differences were observed during the infection. Similarly, previous research has shown that Firmicutes decreased relatively and Bacteroidetes and Proteobacteria increased relatively in response to *S. japonicum* infection in C57BL/6 mice, whereas the same variations were not found in BALB/c mice (25). These results illustrated that the alteration in gut microbiome composition after *S. japonicum* infection was closely related to the host itself. The abundance of some prevalent genera, including *Staphylococcus, Parabacteroides, Alistipes, Roseburia*, and *Ruminococcaceae UCG-014*, was changed significantly during the infection. Both *Parabacteroides* and *Alistipes* are members of Bacteroidetes and were relatively abundant in response to infection, while members of Firmicutes, *Roseburia*, and *Ruminococcaceae UCG-014* were decreased after the infection, which indicated that although no significant differences were found at the phylum level, significant differences were observed at the genus level, and the change trend of different genera from the same phylum was consistent in the course of the infection. Earlier studies have illustrated that *Alistipes* can produce anti-inflammatory metabolites, which would promote the differentiation of anti-inflammatory Treg/Tr1 cells in the gut of mice, and *Alistipes* can also protect mice suffering from the effects of dextran sulfate sodium (48, 49). Hence, depletion of *Alistipes* was found in *Salmonella*-infected mice, porcine epidemic diarrhea virus-infected piglets, and chronic hepatitis B patients (50–52). Nevertheless, *Alistipes* was reported to result in the increase of trimethylamine N-oxide and the decrease of short-chain fatty acids (SCFA) production, which led to the destruction of the intestinal barrier (51, 53). The enhanced abundance of *Alistipes* during *S. japonicum* infection may be caused by the stress reaction of the hosts. Furthermore, as probiotic-type bacteria, which are able to prevent pathogen infection, a reduction in the diversity and the abundance of *Parabacteroides* was observed in mice infected with intestinal helminth parasites in an earlier study (54), which was found at only 7 dpi of *S. japonicum*; however, the increased abundance of *Parabacteroides* after 7 dpi might be a consequence of stress response because some bacteria belonging to *Parabacteroides* were involved in the metabolism of amino acid. Consistently, previous studies have suggested that *Roseburia*, one kind of SCFA-producing bacteria that is essential for maintaining the gut function of humans and animals (55), was significantly decreased both in chronic kidney disease and in end-stage renal disease patients (56, 57), so the intestinal homeostasis was broken. Similarly, some members of Ruminococcaceae were butyrate producers, and the abundance of *Ruminococcaceae UCG-014* reduced in a UC carcinogenesis model and even in a hypertriglyceridemia-related acute necrotizing pancreatitis model (58, 59). Interestingly, *Staphylococcus*, a classical non-enteric pathogen, was also found in the gut microbiome and increased in cystic fibrosis patients (60, 61), which was evident at 21 dpi of *S. japonicum*.

Using LEfSe, we noted that *Alistipes* (phylum Bacteroidetes) showed great abundance at 7 dpi and that another member from Bacteroidetes, *Parabacteroides*, was significantly associated with 14 dpi of *S. japonicum*. Furthermore, LEfSe identified a greater differential abundance of *Staphylococcus* (phylum Firmicutes) at 21 dpi, before the female adults laid eggs, while the greater differential abundance of Gammaproteobacteria was highlighted by LEfSe at 28 dpi, after the female adults laid eggs. Conversely, a significant over-representation of the class Gammaproteobacteria (phylum Proteobacteria) was identified in *Clostridium difficile* infection patients and children with acute diarrhea (62, 63). However, since we did not study bacteria with low abundance, we cannot draw a more precise conclusion based on the results of LEfSe. Correlation analysis of the top 30 dominant genera in the course of *S. japonicum* infection illustrated that most genera were positively related to each other, which showed that the co-occurrence relation was general in the gut microbiome for those who would work together even though they may belong to different phyla. Regardless, co-exclusion relationships were observed in *Odoribacter* (phylum Bacteroidetes) and *Lachnoclostridium* (phylum Firmicutes) as well as in *Lachnospiraceae UCG-001* (phylum Firmicutes) and *Enterorhabdus* (phylum Actinobacteria). *Odoribacter* and *Lachnospiraceae UCG-001* became more abundant, while the others became relatively less abundant during *S. japonicum* infection.

The results of the *16S* rRNA gene-based analysis clarified the dynamic changes in the host gut microbiome in response to *S. japonicum* infection and the interactions between dominant genera. Subsequently, metagenomics sequencing was performed to search for altered pathways or functions at 0, 21, and 42 dpi of *S. japonicum*, which might enable us to generate hypotheses about the mechanisms underlying the infection. Consistent with the alterations in the gut microbiota, the functional metagenome was also changed significantly. In particular, the only ko category significantly enriched among the microbiome of BALB/c mice at 21 dpi was the biosynthesis of betalain, and the involved gene was tyrosinase (K00505), a kind of oxidoreductase. Betalain biosynthesis plays a crucial part in response to osmotic adjustment and salt resistance, and betaine is vital to enhance stress resistance (64), so the activation of betalain biosynthesis was helpful to the gut microbiome to resist stress while *S. japonicum* migrates to the mesenteric veins. Other particularly notable signaling pathways associated with *S. japonicum* infection at 42 dpi were the AMPK and the chemokine signaling pathways. The former was activated by calcium/calmodulin-dependent protein kinase exclusion β (CaMKK β, K07359), one of the upstream kinases, which reflected the elevated AMP/ATP ratio in response to the infection. Once activated, AMPK would lead to a concomitant inhibition of energy-consuming biosynthetic pathways, including the synthesis of glycogen and cholesterol. Chemokines play an important role in protective host response, and the upregulation of the chemokine signaling pathway at 42 dpi showed that the gut microbiota was sensitive to the infection and chemokine receptors were activated to produce an inflammatory immune response. In summary, we hypothesize that the gut microbiota in the host provide signals that resist the parasites and enhance the immune response of the host. These findings from the functional metagenome analysis offered a more detailed view of specific microbiome-encoded functions that are associated with *S. japonicum* infection and may facilitate further studies on the interaction between specific genes.

To describe the metabolic profile of BALB/c mice after *S. japonicum* infection, an untargeted UPLC–MS/MS-based high-resolution metabolomics analysis was performed in the present study. Both PCA and PLS-DA revealed differences between uninfected and infected mice, and the differences increased over time; however, more obvious variances were observed in body fluids than in tissues, which indicated that body fluids were affected more severely than cells during *S. japonicum* infection and that is why more differential metabolites were obtained in the former than in the latter. This result is expected because *S. japonicum* does not parasitize the host cell.

In response to *S. japonicum* infection, the significantly different metabolites were lipids, and the result is consistent with the prior study on *S. japonicum* and *T. brucei rhodesiense* (*T. b. rhodesiense*) (2, 16). Lipids are the major constituents of membranes and are important in reserving energy; they are also highly biologically active metabolites that are involved or even play a part in signaling and a range of inflammatory processes (65). As the major structural lipids that form cellular membranes, phospholipids participate in the regulation of nutrient transport as well as toxic host-cell effector molecules; they are synthesized to support the growth of cells in the course of infection, and phospholipid biosynthetic pathways are the targets of drugs (66–68). Our results demonstrated that most of the altered lipids belonged to glycerophospholipids, which are structural components of biological membranes (69), followed by sphingolipids and diacylglycerol. The majority of glycerophospholipids were significantly reduced in response to the infection, such as PC, phosphatidylethanolamine (PE), phosphatidylserine, phosphatidylinositol, and LysoPC. However, the concentration of some PCs was elevated in *S. japonicum*-infected mice, which was different from the results in *S. haematobium*-infected patients, where high levels of PC and PE were found (70). It is believed that the abundance of PC and PE in bladder endothelial cells may be one of the mechanisms for inducing cancer in chronic urogenital schistosomiasis (70); nevertheless, the different variations presented in PC after *S. japonicum* infection remain unclear, and future studies should address this question.

An endogenous phospholipid, LysoPC, which is derived from PC, has various stimulating or modulating effects on immune cells and possesses pro-inflammatory activities as well as anti-inflammatory properties; a heightened level of LysoPC in the plasma was considered as a marker for cell membrane injury (71, 72). Besides that, LysoPC participates in the elimination of dead eukaryotic and prokaryotic cells and controlling bacterial growth during infection (72, 73). Earlier studies have proven that LysoPC has direct antibacterial activities against methicillin-resistant *Staphylococcus aureus* as well as the ability to enhance neutrophil antimicrobial ability to remove ingested bacteria (74–76); however, our results illustrated that LysoPC does not inhibit all bacteria because LysoPC (20:1) was positively correlated with *Lachnospiraceae NK4A136* group and *Roseburia*, the genera that are beneficial to the host during *S. japonicum* infection.

It cannot be ignored that some sphingomyelin (SM) species were reduced after infection, while the concentration of ceramide (Cer) was elevated, especially at the late stage of infection; the variation trend of SM and Cer was also observed in *T. b. rhodesiense* human African trypanosomiasis patients (16). Sphingolipids not only are components of the membrane that are able to protect the cell from harmful environmental factors but also are involved in cellular signaling as a second messenger (77, 78); the significant observations in sphingolipids (SM and Cer) may offer the basis and foundation for revealing the resistance mechanism between hosts and *S. japonicum* since infection can trigger the generation of Cer, which is generated in part by sphingomyelinase enzymes, leading to cell autophagy and apoptosis, additional pro-inflammatory cytokines, and chemokine synthesis, as well as other metabolic disorders (16, 79, 80). It is more interesting to note that the metagenomic analysis revealed that the chemokine signaling pathway was upregulated at 42 dpi, which suggests that inflammatory effects existed in gut bacteria also; the host and the gut microbiota collaborate with each other to eliminate the parasites.

Surprisingly, the levels of two kinds of carnitine species (acetylcarnitine and oleoylcarnitine) were significantly lower in infected mice than in uninfected mice, while another two kinds of carnitine species (hydroxyisovaleroyl carnitine and pimelylcarnitine) were increased at 3 dpi. Carnitine plays a crucial role in fat metabolism and energy production in mammals, and it can support the production of CD4^+^ and CD8^+^ T cells during infection (81, 82); a prior study has shown that the levels of carnitine and several acylcarnitines were elevated in *S. japonicum*-infected *Microtus fortis* (*M. fortis*) and C57BL/6 mice, but the phenomenon was more obvious in *M. fortis* (21). Hence, we suggest that host responses to *S. japonicum* infection vary and that the protective reaction against *S. japonicum* is relatively less strong in BALB/c mice.

Pathway analysis illuminated that both sphingolipid metabolism and glycerophospholipid metabolism were altered in the process of schistosomiasis, which is consistent with an earlier study of *S. haematobium* (70). In fact, sphingolipids and their derivatives have recently presented as promising drug targets for controlling infectious and inflammatory disease; however, how sphingolipid-mediated pathologies and how the host modifies sphingolipid metabolism to benefit itself remain unclear, and a better understanding of these mechanisms may provide new insights into new therapeutic strategies (83).

Nevertheless, because of the absence of age-matched uninfected BALB/c mice in this study, the alterations of gut microbiome and metabolites could be partially related to the age effect rather than the progression of *S. japonicum* infection alone.

## Conclusions

In the present study, we applied *16S* rRNA gene sequencing combined with a metagenomic sequencing approach and UPLC–MS metabolic profiling to highlight three aspects of the interrelationships between *S. japonicum*, the gut microbiome, and metabolites. We demonstrated that both the gut microbiome and the metabolites were significantly altered in *S. japonicum*-infected BALB/c mice; moreover, they were also associated with the time course of *S. japonicum* infection. In response to *S. japonicum* infection, not only the richness and diversity of gut microbiota were decreased but also the composition of the microbiota which differed obviously from that present during the uninfected status. In summary, the abundance of some bacteria that could produce SCFA was decreased, while those of some opportunistic pathogens that could raise the risk of infections were increased. In addition, metagenomic analysis revealed that the AMPK and chemokine signaling pathways were significantly perturbed after infection. The metabolic biomarkers that we identified in this study were found in serum or urine, with little or no invasiveness, and could distinguish *S. japonicum* infection from non-infection at 3 dpi with high sensitivity and specificity. Additionally, alterations in glycerophospholipid and purine metabolism were discovered in *S. japonicum* infection. As a result, these findings may provide a novel understanding of the mechanisms during schistosomiasis development regarding aspects of the gut microbiome and metabolites and facilitate the discovery of new targets for early diagnosis and prognosis. Nevertheless, further validations of potential biomarkers in human populations are essential, and the exact mechanisms of interactions between *S. japonicum*, the gut microbiome, and metabolites also await future research.

## Data Availability Statement

The sequencing data of 16S rRNA gene and metagenome have been deposited in the NCBI Sequence Read Archive under the project number PRJNA602960 and PRJNA602878.

## Ethics Statement

The animal study was reviewed and approved by the Animal Care and Use Committee of Sun Yat-sen University.

## Author Contributions

ZL conceived and designed the study. ZL, YH, and JC drafted the manuscript. YH and JC carried out the experiments. YX, HoZ, PH, YM, MG, SC, and HaZ participated in data analysis. HaZ participated in study design, technological guidance, and coordination. All authors contributed to the article and approved the submitted version.

## Conflict of Interest

The authors declare that the research was conducted in the absence of any commercial or financial relationships that could be construed as a potential conflict of interest.

## Footnotes

^1^http://huttenhower.org/galaxy

^2^http://www.ncbi.nlm.nih.gov/assembly/GCF_000001635.23

^3^http://soap.genomics.org.cn/

^4^https://github.com/hyattpd/Prodigal

^5^http://www.bioinformatics.org/cd-hit/

^6^http://blast.ncbi.nlm.nih.gov/Blast.cgi

^7^http://www.genome.jp/kegg/pathway.html (KEGG)

^8^http://www.metaboanalyst.ca

^9^http://www.hmdb.ca

^10^http://metlin.scripps.edu

## Supplementary Material

Supplementary Figure 1 A flower plot showing unique and common operational taxonomic units among all samples.

Supplementary Figure 2 Rarefaction plots of *16S* rRNA gene sequences obtained from fecal samples across multiple time points based on alpha diversity analyses. Lines represent the means, and the standard deviations are shown by error bars. **(A)** Observed species. **(B)** Chao1. **(C)** Shannon–Wiener index. **(D)** Phylogenetic diversity index.

Supplementary Figure 3 Principal coordinate analysis (PCoA) plots showing beta diversity differences on the basis of *16S* rRNA gene sequencing during *S. japonicum* infection. Figures were calculated using weighted UniFrac distance, and each point represents an individual. **(A)** Two-dimensional PCoA plot. **(B)** Three-dimensional PCoA plot.

Supplementary Figure 4 Three-dimensional score plots of principal component analysis from quality control and samples. **(A)** Serum samples in positive electrospray ionization (ESI+) mode. **(B)** Urine samples in ESI+ mode. **(C)** Liver aqueous extracts in negative electrospray ionization (ESI-) mode. **(D)** Serum samples in ESI- mode. **(E)** Urine samples in ESI- mode. **(F)** Colon aqueous extracts in ESI- model.

Supplementary Figure 5 Base peak intensity chromatograms of serum samples from different time points in ESI+ mode **(A)** and ESI- mode **(B)** as analyzed by quadrupole-time-of-flight mass spectrometry with a full scan.

Supplementary Figure 6 Base peak intensity chromatograms of urine samples from different time points in ESI+ mode **(A)** and ESI- mode **(B)** as analyzed by quadrupole-time-of-flight mass spectrometry with a full scan.

Supplementary Figure 7 Base peak intensity chromatograms of liver aqueous extracts from different time points in ESI- mode as analyzed by quadrupole-time-of-flight mass spectrometry with a full scan.

Supplementary Figure 8 Base peak intensity chromatograms of colon aqueous extracts from different time points in ESI- mode as analyzed by quadrupole-time-of-flight mass spectrometry with a full scan.

Supplementary Figure 9 Orthogonal partial least squares-discriminatory analysis (OPLS-DA) of serum extracts in ESI+ mode at different stages of *Schistosoma japonicum* infection. Each point represents one sample in score plots. OPLS-DA loadings S-plot combined with the covariance and the correlation loading profile indicate the variable importance. Each point represents a variable.

Supplementary Figure 10 Orthogonal partial least squares-discriminatory analysis (OPLS-DA) from serum extracts in ESI- mode at different stages of *Schistosoma japonicum* infection. Each point represents one sample in score plots. OPLS-DA loadings S-plot combined with the covariance and the correlation loading profile indicate the variable importance. Each point represents a variable.

Supplementary Figure 11 Orthogonal partial least squares-discriminatory analysis (OPLS-DA) from urine extracts in ESI+ mode at different stages of *Schistosoma japonicum* infection. Each point represents one sample in score plots. OPLS-DA loadings S-plot combined with the covariance and the correlation loading profile indicate the variable importance. Each point represents a variable.

Supplementary Figure 12 Orthogonal partial least squares-discriminatory analysis (OPLS-DA) from urine extracts in ESI- mode at different stages of *Schistosoma japonicum* infection. Each point represents one sample in score plots. OPLS-DA loadings S-plot combined with the covariance and the correlation loading profile indicate the variable importance. Each point represents a variable.

Supplementary Figure 13 Orthogonal partial least squares-discriminatory analysis (OPLS-DA) from liver aqueous extracts in ESI- mode at different stages of *Schistosoma japonicum* infection. Each point represents one sample in score plots. OPLS-DA loadings S-plot combined with the covariance and the correlation loading profile indicate the variable importance. Each point represents a variable.

Supplementary Figure 14 Orthogonal partial least squares-discriminatory analysis (OPLS-DA) from colon aqueous extracts in ESI- mode at different stages of *Schistosoma japonicum* infection. Each point represents one sample in score plots. OPLS-DA loadings S-plot combined with the covariance and the correlation loading profile indicate the variable importance. Each point represents a variable.

Supplementary Figure 15 Heat map visualization for the significantly different identified metabolites at different time points. Each column represents a sample, and each row represents a metabolite. The scaled intensity of the metabolites is reflected by the color key; higher intensity levels, when compared to the mean metabolite intensity value, are colored red, while lower intensity levels are colored green. Branch height represents the similarity between two metabolites, and metabolites with the shortest vertical difference indicate the closest connection.

Supplementary Table 1 Operational taxonomic unit tables and taxonomic classifications of the *16S* rRNA gene.

Supplementary Table 2 One-way ANOVA of relative abundances of gut microbiome constituents at the phylum level.

Supplementary Table 3 One-way ANOVA of relative abundances of gut microbiome constituents at the genus level.

Supplementary Table 4 KEGG annotation of metagenomic data.

Supplementary Table 5 Q^2^ and R^2^ values are provided to show partial least squares-discriminatory analysis (PLS-DA) classification performance with different number of components.

Supplementary Table 6 Statistically significantly changed metabolites identified in serum samples, urine samples, liver aqueous extracts and colon aqueous extracts (*p* < 0.05).
