# Supplementary figures and images for: Alterations of Gut Microbiome and Metabolite Profiling in Mice Infected by Schistosoma japonicum
Source: Front Immunol. 2020 Oct 8;11:569727. doi: 10.3389/fimmu.2020.569727 (PMC7580221; doi:10.3389/fimmu.2020.569727)

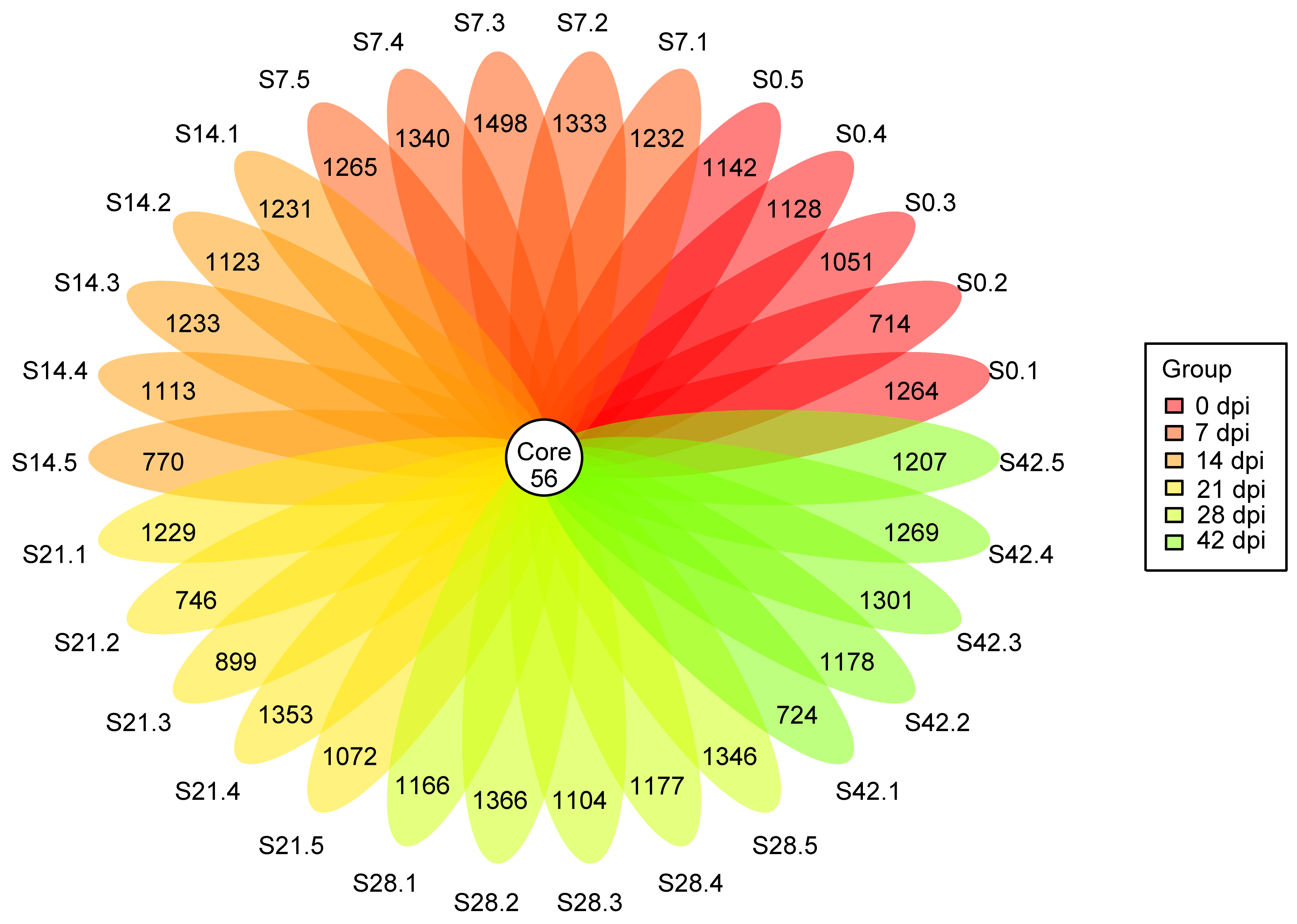

Supplement: Supplementary Figure 1 — A flower plot showing unique and common operational taxonomic units among all samples. [file Image_1.TIF]

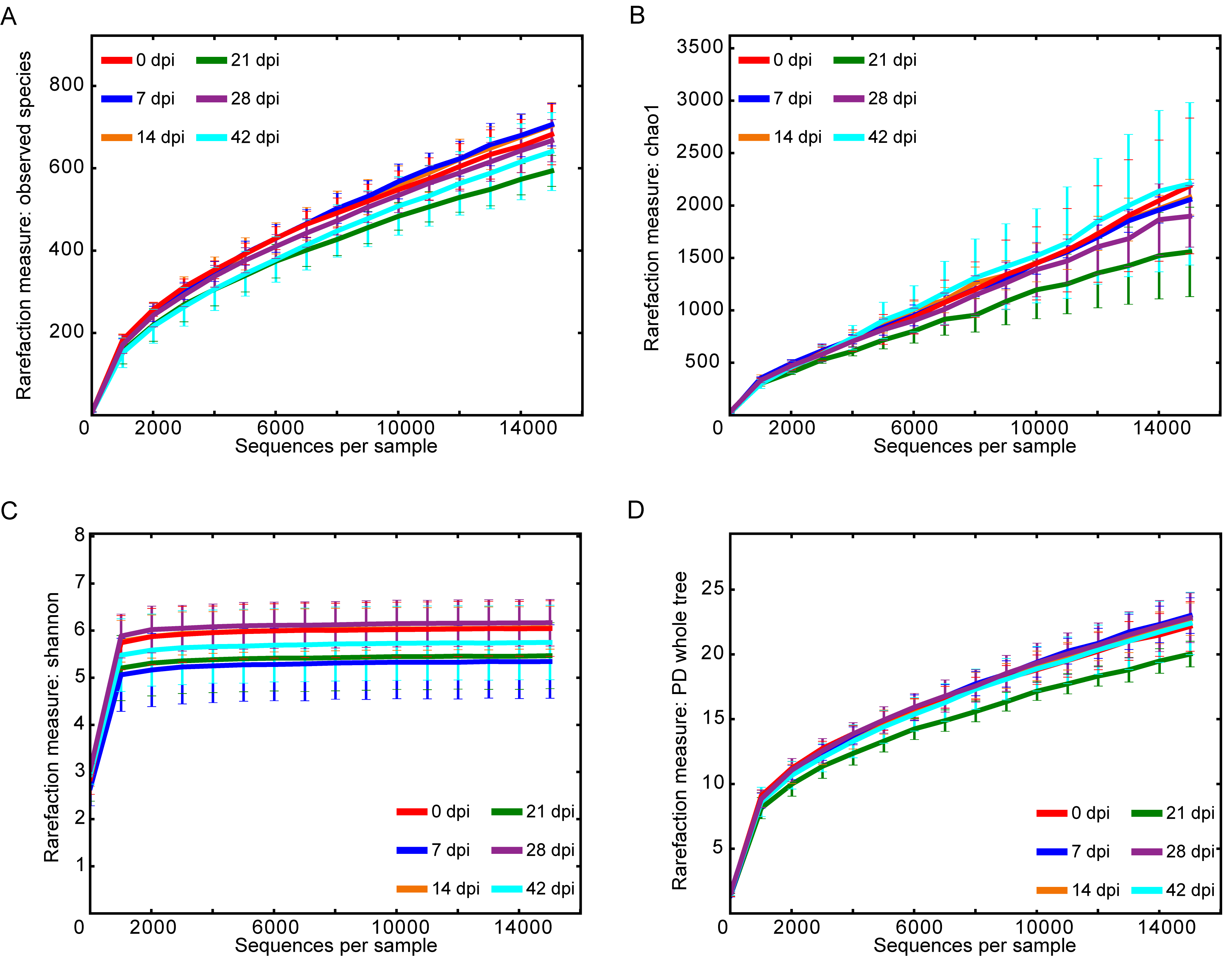

Supplement: Supplementary Figure 2 — Rarefaction plots of 16S rRNA gene sequences obtained from fecal samples across multiple time points based on alpha diversity analyses. Lines represent the means, and the standard deviations are shown by error bars. (A) Observed species. (B) Chao1. (C) Shannon–Wiener index. (D) Phylogenetic diversity index. [file Image_2.TIF]

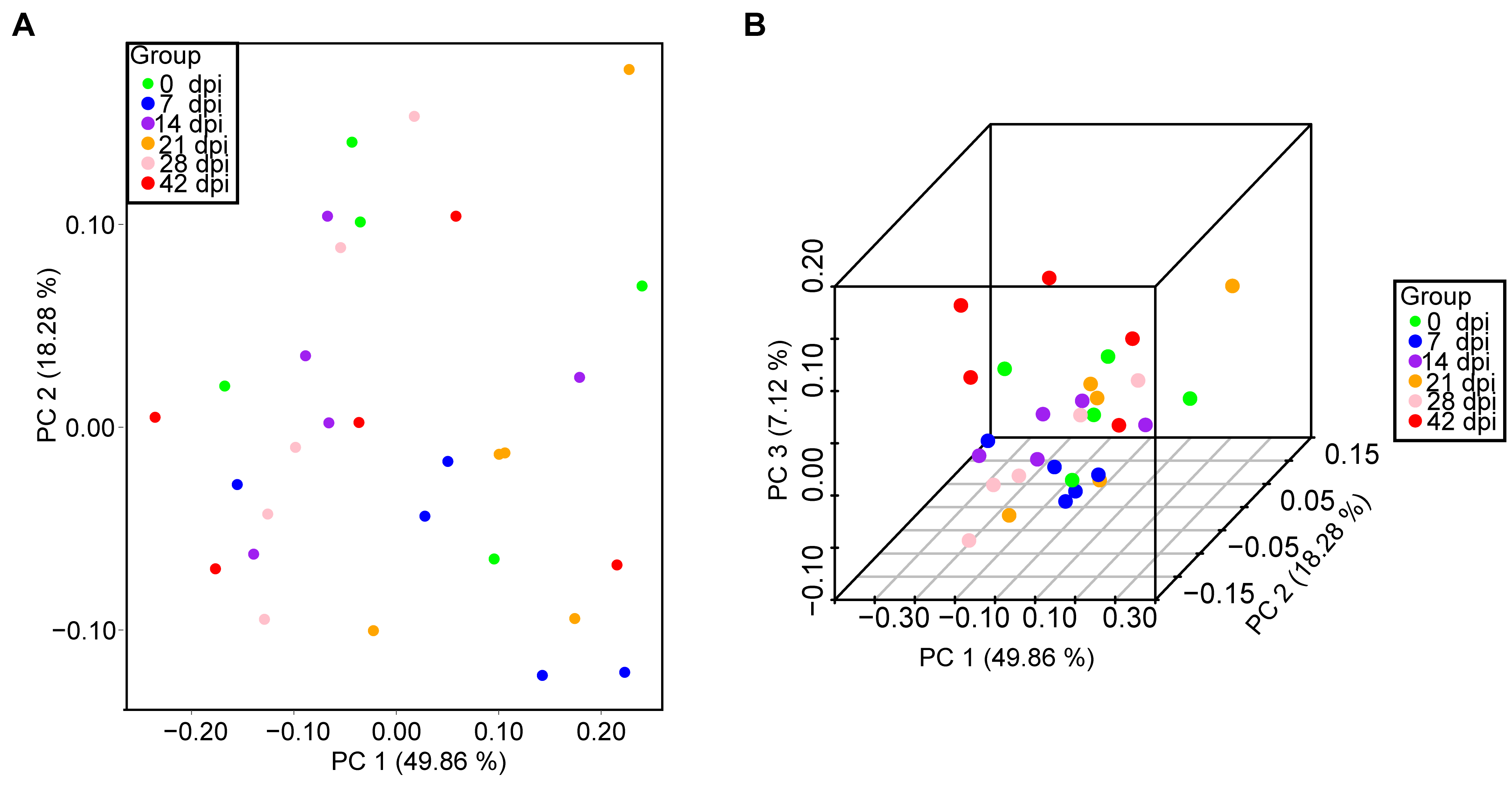

Supplement: Supplementary Figure 3 — Principal coordinate analysis (PCoA) plots showing beta diversity differences on the basis of 16S rRNA gene sequencing during S. japonicum infection. Figures were calculated using weighted UniFrac distance, and each point represents an individual. (A) Two-dimensional PCoA plot. (B) Three-dimensional PCoA plot. [file Image_3.TIF]

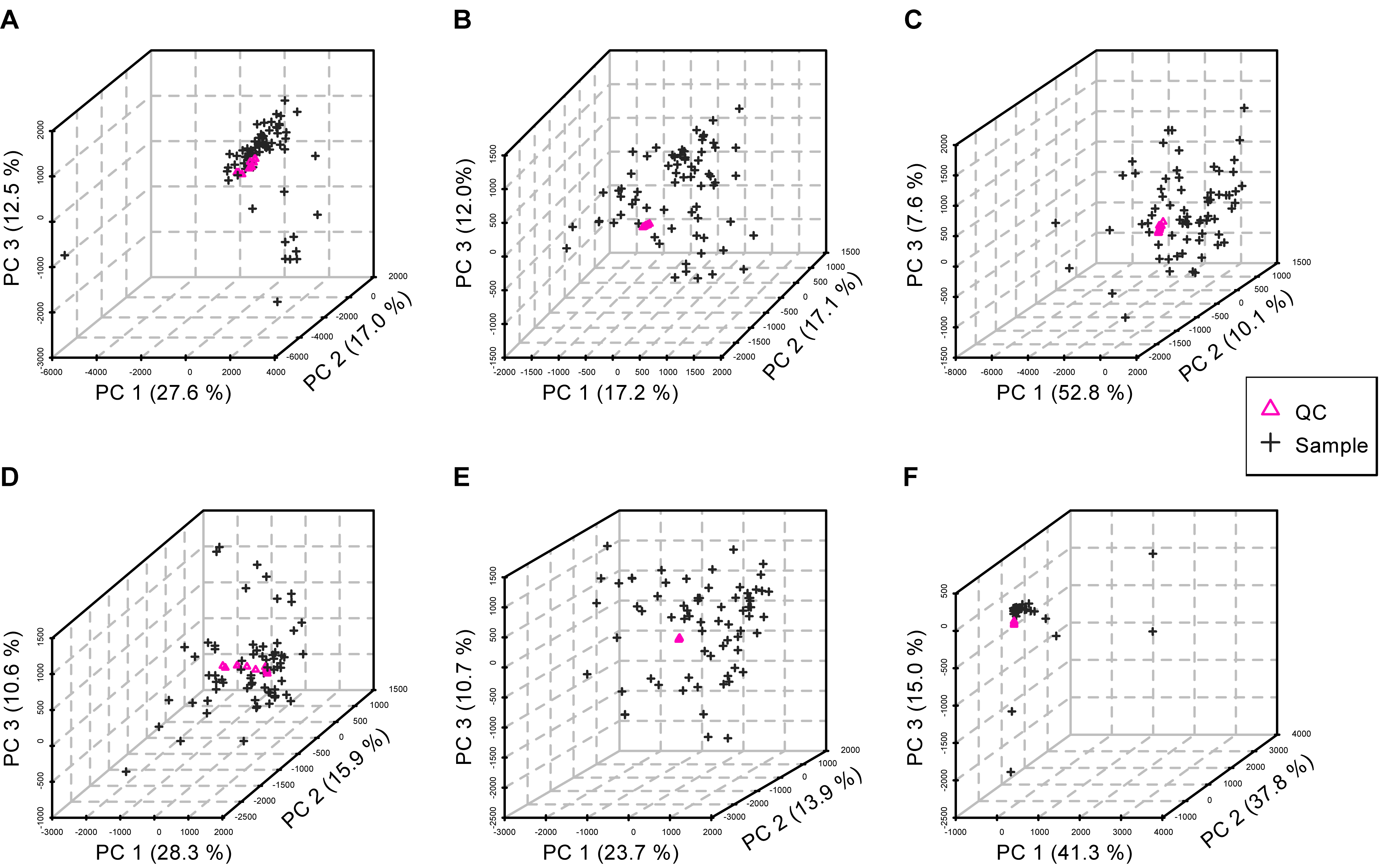

Supplement: Supplementary Figure 4 — Three-dimensional score plots of principal component analysis from quality control and samples. (A) Serum samples in positive electrospray ionization (ESI+) mode. (B) Urine samples in ESI+ mode. (C) Liver aqueous extracts in negative electrospray ionization (ESI-) mode. (D) Serum samples in ESI- mode. (E) Urine samples in ESI- mode. (F) Colon aqueous extracts in ESI- model. [file Image_4.TIF]

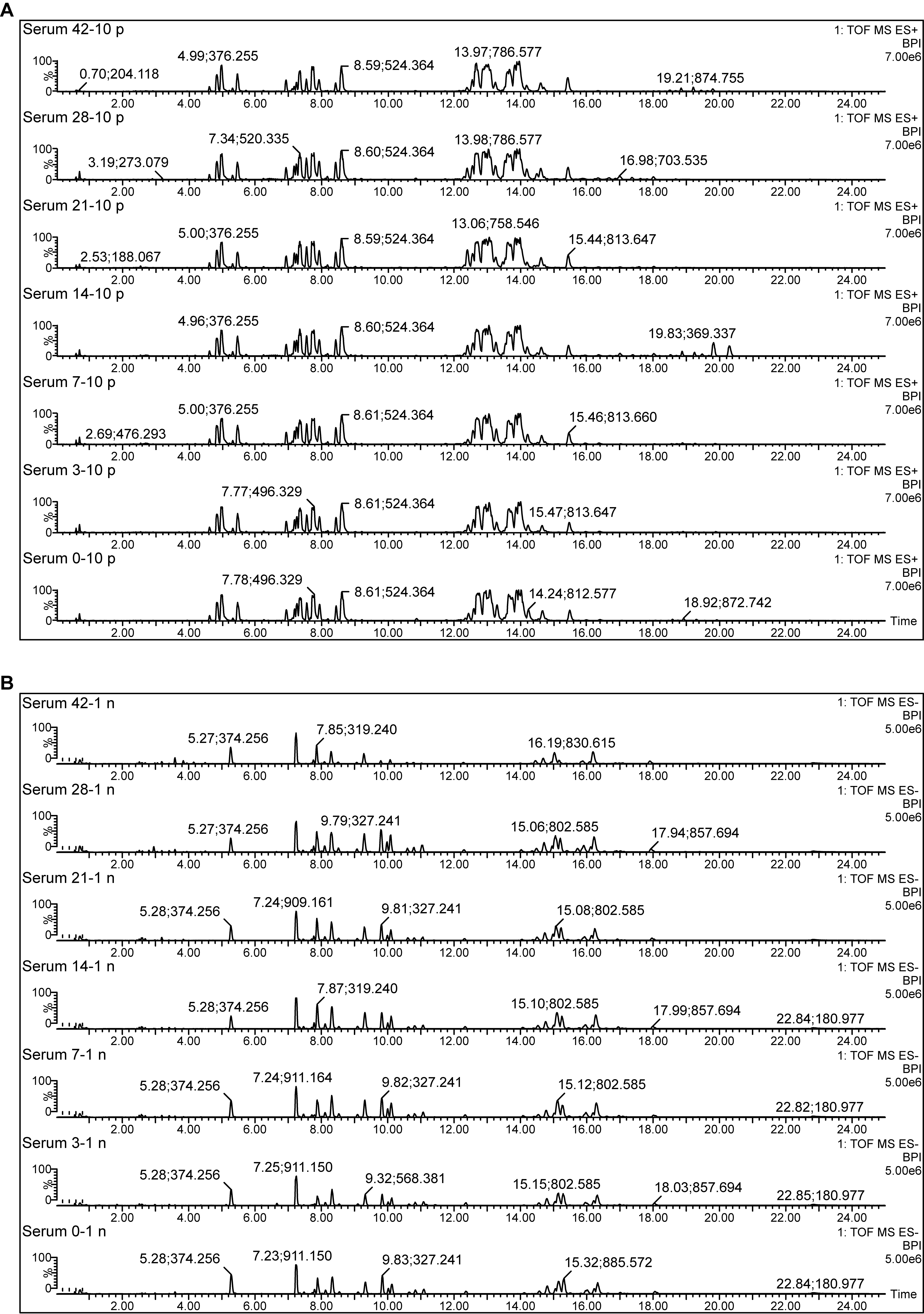

Supplement: Supplementary Figure 5 — Base peak intensity chromatograms of serum samples from different time points in ESI+ mode (A) and ESI- mode (B) as analyzed by quadrupole-time-of-flight mass spectrometry with a full scan. [file Image_5.TIF]

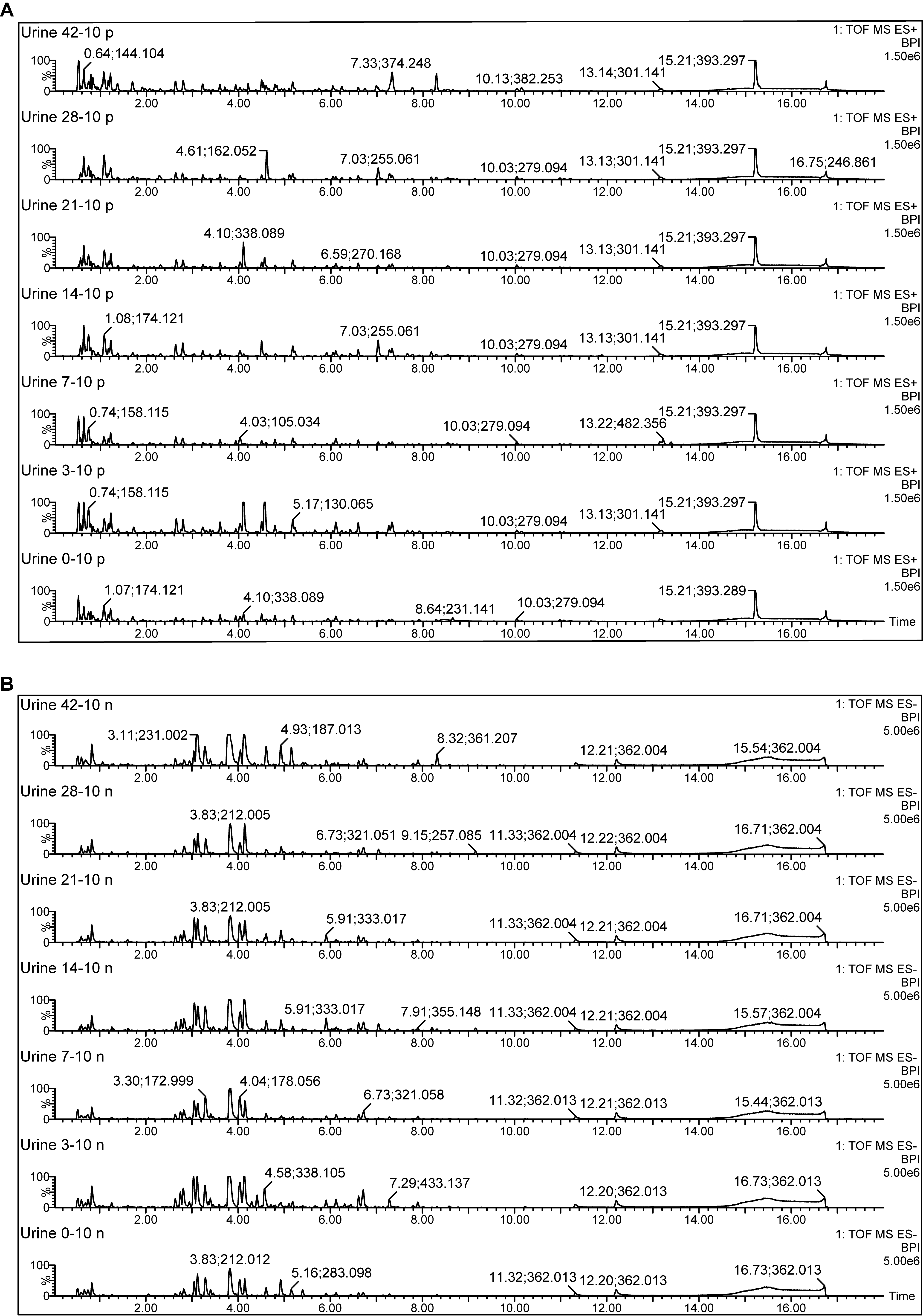

Supplement: Supplementary Figure 6 — Base peak intensity chromatograms of urine samples from different time points in ESI+ mode (A) and ESI- mode (B) as analyzed by quadrupole-time-of-flight mass spectrometry with a full scan. [file Image_6.TIF]

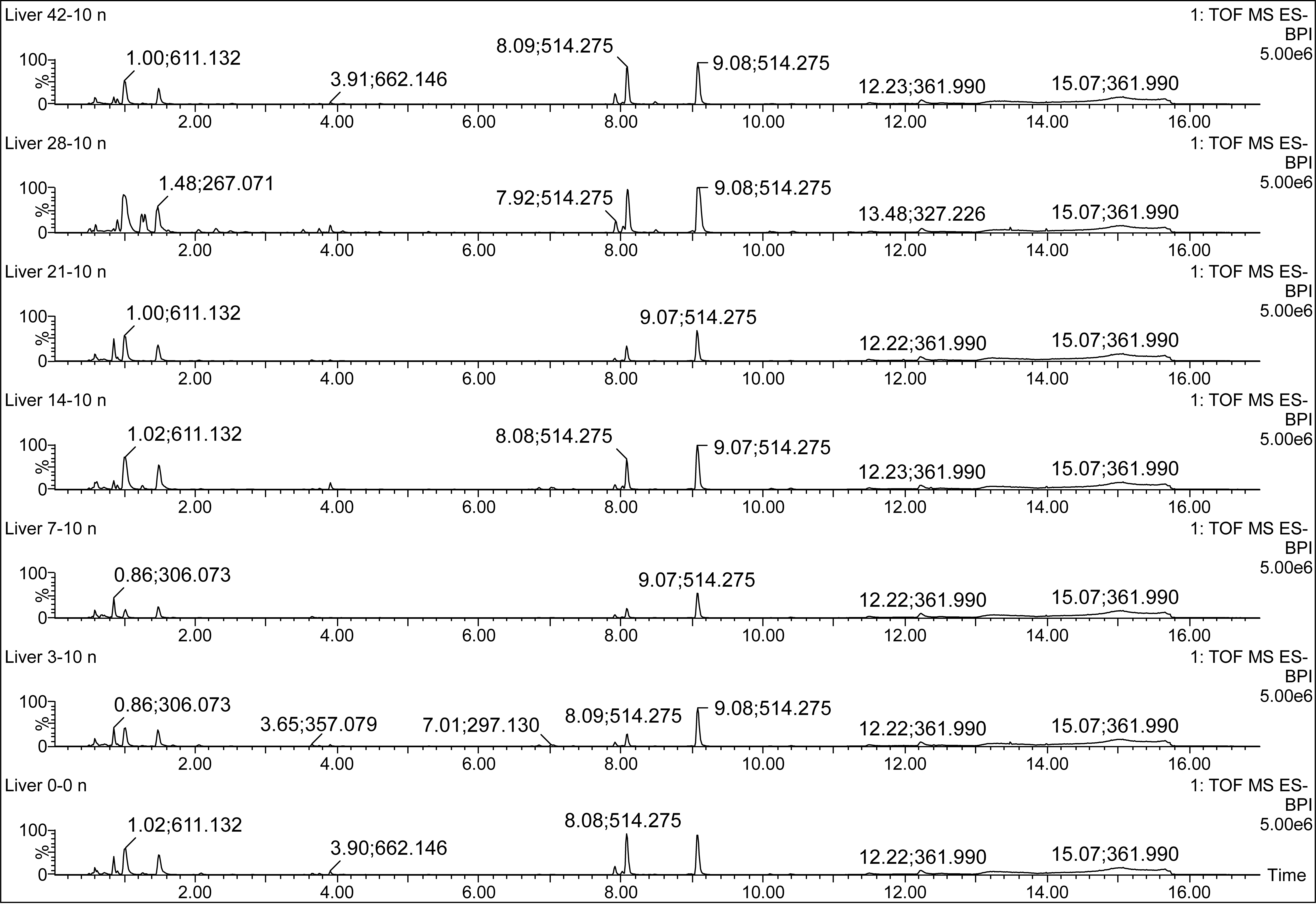

Supplement: Supplementary Figure 7 — Base peak intensity chromatograms of liver aqueous extracts from different time points in ESI- mode as analyzed by quadrupole-time-of-flight mass spectrometry with a full scan. [file Image_7.TIF]

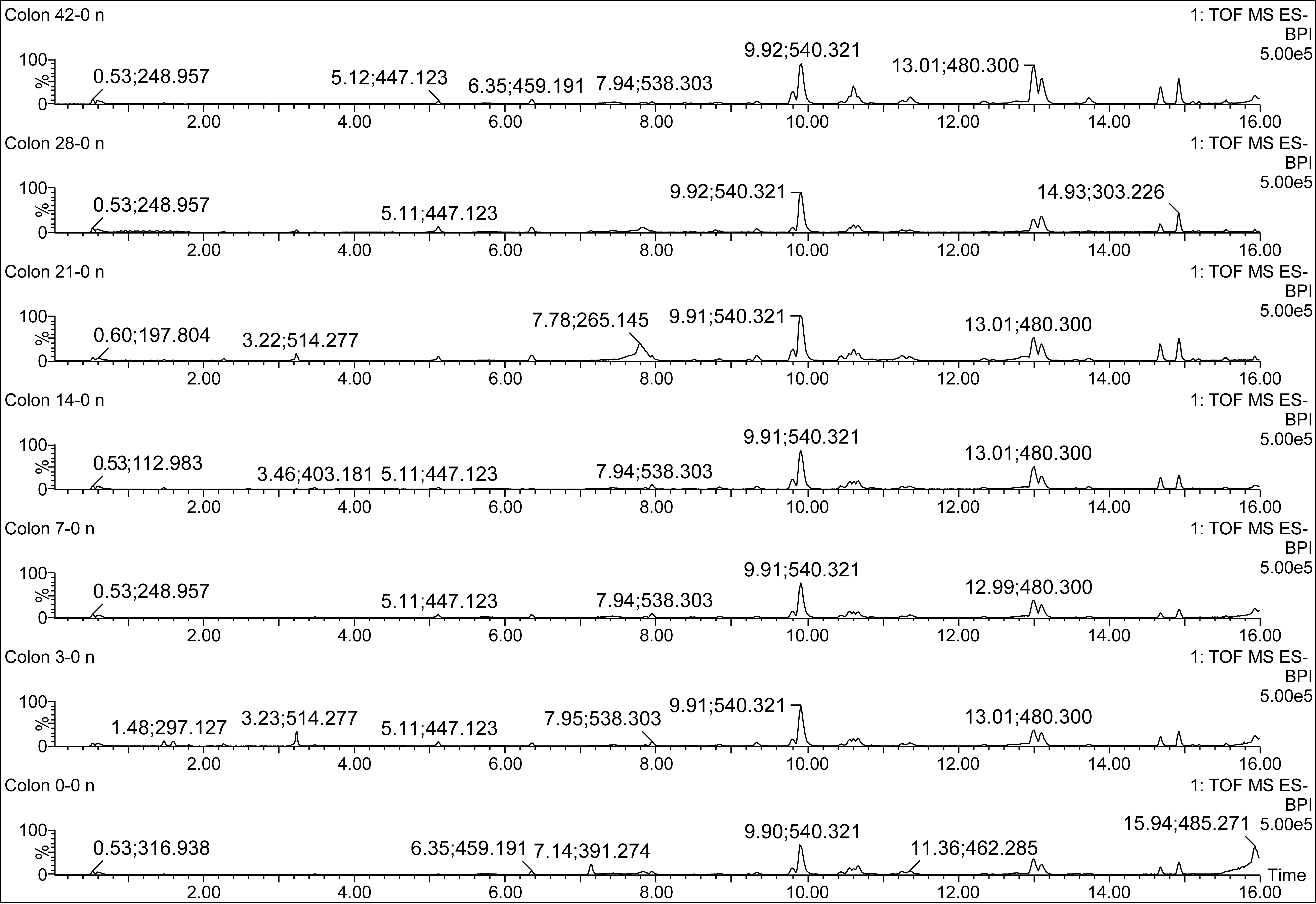

Supplement: Supplementary Figure 8 — Base peak intensity chromatograms of colon aqueous extracts from different time points in ESI- mode as analyzed by quadrupole-time-of-flight mass spectrometry with a full scan. [file Image_8.TIF]

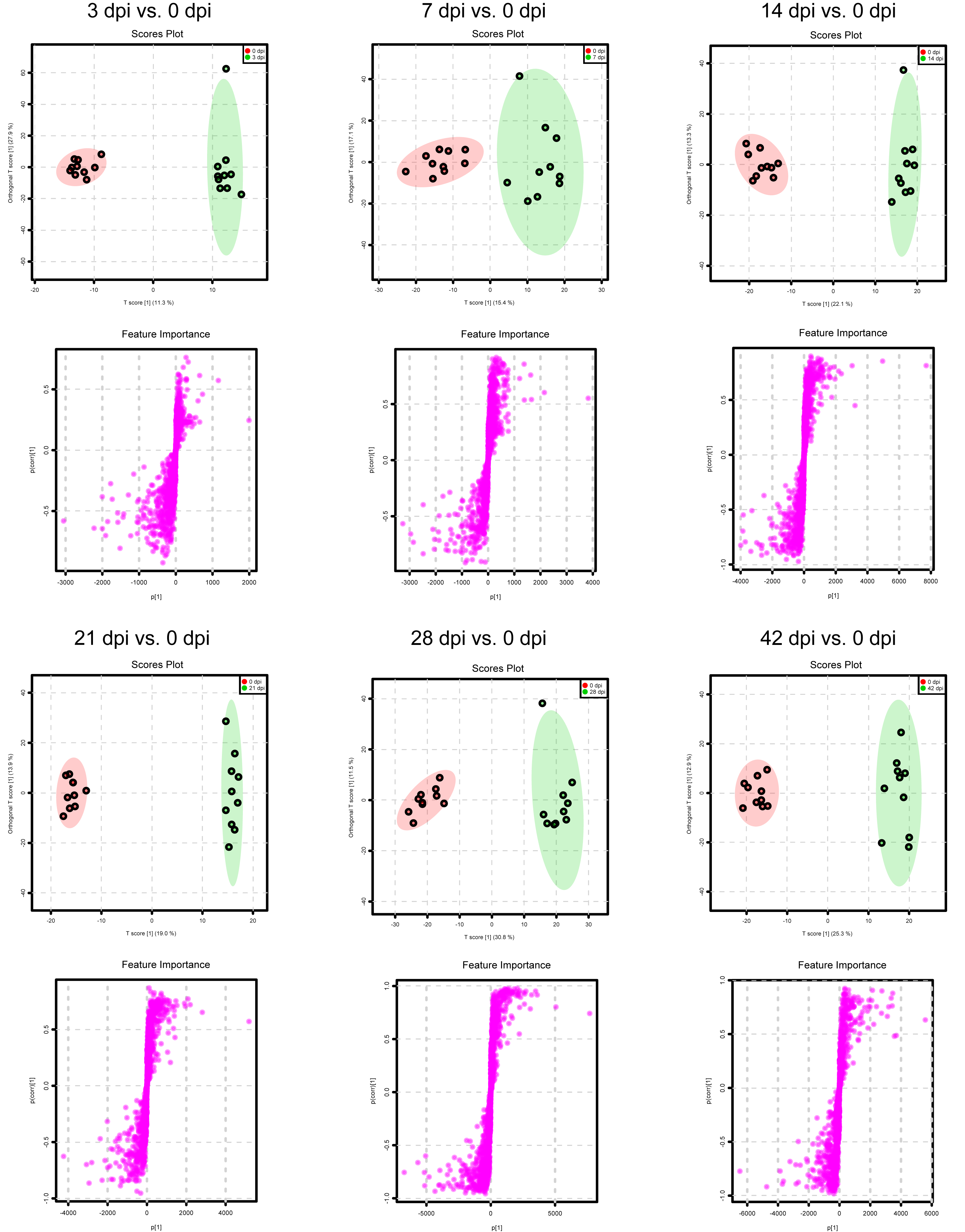

Supplement: Supplementary Figure 9 — Orthogonal partial least squares-discriminatory analysis (OPLS-DA) of serum extracts in ESI+ mode at different stages of Schistosoma japonicum infection. Each point represents one sample in score plots. OPLS-DA loadings S-plot combined with the covariance and the correlation loading profile indicate the variable importance. Each point represents a variable. [file Image_9.TIF]

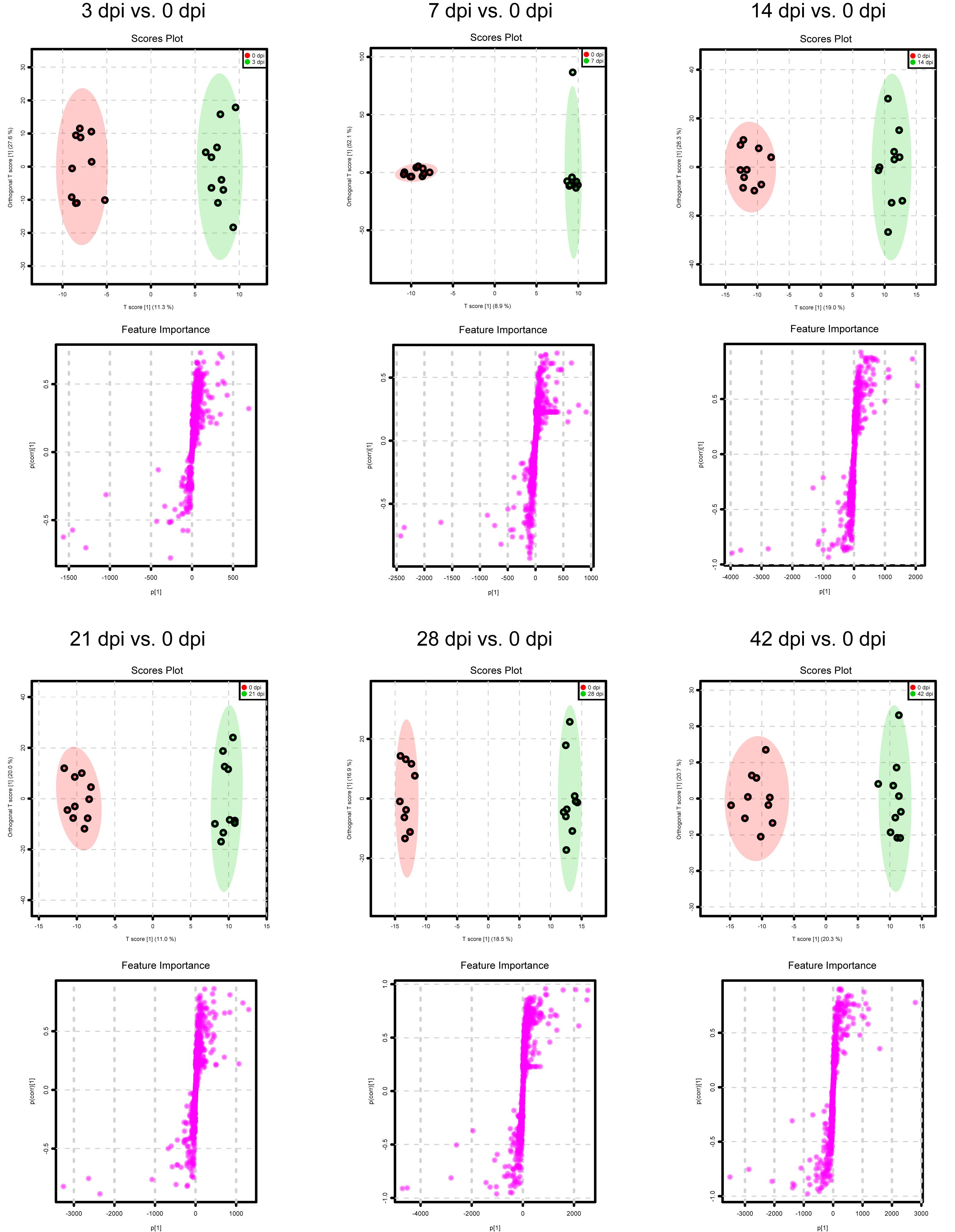

Supplement: Supplementary Figure 10 — Orthogonal partial least squares-discriminatory analysis (OPLS-DA) from serum extracts in ESI- mode at different stages of Schistosoma japonicum infection. Each point represents one sample in score plots. OPLS-DA loadings S-plot combined with the covariance and the correlation loading profile indicate the variable importance. Each point represents a variable. [file Image_10.TIF]

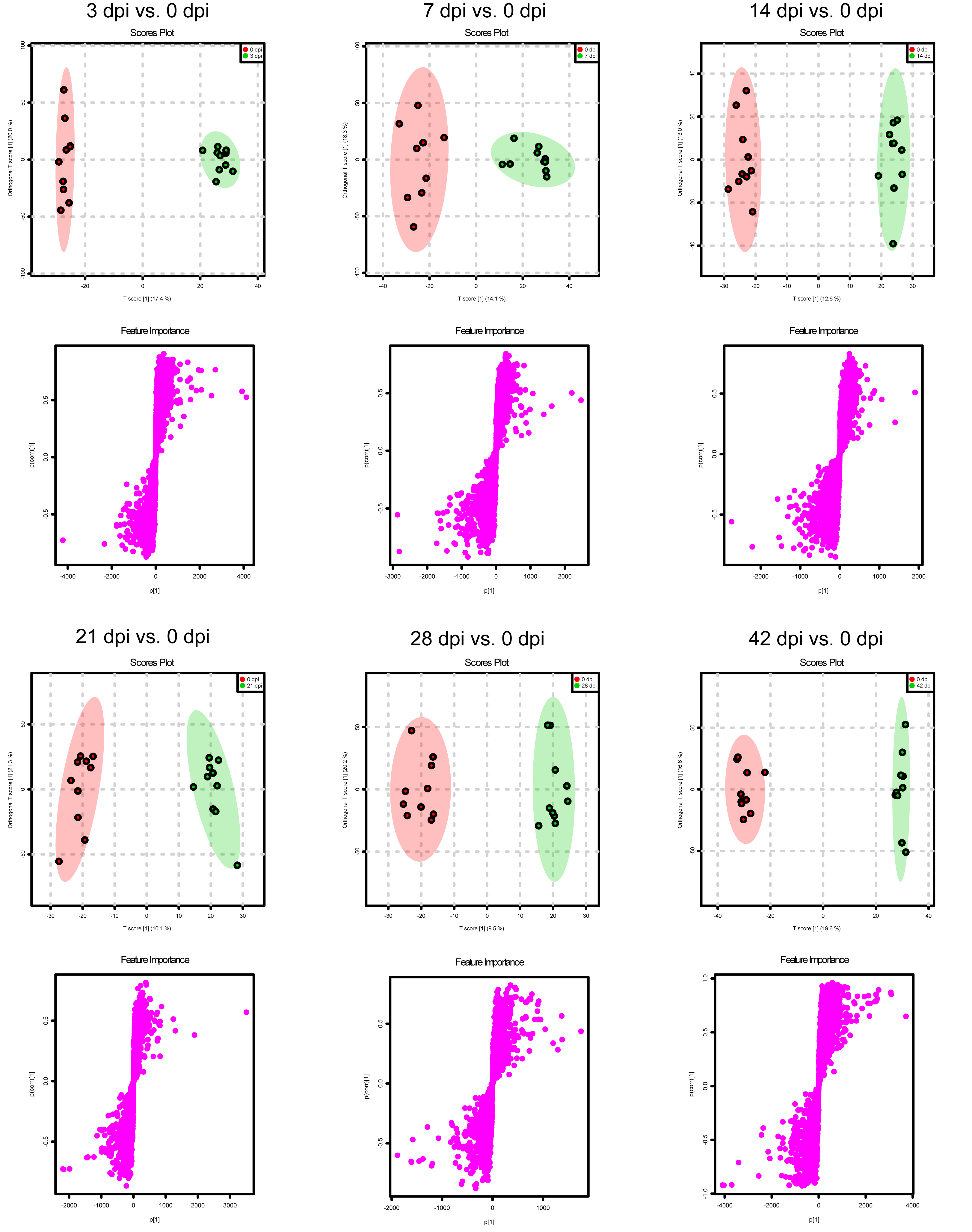

Supplement: Supplementary Figure 11 — Orthogonal partial least squares-discriminatory analysis (OPLS-DA) from urine extracts in ESI+ mode at different stages of Schistosoma japonicum infection. Each point represents one sample in score plots. OPLS-DA loadings S-plot combined with the covariance and the correlation loading profile indicate the variable importance. Each point represents a variable. [file Image_11.TIF]

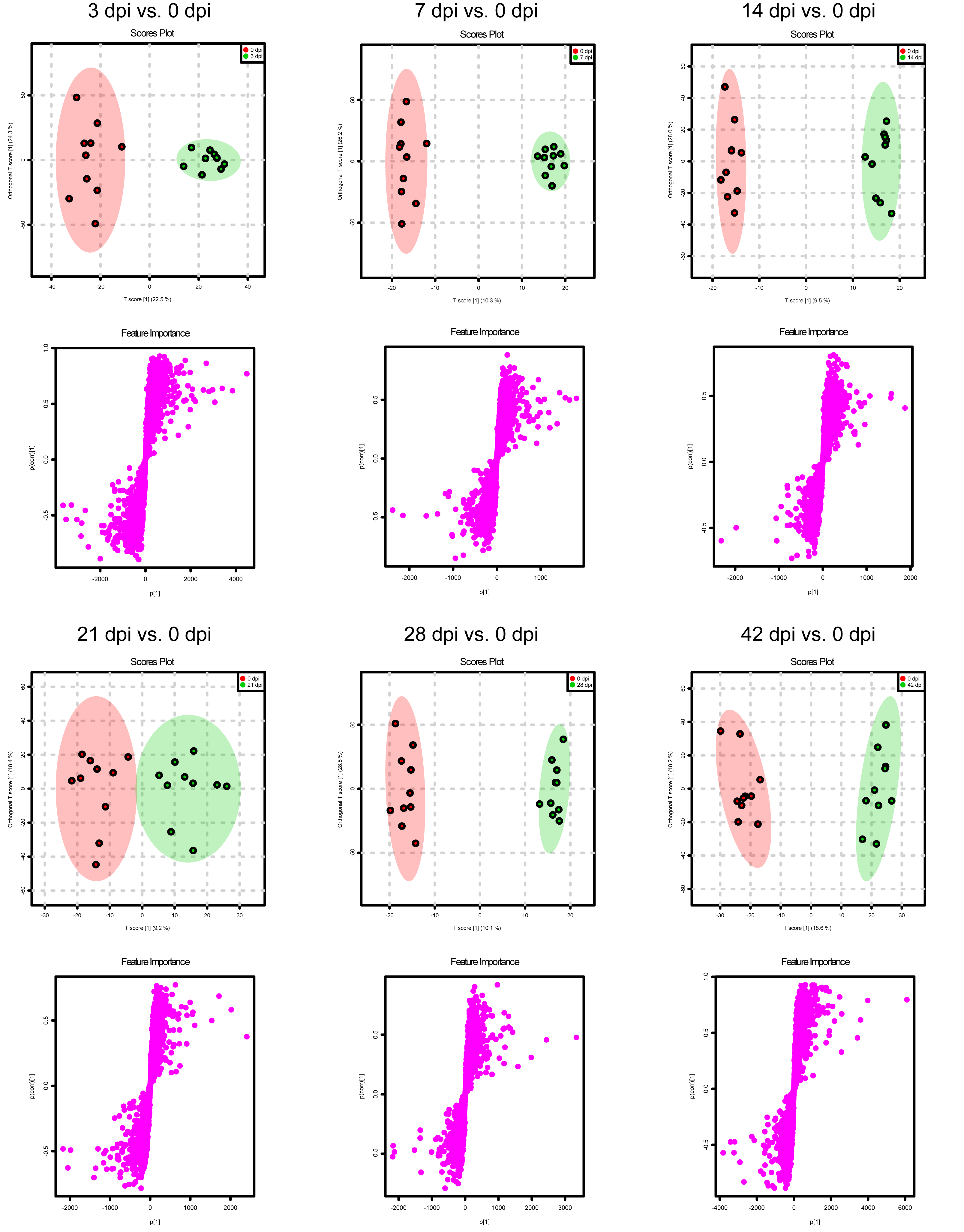

Supplement: Supplementary Figure 12 — Orthogonal partial least squares-discriminatory analysis (OPLS-DA) from urine extracts in ESI- mode at different stages of Schistosoma japonicum infection. Each point represents one sample in score plots. OPLS-DA loadings S-plot combined with the covariance and the correlation loading profile indicate the variable importance. Each point represents a variable. [file Image_12.TIF]

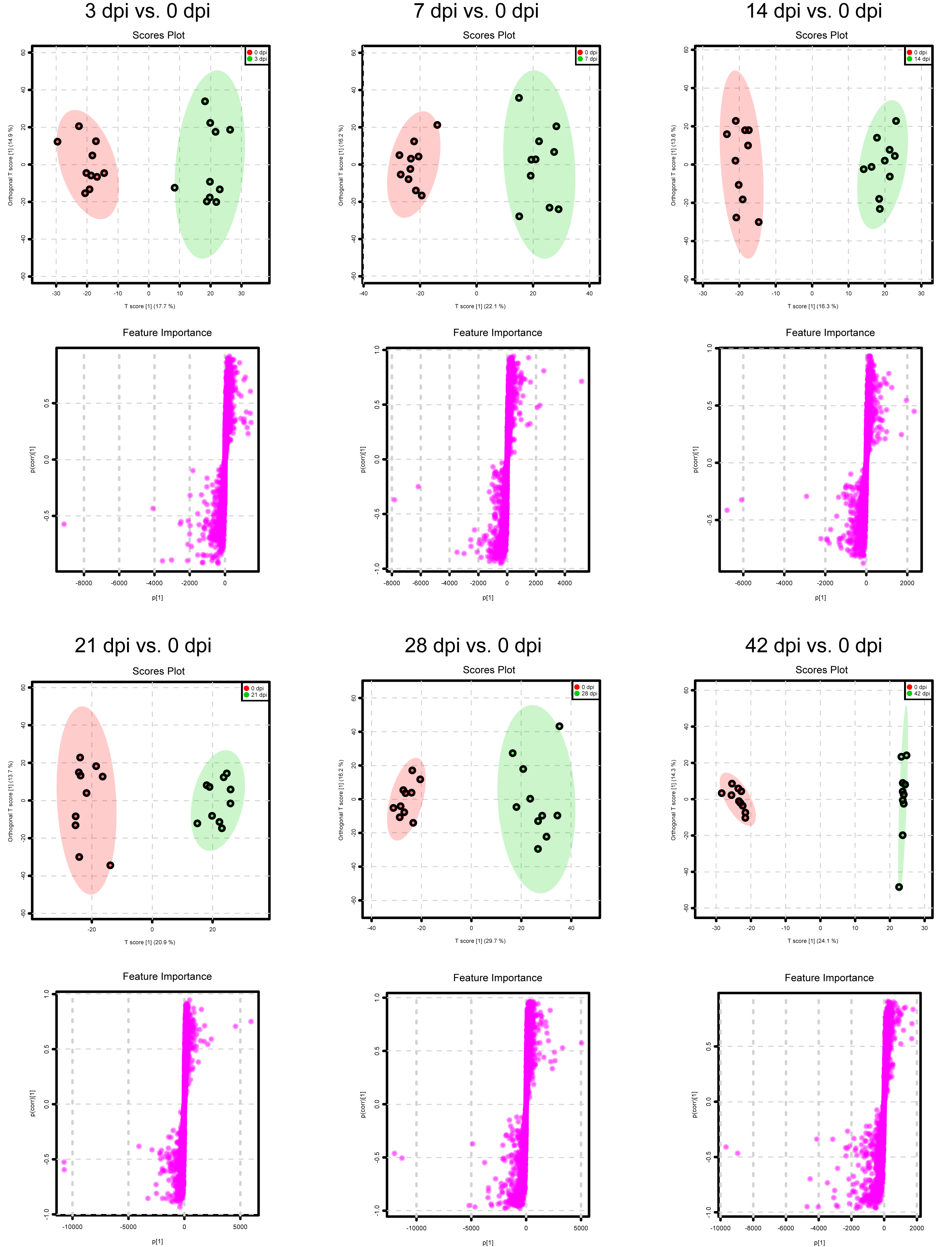

Supplement: Supplementary Figure 13 — Orthogonal partial least squares-discriminatory analysis (OPLS-DA) from liver aqueous extracts in ESI- mode at different stages of Schistosoma japonicum infection. Each point represents one sample in score plots. OPLS-DA loadings S-plot combined with the covariance and the correlation loading profile indicate the variable importance. Each point represents a variable. [file Image_13.TIF]

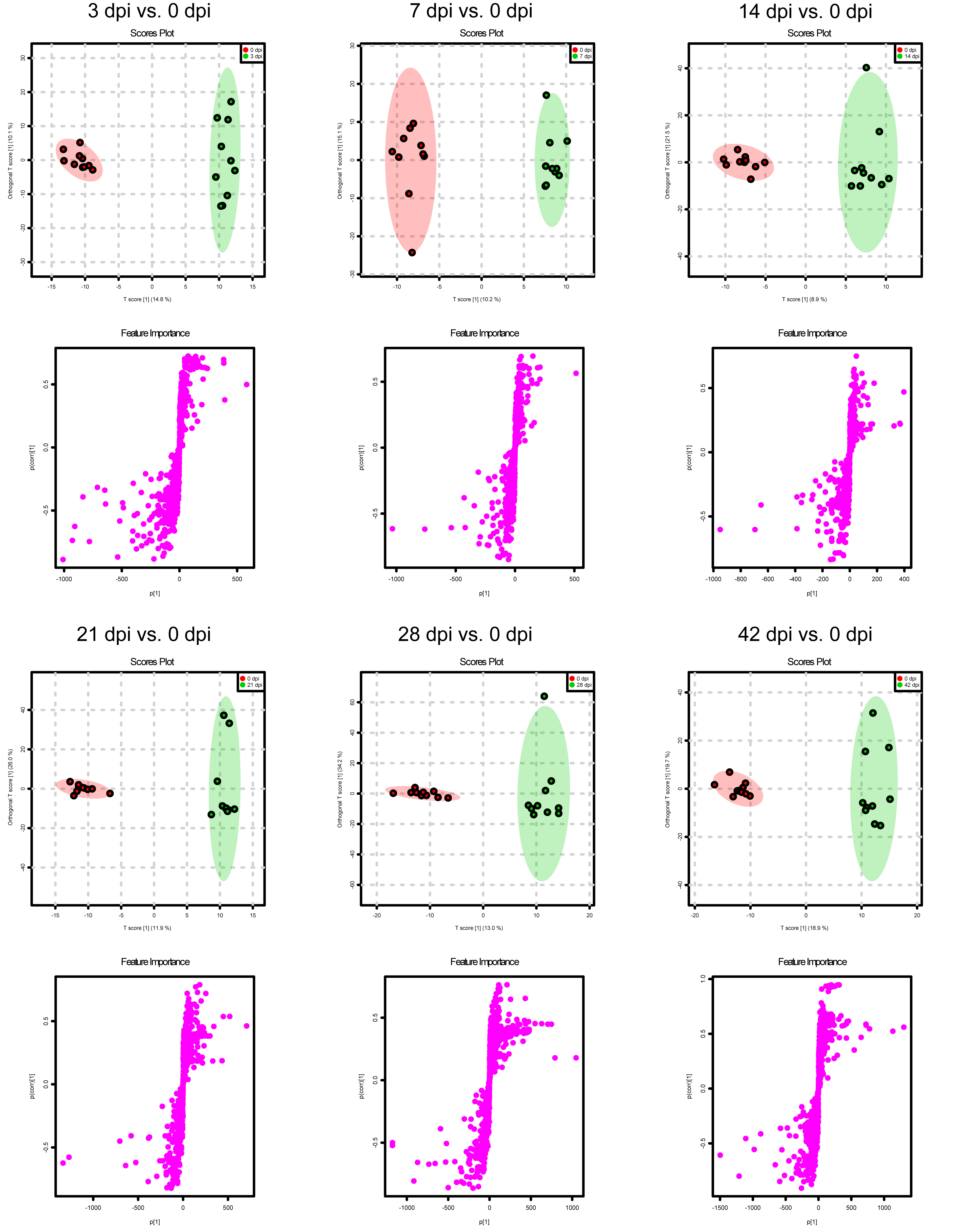

Supplement: Supplementary Figure 14 — Orthogonal partial least squares-discriminatory analysis (OPLS-DA) from colon aqueous extracts in ESI- mode at different stages of Schistosoma japonicum infection. Each point represents one sample in score plots. OPLS-DA loadings S-plot combined with the covariance and the correlation loading profile indicate the variable importance. Each point represents a variable. [file Image_14.TIF]

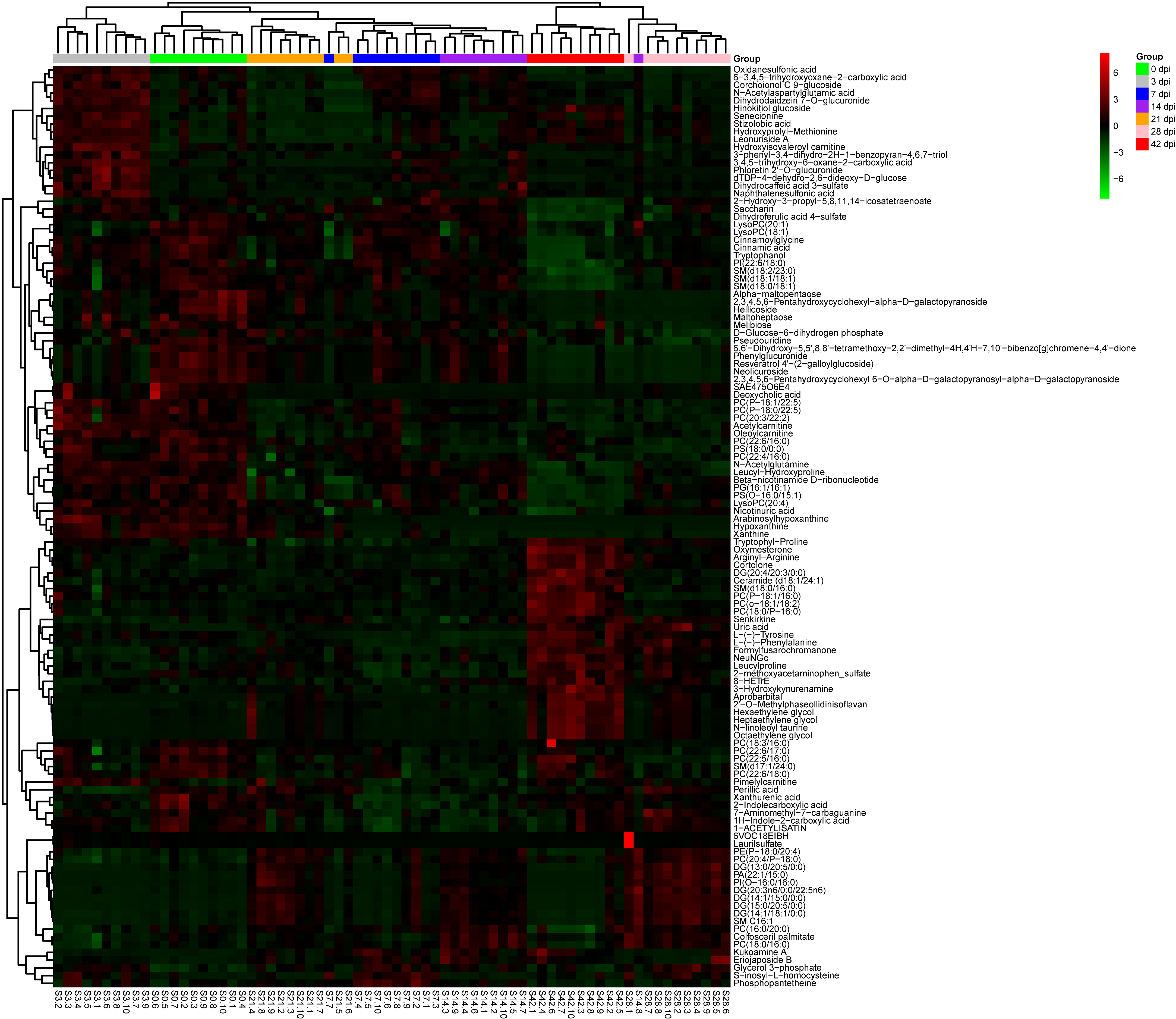

Supplement: Supplementary Figure 15 — Heat map visualization for the significantly different identified metabolites at different time points. Each column represents a sample, and each row represents a metabolite. The scaled intensity of the metabolites is reflected by the color key; higher intensity levels, when compared to the mean metabolite intensity value, are colored red, while lower intensity levels are colored green. Branch height represents the similarity between two metabolites, and metabolites with the shortest vertical difference indicate the closest connection. [file Image_15.TIF]
