# Supplementary material for: Alterations of Gut Microbiome and Metabolite Profiling in Mice Infected by Schistosoma japonicum
Source: Front Immunol. 2020 Oct 8;11:569727. doi: 10.3389/fimmu.2020.569727 (PMC7580221; doi:10.3389/fimmu.2020.569727)
Supplement: Supplementary Table 2 — One-way ANOVA of relative abundances of gut microbiome constituents at the phylum level. [file Table_2.DOCX]

| **Phylum** | ***p*-value** | **FDR** | **0 dpi** | **7 dpi** | **14 dpi** | **21 dpi** | **28 dpi** | **42 dpi** |
| --- | --- | --- | --- | --- | --- | --- | --- | --- |
| Actinobacteria | 0.00027 | 0.00337 | 0.0155 | 0.0211 | 0.0111 | 0.0102 | 0.0179 | 0.0072 |
| Saccharibacteria | 0.00031 | 0.00337 | 0.0000 | 0.0001 | 0.0000 | 0.0000 | 0.0000 | 0.0000 |
| Other | 0.01699 | 0.12458 | 0.0001 | 0.0001 | 0.0001 | 0.0002 | 0.0002 | 0.0010 |
| Proteobacteria | 0.06076 | 0.27577 | 0.0369 | 0.0221 | 0.0062 | 0.0582 | 0.0149 | 0.0747 |
| Deferribacteres | 0.06699 | 0.27577 | 0.0128 | 0.0033 | 0.0375 | 0.0151 | 0.0163 | 0.0098 |
| Gemmatimonadetes | 0.07521 | 0.27577 | 0.0000 | 0.0000 | 0.0000 | 0.0000 | 0.0000 | 0.0000 |
| Tenericutes | 0.13658 | 0.42924 | 0.0020 | 0.0004 | 0.0001 | 0.0001 | 0.0014 | 0.0068 |
| Spirochaetae | 0.18814 | 0.48280 | 0.0000 | 0.0000 | 0.0000 | 0.0000 | 0.0000 | 0.0000 |
| Firmicutes | 0.24489 | 0.48280 | 0.6166 | 0.6725 | 0.5465 | 0.7147 | 0.5139 | 0.4935 |
| Bacteroidetes | 0.26035 | 0.48280 | 0.3160 | 0.2804 | 0.3983 | 0.2014 | 0.4352 | 0.4066 |
| Fusobacteria | 0.28036 | 0.48280 | 0.0000 | 0.0000 | 0.0000 | 0.0000 | 0.0000 | 0.0001 |
| Cyanobacteria | 0.36447 | 0.48280 | 0.0000 | 0.0000 | 0.0000 | 0.0000 | 0.0000 | 0.0004 |
| Euryarchaeota | 0.37807 | 0.48280 | 0.0000 | 0.0000 | 0.0000 | 0.0000 | 0.0000 | 0.0000 |
| Acidobacteria | 0.42572 | 0.48280 | 0.0000 | 0.0000 | 0.0000 | 0.0000 | 0.0000 | 0.0000 |
| Aminicenantes | 0.43891 | 0.48280 | 0.0000 | 0.0000 | 0.0000 | 0.0000 | 0.0000 | 0.0000 |
| Chloroflexi | 0.43891 | 0.48280 | 0.0000 | 0.0000 | 0.0000 | 0.0000 | 0.0000 | 0.0000 |
| Fibrobacteres | 0.43891 | 0.48280 | 0.0000 | 0.0000 | 0.0000 | 0.0000 | 0.0000 | 0.0000 |
| Gracilibacteria | 0.43891 | 0.48280 | 0.0000 | 0.0000 | 0.0000 | 0.0000 | 0.0000 | 0.0000 |
| Latescibacteria | 0.43891 | 0.48280 | 0.0000 | 0.0000 | 0.0000 | 0.0000 | 0.0000 | 0.0000 |
| Verrucomicrobia | 0.43891 | 0.48280 | 0.0000 | 0.0000 | 0.0000 | 0.0000 | 0.0000 | 0.0000 |
| Chlorobi | 0.56050 | 0.58719 | 0.0000 | 0.0000 | 0.0000 | 0.0000 | 0.0000 | 0.0000 |
| Thaumarchaeota | 0.62743 | 0.62743 | 0.0000 | 0.0000 | 0.0000 | 0.0000 | 0.0000 | 0.0000 |
