# Supplementary material for: Alterations of Gut Microbiome and Metabolite Profiling in Mice Infected by Schistosoma japonicum
Source: Front Immunol. 2020 Oct 8;11:569727. doi: 10.3389/fimmu.2020.569727 (PMC7580221; doi:10.3389/fimmu.2020.569727)
Supplement: Supplementary Table 3 — One-way ANOVA of relative abundances of gut microbiome constituents at the genus level. [file Table_3.DOCX]

| **Genus** | ***p*-value** | **FDR** | **0 dpi** | **7 dpi** | **14 dpi** | **21 dpi** | **28 dpi** | **42 dpi** |
| --- | --- | --- | --- | --- | --- | --- | --- | --- |
| *Ruminococcaceae UCG-014* | 0.00008 | 0.02202 | 0.0112 | 0.0224 | 0.0097 | 0.0082 | 0.0064 | 0.0021 |
| *Parvibacter* | 0.00016 | 0.02202 | 0.0006 | 0.0001 | 0.0001 | 0.0001 | 0.0001 | 0.0001 |
| *Candidatus Saccharimonas* | 0.00031 | 0.02797 | 0.0000 | 0.0001 | 0.0000 | 0.0000 | 0.0000 | 0.0000 |
| *Enterorhabdus* | 0.00074 | 0.04289 | 0.0136 | 0.0196 | 0.0098 | 0.0096 | 0.0163 | 0.0068 |
| *Lachnospiraceae UCG-010* | 0.00078 | 0.04289 | 0.0000 | 0.0000 | 0.0000 | 0.0000 | 0.0000 | 0.0001 |
| *Rikenella* | 0.00129 | 0.05902 | 0.0000 | 0.0000 | 0.0000 | 0.0000 | 0.0000 | 0.0072 |
| *Family XIII UCG-001* | 0.00237 | 0.09268 | 0.0005 | 0.0004 | 0.0001 | 0.0001 | 0.0001 | 0.0001 |
| *[Eubacterium] ventriosum group* | 0.00283 | 0.09372 | 0.0004 | 0.0020 | 0.0007 | 0.0001 | 0.0005 | 0.0004 |
| *Brevundimonas* | 0.00308 | 0.09372 | 0.0000 | 0.0000 | 0.0000 | 0.0000 | 0.0001 | 0.0000 |
| *Ruminococcaceae UCG-013* | 0.00478 | 0.12485 | 0.0003 | 0.0004 | 0.0003 | 0.0010 | 0.0003 | 0.0001 |
| *Peptococcus* | 0.00501 | 0.12485 | 0.0003 | 0.0001 | 0.0001 | 0.0002 | 0.0002 | 0.0000 |
| *Desulfovibrio* | 0.01144 | 0.26127 | 0.0216 | 0.0064 | 0.0026 | 0.0003 | 0.0029 | 0.0097 |
| *Alistipes* | 0.01468 | 0.27920 | 0.0015 | 0.0275 | 0.0160 | 0.0129 | 0.0015 | 0.0199 |
| *[Eubacterium] brachy group* | 0.01525 | 0.27920 | 0.0004 | 0.0003 | 0.0002 | 0.0001 | 0.0002 | 0.0001 |
| *Odoribacter* | 0.01574 | 0.27920 | 0.0025 | 0.0046 | 0.0006 | 0.0015 | 0.0005 | 0.0223 |
| *Parabacteroides* | 0.01630 | 0.27920 | 0.0089 | 0.0018 | 0.0161 | 0.0093 | 0.0101 | 0.0477 |
| *Staphylococcus* | 0.01772 | 0.28553 | 0.0007 | 0.0112 | 0.0004 | 0.1446 | 0.0006 | 0.0024 |
| *Ambiguous taxa* | 0.02474 | 0.37663 | 0.0425 | 0.0400 | 0.0523 | 0.0433 | 0.0899 | 0.0209 |
| *Ruminiclostridium 5* | 0.02663 | 0.38110 | 0.0043 | 0.0027 | 0.0027 | 0.0019 | 0.0020 | 0.0011 |
| *Senegalimassilia* | 0.02782 | 0.38110 | 0.0000 | 0.0000 | 0.0000 | 0.0000 | 0.0000 | 0.0001 |
| *Coprococcus 1* | 0.03490 | 0.45536 | 0.0026 | 0.0010 | 0.0020 | 0.0021 | 0.0014 | 0.0047 |
| *Jeotgalicoccus* | 0.03671 | 0.45717 | 0.0000 | 0.0000 | 0.0000 | 0.0011 | 0.0000 | 0.0000 |
| *Ruminococcaceae NK4A214 group* | 0.04225 | 0.50329 | 0.0005 | 0.0002 | 0.0001 | 0.0002 | 0.0002 | 0.0001 |
| *Roseburia* | 0.04466 | 0.50989 | 0.0209 | 0.0067 | 0.0161 | 0.0069 | 0.0061 | 0.0039 |
| *Aliagarivorans* | 0.04828 | 0.51350 | 0.0000 | 0.0000 | 0.0000 | 0.0000 | 0.0000 | 0.0000 |
| *Candidatus Aquiluna* | 0.05018 | 0.51350 | 0.0000 | 0.0000 | 0.0000 | 0.0000 | 0.0000 | 0.0000 |
| *uncultured delta proteobacterium* | 0.05224 | 0.51350 | 0.0000 | 0.0000 | 0.0000 | 0.0000 | 0.0000 | 0.0000 |
| *Alloprevotella* | 0.05247 | 0.51350 | 0.0443 | 0.0060 | 0.0470 | 0.0039 | 0.0347 | 0.0201 |
| *Vibrio* | 0.06241 | 0.57616 | 0.0000 | 0.0000 | 0.0001 | 0.0000 | 0.0003 | 0.0018 |
| *Prevotellaceae UCG-001* | 0.06680 | 0.57616 | 0.0030 | 0.0004 | 0.0014 | 0.0000 | 0.0001 | 0.0049 |
| *Mucispirillum* | 0.06699 | 0.57616 | 0.0128 | 0.0033 | 0.0375 | 0.0151 | 0.0163 | 0.0098 |
| *Ruminococcaceae UCG-009* | 0.06729 | 0.57616 | 0.0019 | 0.0013 | 0.0011 | 0.0009 | 0.0010 | 0.0004 |
| *Solibacillus* | 0.07104 | 0.58982 | 0.0000 | 0.0003 | 0.0000 | 0.0001 | 0.0000 | 0.0000 |
| *Reyranella* | 0.07810 | 0.60235 | 0.0000 | 0.0000 | 0.0000 | 0.0000 | 0.0000 | 0.0000 |
| *Solobacterium* | 0.08337 | 0.60235 | 0.0000 | 0.0000 | 0.0000 | 0.0000 | 0.0000 | 0.0000 |
| *Lachnospiraceae FCS020 group* | 0.08712 | 0.60235 | 0.0023 | 0.0013 | 0.0019 | 0.0054 | 0.0040 | 0.0006 |
| *Ruminococcaceae UCG-010* | 0.08814 | 0.60235 | 0.0004 | 0.0003 | 0.0002 | 0.0001 | 0.0007 | 0.0006 |
| *Arcobacter* | 0.09038 | 0.60235 | 0.0000 | 0.0000 | 0.0000 | 0.0000 | 0.0002 | 0.0006 |
| *Thalassospira* | 0.09065 | 0.60235 | 0.0000 | 0.0000 | 0.0000 | 0.0000 | 0.0000 | 0.0073 |
| *Candidatus Stoquefichus* | 0.09313 | 0.60235 | 0.0016 | 0.0002 | 0.0005 | 0.0001 | 0.0002 | 0.0004 |
| *Fusobacterium* | 0.09705 | 0.60235 | 0.0000 | 0.0000 | 0.0000 | 0.0000 | 0.0000 | 0.0000 |
| *Actinobacillus* | 0.10352 | 0.60235 | 0.0000 | 0.0000 | 0.0000 | 0.0000 | 0.0000 | 0.0001 |
| *[Ruminococcus] gauvreauii group* | 0.10562 | 0.60235 | 0.0000 | 0.0000 | 0.0000 | 0.0000 | 0.0000 | 0.0000 |
| *Kocuria* | 0.10833 | 0.60235 | 0.0000 | 0.0000 | 0.0000 | 0.0001 | 0.0001 | 0.0000 |
| *Marinobacterium* | 0.10975 | 0.60235 | 0.0000 | 0.0000 | 0.0000 | 0.0000 | 0.0000 | 0.0000 |
| *Polaribacter* | 0.11558 | 0.60235 | 0.0000 | 0.0000 | 0.0000 | 0.0000 | 0.0000 | 0.0001 |
| *Lachnospiraceae UCG-005* | 0.11686 | 0.60235 | 0.0001 | 0.0001 | 0.0001 | 0.0001 | 0.0000 | 0.0000 |
| *Turicibacter* | 0.12183 | 0.60235 | 0.0000 | 0.0000 | 0.0000 | 0.0000 | 0.0000 | 0.0001 |
| *[Eubacterium] ruminantium group* | 0.12304 | 0.60235 | 0.0000 | 0.0000 | 0.0000 | 0.0000 | 0.0000 | 0.0000 |
| *Anaeroplasma* | 0.12318 | 0.60235 | 0.0015 | 0.0000 | 0.0001 | 0.0000 | 0.0014 | 0.0067 |
| *Acinetobacter* | 0.13593 | 0.60235 | 0.0000 | 0.0000 | 0.0000 | 0.0000 | 0.0000 | 0.0000 |
| *Rikenellaceae RC9 gut group* | 0.14365 | 0.60235 | 0.0000 | 0.0000 | 0.0002 | 0.0000 | 0.0000 | 0.0001 |
| *uncultured Bacteroidales bacterium* | 0.14831 | 0.60235 | 0.0639 | 0.0551 | 0.0706 | 0.0139 | 0.0669 | 0.0352 |
| *Marinifilum* | 0.15910 | 0.60235 | 0.0000 | 0.0000 | 0.0000 | 0.0000 | 0.0000 | 0.0000 |
| *Ruminococcaceae UCG-005* | 0.16087 | 0.60235 | 0.0004 | 0.0003 | 0.0001 | 0.0001 | 0.0004 | 0.0002 |
| *Dialister* | 0.16155 | 0.60235 | 0.0000 | 0.0000 | 0.0000 | 0.0000 | 0.0000 | 0.0000 |
| *hgcI clade* | 0.16427 | 0.60235 | 0.0000 | 0.0000 | 0.0001 | 0.0000 | 0.0001 | 0.0000 |
| *Candidatus Arthromitus* | 0.16428 | 0.60235 | 0.0000 | 0.0002 | 0.0000 | 0.0000 | 0.0000 | 0.0000 |
| *Aquibacter* | 0.17387 | 0.60235 | 0.0000 | 0.0000 | 0.0000 | 0.0000 | 0.0001 | 0.0000 |
| *Pseudoalteromonas* | 0.17657 | 0.60235 | 0.0000 | 0.0000 | 0.0000 | 0.0000 | 0.0000 | 0.0001 |
| *Sulfitobacter* | 0.17855 | 0.60235 | 0.0000 | 0.0000 | 0.0000 | 0.0000 | 0.0000 | 0.0000 |
| *Bacteroides* | 0.19227 | 0.60235 | 0.0489 | 0.0326 | 0.0668 | 0.0308 | 0.0400 | 0.1200 |
| *Tenacibaculum* | 0.19306 | 0.60235 | 0.0000 | 0.0000 | 0.0000 | 0.0000 | 0.0000 | 0.0000 |
| *Oceanospirillum* | 0.20224 | 0.60235 | 0.0000 | 0.0000 | 0.0000 | 0.0000 | 0.0000 | 0.0000 |
| *Lactococcus* | 0.21548 | 0.60235 | 0.0000 | 0.0002 | 0.0000 | 0.0253 | 0.0068 | 0.0001 |
| *[Eubacterium] nodatum group* | 0.23041 | 0.60235 | 0.0008 | 0.0011 | 0.0005 | 0.0003 | 0.0005 | 0.0011 |
| *Sphingomonas* | 0.23094 | 0.60235 | 0.0000 | 0.0000 | 0.0000 | 0.0000 | 0.0001 | 0.0000 |
| *Helicobacter* | 0.23523 | 0.60235 | 0.0009 | 0.0045 | 0.0014 | 0.0310 | 0.0093 | 0.0178 |
| *Ruminococcaceae UCG-002* | 0.23555 | 0.60235 | 0.0000 | 0.0000 | 0.0000 | 0.0000 | 0.0000 | 0.0000 |
| *Enterococcus* | 0.23672 | 0.60235 | 0.0043 | 0.0151 | 0.0003 | 0.0055 | 0.0001 | 0.0106 |
| *Corynebacterium* | 0.23791 | 0.60235 | 0.0001 | 0.0001 | 0.0001 | 0.0000 | 0.0000 | 0.0000 |
| *Ruminococcus 1* | 0.23798 | 0.60235 | 0.0006 | 0.0003 | 0.0007 | 0.0000 | 0.0036 | 0.0043 |
| *Ruminiclostridium* | 0.24036 | 0.60235 | 0.0019 | 0.0007 | 0.0025 | 0.0017 | 0.0012 | 0.0012 |
| *Alteromonas* | 0.24695 | 0.60235 | 0.0000 | 0.0000 | 0.0000 | 0.0000 | 0.0000 | 0.0000 |
| *Lachnospiraceae UCG-008* | 0.24725 | 0.60235 | 0.0006 | 0.0001 | 0.0005 | 0.0002 | 0.0002 | 0.0000 |
| *Erysipelatoclostridium* | 0.24763 | 0.60235 | 0.0016 | 0.0001 | 0.0039 | 0.0025 | 0.0006 | 0.0284 |
| *Pasteurella* | 0.25842 | 0.60235 | 0.0000 | 0.0000 | 0.0000 | 0.0000 | 0.0000 | 0.0001 |
| *Family XIII AD3011 group* | 0.29895 | 0.60235 | 0.0000 | 0.0001 | 0.0000 | 0.0000 | 0.0001 | 0.0007 |
| *Colwellia* | 0.30335 | 0.60235 | 0.0000 | 0.0000 | 0.0000 | 0.0000 | 0.0000 | 0.0000 |
| *Lysinibacillus* | 0.30682 | 0.60235 | 0.0000 | 0.0000 | 0.0000 | 0.0001 | 0.0000 | 0.0000 |
| *Psychrilyobacter* | 0.30697 | 0.60235 | 0.0000 | 0.0000 | 0.0000 | 0.0000 | 0.0000 | 0.0001 |
| *Bilophila* | 0.31013 | 0.60235 | 0.0000 | 0.0000 | 0.0000 | 0.0008 | 0.0006 | 0.0006 |
| *Lachnospiraceae UCG-004* | 0.31552 | 0.60235 | 0.0000 | 0.0000 | 0.0000 | 0.0000 | 0.0000 | 0.0000 |
| *Acetatifactor* | 0.32188 | 0.60235 | 0.0009 | 0.0004 | 0.0010 | 0.0000 | 0.0003 | 0.0001 |
| *NS3a marine group* | 0.32576 | 0.60235 | 0.0000 | 0.0000 | 0.0000 | 0.0000 | 0.0000 | 0.0000 |
| *Lactobacillus* | 0.32661 | 0.60235 | 0.0413 | 0.1262 | 0.0952 | 0.0444 | 0.1424 | 0.0758 |
| *Fusibacter* | 0.33389 | 0.60235 | 0.0000 | 0.0000 | 0.0000 | 0.0000 | 0.0000 | 0.0000 |
| *Anaerotruncus* | 0.34490 | 0.60235 | 0.0102 | 0.0047 | 0.0084 | 0.0075 | 0.0065 | 0.0165 |
| *OM27 clade* | 0.35932 | 0.60235 | 0.0000 | 0.0000 | 0.0000 | 0.0000 | 0.0000 | 0.0000 |
| *Prevotella 2* | 0.36724 | 0.60235 | 0.0000 | 0.0000 | 0.0002 | 0.0000 | 0.0000 | 0.0003 |
| *Clostridium sensu stricto 1* | 0.36884 | 0.60235 | 0.0000 | 0.0000 | 0.0002 | 0.0003 | 0.0000 | 0.0001 |
| *Ruminococcaceae UCG-004* | 0.37206 | 0.60235 | 0.0005 | 0.0001 | 0.0001 | 0.0006 | 0.0003 | 0.0002 |
| *uncultured archaeon* | 0.37807 | 0.60235 | 0.0000 | 0.0000 | 0.0000 | 0.0000 | 0.0000 | 0.0000 |
| *Ruminiclostridium 9* | 0.38744 | 0.60235 | 0.0065 | 0.0032 | 0.0051 | 0.0056 | 0.0044 | 0.0038 |
| *Other* | 0.39619 | 0.60235 | 0.3071 | 0.3940 | 0.2599 | 0.2914 | 0.2263 | 0.2327 |
| *Prevotella 9* | 0.39699 | 0.60235 | 0.0002 | 0.0001 | 0.0020 | 0.0002 | 0.0001 | 0.0006 |
| *CL500-29 marine group* | 0.40625 | 0.60235 | 0.0000 | 0.0000 | 0.0000 | 0.0000 | 0.0000 | 0.0000 |
| *Prolixibacter* | 0.41529 | 0.60235 | 0.0000 | 0.0000 | 0.0000 | 0.0000 | 0.0000 | 0.0001 |
| *Shewanella* | 0.42070 | 0.60235 | 0.0000 | 0.0000 | 0.0000 | 0.0000 | 0.0000 | 0.0000 |
| *Prevotella 1* | 0.42234 | 0.60235 | 0.0000 | 0.0000 | 0.0005 | 0.0001 | 0.0001 | 0.0005 |
| *[Eubacterium] coprostanoligenes group* | 0.42617 | 0.60235 | 0.0006 | 0.0005 | 0.0006 | 0.0000 | 0.0000 | 0.0000 |
| *Oceanobacillus* | 0.43391 | 0.60235 | 0.0000 | 0.0000 | 0.0000 | 0.0007 | 0.0000 | 0.0000 |
| *Acetitomaculum* | 0.43891 | 0.60235 | 0.0000 | 0.0000 | 0.0002 | 0.0000 | 0.0000 | 0.0000 |
| *Actinopolyspora* | 0.43891 | 0.60235 | 0.0000 | 0.0000 | 0.0000 | 0.0000 | 0.0000 | 0.0000 |
| *Alkanindiges* | 0.43891 | 0.60235 | 0.0000 | 0.0000 | 0.0000 | 0.0000 | 0.0000 | 0.0000 |
| *Alsobacter* | 0.43891 | 0.60235 | 0.0000 | 0.0000 | 0.0000 | 0.0000 | 0.0000 | 0.0000 |
| *Amphritea* | 0.43891 | 0.60235 | 0.0000 | 0.0000 | 0.0000 | 0.0000 | 0.0000 | 0.0000 |
| *Anaerostipes* | 0.43891 | 0.60235 | 0.0000 | 0.0000 | 0.0000 | 0.0000 | 0.0000 | 0.0000 |
| *Aquiflexum* | 0.43891 | 0.60235 | 0.0000 | 0.0000 | 0.0000 | 0.0000 | 0.0000 | 0.0000 |
| *Arthrobacter* | 0.43891 | 0.60235 | 0.0000 | 0.0000 | 0.0000 | 0.0000 | 0.0000 | 0.0000 |
| *Bosea* | 0.43891 | 0.60235 | 0.0000 | 0.0000 | 0.0000 | 0.0000 | 0.0000 | 0.0000 |
| *Bryobacter* | 0.43891 | 0.60235 | 0.0000 | 0.0000 | 0.0000 | 0.0000 | 0.0000 | 0.0000 |
| *Butyrivibrio* | 0.43891 | 0.60235 | 0.0000 | 0.0000 | 0.0000 | 0.0000 | 0.0000 | 0.0000 |
| *Campylobacter* | 0.43891 | 0.60235 | 0.0000 | 0.0000 | 0.0000 | 0.0000 | 0.0000 | 0.0000 |
| *Candidatus Endoecteinascidia* | 0.43891 | 0.60235 | 0.0000 | 0.0000 | 0.0000 | 0.0000 | 0.0000 | 0.0000 |
| *Candidatus Nitrososphaera* | 0.43891 | 0.60235 | 0.0000 | 0.0000 | 0.0000 | 0.0000 | 0.0000 | 0.0000 |
| *Candidatus Thiobios* | 0.43891 | 0.60235 | 0.0000 | 0.0000 | 0.0000 | 0.0000 | 0.0000 | 0.0000 |
| *Carnobacterium* | 0.43891 | 0.60235 | 0.0000 | 0.0000 | 0.0000 | 0.0000 | 0.0000 | 0.0000 |
| *Catenococcus* | 0.43891 | 0.60235 | 0.0000 | 0.0000 | 0.0000 | 0.0000 | 0.0000 | 0.0000 |
| *Cesiribacter* | 0.43891 | 0.60235 | 0.0000 | 0.0000 | 0.0000 | 0.0000 | 0.0000 | 0.0000 |
| *Cetobacterium* | 0.43891 | 0.60235 | 0.0000 | 0.0000 | 0.0000 | 0.0000 | 0.0000 | 0.0000 |
| *Christensenellaceae R-7 group* | 0.43891 | 0.60235 | 0.0000 | 0.0000 | 0.0000 | 0.0000 | 0.0000 | 0.0000 |
| *Clostridium sensu stricto 15* | 0.43891 | 0.60235 | 0.0000 | 0.0000 | 0.0000 | 0.0000 | 0.0000 | 0.0000 |
| *Clostridium sensu stricto 6* | 0.43891 | 0.60235 | 0.0000 | 0.0000 | 0.0000 | 0.0000 | 0.0000 | 0.0000 |
| *Comamonas* | 0.43891 | 0.60235 | 0.0000 | 0.0000 | 0.0000 | 0.0000 | 0.0000 | 0.0000 |
| *Coprococcus 2* | 0.43891 | 0.60235 | 0.0000 | 0.0000 | 0.0000 | 0.0000 | 0.0000 | 0.0000 |
| *Crocinitomix* | 0.43891 | 0.60235 | 0.0000 | 0.0000 | 0.0000 | 0.0000 | 0.0000 | 0.0000 |
| *Cronobacter* | 0.43891 | 0.60235 | 0.0000 | 0.0000 | 0.0000 | 0.0000 | 0.0000 | 0.0000 |
| *Desulfobacula* | 0.43891 | 0.60235 | 0.0000 | 0.0000 | 0.0000 | 0.0000 | 0.0000 | 0.0000 |
| *Desulfofaba* | 0.43891 | 0.60235 | 0.0000 | 0.0000 | 0.0000 | 0.0000 | 0.0000 | 0.0000 |
| *Desulfosarcina* | 0.43891 | 0.60235 | 0.0000 | 0.0000 | 0.0000 | 0.0000 | 0.0000 | 0.0000 |
| *Dorea* | 0.43891 | 0.60235 | 0.0000 | 0.0000 | 0.0001 | 0.0000 | 0.0000 | 0.0000 |
| *Facklamia* | 0.43891 | 0.60235 | 0.0000 | 0.0000 | 0.0000 | 0.0000 | 0.0000 | 0.0000 |
| *Family XIII UCG-002* | 0.43891 | 0.60235 | 0.0000 | 0.0001 | 0.0000 | 0.0000 | 0.0000 | 0.0000 |
| *Ferruginibacter* | 0.43891 | 0.60235 | 0.0000 | 0.0000 | 0.0000 | 0.0000 | 0.0000 | 0.0000 |
| *Fibrobacter* | 0.43891 | 0.60235 | 0.0000 | 0.0000 | 0.0000 | 0.0000 | 0.0000 | 0.0000 |
| *Flavobacterium* | 0.43891 | 0.60235 | 0.0000 | 0.0000 | 0.0000 | 0.0000 | 0.0000 | 0.0000 |
| *Formosa* | 0.43891 | 0.60235 | 0.0000 | 0.0000 | 0.0000 | 0.0000 | 0.0000 | 0.0000 |
| *Gemmatimonas* | 0.43891 | 0.60235 | 0.0000 | 0.0000 | 0.0000 | 0.0000 | 0.0000 | 0.0000 |
| *Glaciecola* | 0.43891 | 0.60235 | 0.0000 | 0.0000 | 0.0000 | 0.0000 | 0.0000 | 0.0000 |
| *Gordonibacter* | 0.43891 | 0.60235 | 0.0000 | 0.0000 | 0.0000 | 0.0000 | 0.0000 | 0.0000 |
| *Granulosicoccus* | 0.43891 | 0.60235 | 0.0000 | 0.0000 | 0.0000 | 0.0000 | 0.0000 | 0.0000 |
| *Haliscomenobacter* | 0.43891 | 0.60235 | 0.0000 | 0.0000 | 0.0000 | 0.0000 | 0.0000 | 0.0000 |
| *Hoeflea* | 0.43891 | 0.60235 | 0.0000 | 0.0000 | 0.0000 | 0.0000 | 0.0000 | 0.0000 |
| *Hyphomonas* | 0.43891 | 0.60235 | 0.0000 | 0.0000 | 0.0000 | 0.0000 | 0.0000 | 0.0000 |
| *Kangiella* | 0.43891 | 0.60235 | 0.0000 | 0.0000 | 0.0000 | 0.0000 | 0.0000 | 0.0000 |
| *Kytococcus* | 0.43891 | 0.60235 | 0.0000 | 0.0000 | 0.0000 | 0.0000 | 0.0000 | 0.0000 |
| *Lachnospiraceae UCG-003* | 0.43891 | 0.60235 | 0.0000 | 0.0000 | 0.0000 | 0.0000 | 0.0000 | 0.0000 |
| *Leeia* | 0.43891 | 0.60235 | 0.0000 | 0.0000 | 0.0000 | 0.0000 | 0.0000 | 0.0000 |
| *Leucobacter* | 0.43891 | 0.60235 | 0.0000 | 0.0000 | 0.0000 | 0.0000 | 0.0000 | 0.0000 |
| *Lewinella* | 0.43891 | 0.60235 | 0.0000 | 0.0000 | 0.0000 | 0.0000 | 0.0000 | 0.0000 |
| *Limnobacter* | 0.43891 | 0.60235 | 0.0000 | 0.0000 | 0.0000 | 0.0000 | 0.0000 | 0.0000 |
| *Lysobacter* | 0.43891 | 0.60235 | 0.0000 | 0.0000 | 0.0000 | 0.0000 | 0.0000 | 0.0000 |
| *Maribacter* | 0.43891 | 0.60235 | 0.0000 | 0.0000 | 0.0000 | 0.0000 | 0.0000 | 0.0000 |
| *Marinomonas* | 0.43891 | 0.60235 | 0.0000 | 0.0000 | 0.0000 | 0.0000 | 0.0000 | 0.0000 |
| *Mitsuokella* | 0.43891 | 0.60235 | 0.0000 | 0.0000 | 0.0000 | 0.0000 | 0.0000 | 0.0000 |
| *Moraxella* | 0.43891 | 0.60235 | 0.0000 | 0.0000 | 0.0000 | 0.0000 | 0.0000 | 0.0000 |
| *Moryella* | 0.43891 | 0.60235 | 0.0000 | 0.0000 | 0.0000 | 0.0000 | 0.0000 | 0.0000 |
| *Muriicola* | 0.43891 | 0.60235 | 0.0000 | 0.0000 | 0.0000 | 0.0000 | 0.0000 | 0.0000 |
| *Mycobacterium* | 0.43891 | 0.60235 | 0.0000 | 0.0000 | 0.0000 | 0.0000 | 0.0000 | 0.0000 |
| *Neptunomonas* | 0.43891 | 0.60235 | 0.0000 | 0.0000 | 0.0000 | 0.0000 | 0.0000 | 0.0000 |
| *Nitriliruptor* | 0.43891 | 0.60235 | 0.0000 | 0.0000 | 0.0000 | 0.0000 | 0.0000 | 0.0000 |
| *Nordella* | 0.43891 | 0.60235 | 0.0000 | 0.0000 | 0.0000 | 0.0000 | 0.0000 | 0.0000 |
| *Novosphingobium* | 0.43891 | 0.60235 | 0.0000 | 0.0000 | 0.0000 | 0.0000 | 0.0000 | 0.0000 |
| *Owenweeksia* | 0.43891 | 0.60235 | 0.0000 | 0.0000 | 0.0000 | 0.0000 | 0.0000 | 0.0000 |
| *Paenibacillus* | 0.43891 | 0.60235 | 0.0000 | 0.0000 | 0.0000 | 0.0000 | 0.0000 | 0.0000 |
| *Pediococcus* | 0.43891 | 0.60235 | 0.0000 | 0.0000 | 0.0000 | 0.0000 | 0.0000 | 0.0000 |
| *Planomicrobium* | 0.43891 | 0.60235 | 0.0000 | 0.0000 | 0.0000 | 0.0000 | 0.0000 | 0.0000 |
| *Pleionea* | 0.43891 | 0.60235 | 0.0000 | 0.0000 | 0.0000 | 0.0000 | 0.0000 | 0.0000 |
| *Porphyromonas* | 0.43891 | 0.60235 | 0.0000 | 0.0000 | 0.0000 | 0.0000 | 0.0000 | 0.0000 |
| *Portibacter* | 0.43891 | 0.60235 | 0.0000 | 0.0000 | 0.0000 | 0.0000 | 0.0000 | 0.0000 |
| *Prevotellaceae UCG-003* | 0.43891 | 0.60235 | 0.0000 | 0.0000 | 0.0000 | 0.0000 | 0.0000 | 0.0000 |
| *Proteiniclasticum* | 0.43891 | 0.60235 | 0.0000 | 0.0000 | 0.0000 | 0.0000 | 0.0000 | 0.0000 |
| *Proteus* | 0.43891 | 0.60235 | 0.0000 | 0.0000 | 0.0000 | 0.0002 | 0.0000 | 0.0000 |
| *Pseudoruegeria* | 0.43891 | 0.60235 | 0.0000 | 0.0000 | 0.0000 | 0.0000 | 0.0000 | 0.0000 |
| *Pseudoxanthomonas* | 0.43891 | 0.60235 | 0.0000 | 0.0000 | 0.0000 | 0.0000 | 0.0000 | 0.0000 |
| *Psychroflexus* | 0.43891 | 0.60235 | 0.0000 | 0.0000 | 0.0000 | 0.0000 | 0.0000 | 0.0000 |
| *Rhizobium* | 0.43891 | 0.60235 | 0.0000 | 0.0000 | 0.0000 | 0.0000 | 0.0000 | 0.0000 |
| *Ruminococcaceae UCG-008* | 0.43891 | 0.60235 | 0.0000 | 0.0000 | 0.0000 | 0.0000 | 0.0000 | 0.0000 |
| *Saccharopolyspora* | 0.43891 | 0.60235 | 0.0000 | 0.0000 | 0.0000 | 0.0000 | 0.0000 | 0.0000 |
| *Shimia* | 0.43891 | 0.60235 | 0.0000 | 0.0000 | 0.0000 | 0.0000 | 0.0000 | 0.0000 |
| *Shuttleworthia* | 0.43891 | 0.60235 | 0.0000 | 0.0000 | 0.0000 | 0.0000 | 0.0000 | 0.0000 |
| *Sufflavibacter* | 0.43891 | 0.60235 | 0.0000 | 0.0000 | 0.0000 | 0.0000 | 0.0000 | 0.0000 |
| *Sva0081 sediment group* | 0.43891 | 0.60235 | 0.0000 | 0.0000 | 0.0000 | 0.0000 | 0.0000 | 0.0000 |
| *Terrisporobacter* | 0.43891 | 0.60235 | 0.0000 | 0.0000 | 0.0000 | 0.0000 | 0.0000 | 0.0000 |
| *Thalassotalea* | 0.43891 | 0.60235 | 0.0000 | 0.0000 | 0.0000 | 0.0000 | 0.0000 | 0.0000 |
| *Treponema 2* | 0.43891 | 0.60235 | 0.0000 | 0.0000 | 0.0000 | 0.0000 | 0.0000 | 0.0000 |
| *Ulvibacter* | 0.43891 | 0.60235 | 0.0000 | 0.0000 | 0.0000 | 0.0000 | 0.0000 | 0.0000 |
| *Weissella* | 0.43891 | 0.60235 | 0.0000 | 0.0000 | 0.0000 | 0.0000 | 0.0000 | 0.0000 |
| *endosymbionts* | 0.43891 | 0.60235 | 0.0000 | 0.0000 | 0.0000 | 0.0000 | 0.0000 | 0.0000 |
| *uncultured Acidobacteria bacterium* | 0.43891 | 0.60235 | 0.0000 | 0.0000 | 0.0000 | 0.0000 | 0.0000 | 0.0000 |
| *uncultured Erysipelotrichi bacterium* | 0.43891 | 0.60235 | 0.0000 | 0.0000 | 0.0000 | 0.0000 | 0.0000 | 0.0000 |
| *uncultured Sphingobacterium sp.* | 0.43891 | 0.60235 | 0.0000 | 0.0000 | 0.0000 | 0.0000 | 0.0000 | 0.0000 |
| *Escherichia-Shigella* | 0.44535 | 0.60235 | 0.0138 | 0.0109 | 0.0011 | 0.0253 | 0.0007 | 0.0347 |
| *Anaerovorax* | 0.44540 | 0.60235 | 0.0001 | 0.0000 | 0.0001 | 0.0000 | 0.0001 | 0.0000 |
| *Lachnospiraceae NK4A136 group* | 0.44719 | 0.60235 | 0.1119 | 0.0622 | 0.0768 | 0.0859 | 0.0909 | 0.0424 |
| *Bhargavaea* | 0.45301 | 0.60235 | 0.0000 | 0.0000 | 0.0000 | 0.0001 | 0.0000 | 0.0000 |
| *Romboutsia* | 0.45464 | 0.60235 | 0.0000 | 0.0000 | 0.0000 | 0.0000 | 0.0009 | 0.0000 |
| *Pantoea* | 0.45885 | 0.60235 | 0.0000 | 0.0000 | 0.0000 | 0.0000 | 0.0000 | 0.0000 |
| *Bifidobacterium* | 0.45896 | 0.60235 | 0.0000 | 0.0000 | 0.0000 | 0.0000 | 0.0008 | 0.0001 |
| *Hydrogenoanaerobacterium* | 0.45980 | 0.60235 | 0.0000 | 0.0000 | 0.0000 | 0.0000 | 0.0000 | 0.0000 |
| *Lachnospiraceae UCG-006* | 0.47088 | 0.60235 | 0.0037 | 0.0050 | 0.0019 | 0.0049 | 0.0020 | 0.0009 |
| *Bacillus* | 0.47128 | 0.60235 | 0.0000 | 0.0001 | 0.0000 | 0.0006 | 0.0001 | 0.0001 |
| *Ornithinibacillus* | 0.47421 | 0.60235 | 0.0000 | 0.0000 | 0.0000 | 0.0001 | 0.0000 | 0.0000 |
| *Lachnoclostridium* | 0.47913 | 0.60235 | 0.0084 | 0.0083 | 0.0130 | 0.0120 | 0.0097 | 0.0047 |
| *Collinsella* | 0.48120 | 0.60235 | 0.0000 | 0.0000 | 0.0001 | 0.0000 | 0.0000 | 0.0000 |
| *Erysipelotrichaceae UCG-004* | 0.48151 | 0.60235 | 0.0000 | 0.0000 | 0.0001 | 0.0000 | 0.0000 | 0.0000 |
| *Gemella* | 0.48374 | 0.60235 | 0.0000 | 0.0000 | 0.0002 | 0.0003 | 0.0001 | 0.0002 |
| *MWH-UniP1 aquatic group* | 0.48608 | 0.60235 | 0.0000 | 0.0000 | 0.0000 | 0.0000 | 0.0000 | 0.0000 |
| *Aerococcus* | 0.48838 | 0.60235 | 0.0000 | 0.0002 | 0.0000 | 0.0006 | 0.0000 | 0.0001 |
| *Succinivibrio* | 0.49044 | 0.60235 | 0.0001 | 0.0000 | 0.0002 | 0.0000 | 0.0001 | 0.0004 |
| *Psychrosphaera* | 0.49144 | 0.60235 | 0.0000 | 0.0000 | 0.0000 | 0.0000 | 0.0000 | 0.0000 |
| *Terribacillus* | 0.49373 | 0.60235 | 0.0000 | 0.0000 | 0.0000 | 0.0001 | 0.0000 | 0.0000 |
| *Allobaculum* | 0.49673 | 0.60235 | 0.0000 | 0.0000 | 0.0000 | 0.0000 | 0.0000 | 0.0000 |
| *Exiguobacterium* | 0.49747 | 0.60235 | 0.0000 | 0.0001 | 0.0000 | 0.0000 | 0.0000 | 0.0000 |
| *Coriobacteriaceae UCG-002* | 0.49749 | 0.60235 | 0.0000 | 0.0000 | 0.0000 | 0.0000 | 0.0000 | 0.0000 |
| *Phaeodactylibacter* | 0.49781 | 0.60235 | 0.0000 | 0.0000 | 0.0000 | 0.0000 | 0.0001 | 0.0000 |
| *Thermoactinomyces* | 0.49819 | 0.60235 | 0.0000 | 0.0000 | 0.0000 | 0.0000 | 0.0000 | 0.0000 |
| *uncultured bacterium* | 0.50197 | 0.60235 | 0.0681 | 0.0599 | 0.0877 | 0.0590 | 0.1136 | 0.0901 |
| *Rheinheimera* | 0.50352 | 0.60235 | 0.0000 | 0.0000 | 0.0000 | 0.0000 | 0.0000 | 0.0000 |
| *Pseudofulvibacter* | 0.51073 | 0.60235 | 0.0000 | 0.0000 | 0.0000 | 0.0000 | 0.0000 | 0.0000 |
| *Lutibacter* | 0.51369 | 0.60235 | 0.0000 | 0.0000 | 0.0000 | 0.0000 | 0.0000 | 0.0000 |
| *Intestinimonas* | 0.51643 | 0.60235 | 0.0010 | 0.0010 | 0.0012 | 0.0008 | 0.0007 | 0.0022 |
| *Lachnospiraceae NK4B4 group* | 0.51930 | 0.60235 | 0.0000 | 0.0029 | 0.0001 | 0.0009 | 0.0001 | 0.0000 |
| *Corynebacterium 1* | 0.52083 | 0.60235 | 0.0000 | 0.0000 | 0.0000 | 0.0000 | 0.0000 | 0.0001 |
| *Desulfobulbus* | 0.52196 | 0.60235 | 0.0000 | 0.0000 | 0.0000 | 0.0000 | 0.0000 | 0.0000 |
| *Anaerovibrio* | 0.52344 | 0.60235 | 0.0000 | 0.0000 | 0.0000 | 0.0000 | 0.0000 | 0.0001 |
| *Sutterella* | 0.52438 | 0.60235 | 0.0000 | 0.0000 | 0.0000 | 0.0000 | 0.0000 | 0.0000 |
| *Actibacter* | 0.52489 | 0.60235 | 0.0000 | 0.0000 | 0.0000 | 0.0000 | 0.0000 | 0.0000 |
| *Photobacterium* | 0.52777 | 0.60235 | 0.0000 | 0.0000 | 0.0000 | 0.0000 | 0.0000 | 0.0000 |
| *Candidatus Soleaferrea* | 0.53162 | 0.60235 | 0.0000 | 0.0000 | 0.0000 | 0.0000 | 0.0000 | 0.0000 |
| *Actinomyces* | 0.53281 | 0.60235 | 0.0000 | 0.0000 | 0.0000 | 0.0000 | 0.0000 | 0.0000 |
| *Fluviicola* | 0.53699 | 0.60235 | 0.0000 | 0.0000 | 0.0000 | 0.0000 | 0.0000 | 0.0000 |
| *uncultured* | 0.54046 | 0.60235 | 0.0440 | 0.0238 | 0.0306 | 0.0368 | 0.0384 | 0.0207 |
| *Thiogranum* | 0.54334 | 0.60235 | 0.0000 | 0.0000 | 0.0000 | 0.0000 | 0.0000 | 0.0000 |
| *Macrococcus* | 0.54612 | 0.60235 | 0.0000 | 0.0000 | 0.0000 | 0.0001 | 0.0000 | 0.0000 |
| *Streptococcus* | 0.54639 | 0.60235 | 0.0087 | 0.0040 | 0.0043 | 0.0036 | 0.0028 | 0.0013 |
| *Atopostipes* | 0.55268 | 0.60235 | 0.0000 | 0.0000 | 0.0000 | 0.0000 | 0.0000 | 0.0000 |
| *Blautia* | 0.55516 | 0.60235 | 0.0119 | 0.0006 | 0.0082 | 0.0062 | 0.0042 | 0.0112 |
| *Massilia* | 0.55586 | 0.60235 | 0.0000 | 0.0000 | 0.0000 | 0.0000 | 0.0000 | 0.0000 |
| *Nocardioides* | 0.55700 | 0.60235 | 0.0000 | 0.0000 | 0.0000 | 0.0000 | 0.0000 | 0.0000 |
| *Pseudomonas* | 0.55720 | 0.60235 | 0.0000 | 0.0000 | 0.0000 | 0.0000 | 0.0000 | 0.0000 |
| *Draconibacterium* | 0.55798 | 0.60235 | 0.0000 | 0.0000 | 0.0000 | 0.0000 | 0.0000 | 0.0000 |
| *Marinicella* | 0.55798 | 0.60235 | 0.0000 | 0.0000 | 0.0000 | 0.0000 | 0.0000 | 0.0000 |
| *Spirochaeta 2* | 0.55798 | 0.60235 | 0.0000 | 0.0000 | 0.0000 | 0.0000 | 0.0000 | 0.0000 |
| *Desulfuromusa* | 0.55895 | 0.60235 | 0.0000 | 0.0000 | 0.0000 | 0.0000 | 0.0000 | 0.0000 |
| *Idiomarina* | 0.55895 | 0.60235 | 0.0000 | 0.0000 | 0.0000 | 0.0000 | 0.0000 | 0.0000 |
| *Peredibacter* | 0.55895 | 0.60235 | 0.0000 | 0.0000 | 0.0000 | 0.0000 | 0.0000 | 0.0000 |
| *Thalassolituus* | 0.55895 | 0.60235 | 0.0000 | 0.0000 | 0.0000 | 0.0000 | 0.0000 | 0.0000 |
| *Lachnospiraceae AC2044 group* | 0.55932 | 0.60235 | 0.0000 | 0.0000 | 0.0001 | 0.0000 | 0.0000 | 0.0000 |
| *Mycoplasma* | 0.55969 | 0.60235 | 0.0000 | 0.0000 | 0.0000 | 0.0000 | 0.0000 | 0.0000 |
| *Gemmobacter* | 0.55985 | 0.60235 | 0.0000 | 0.0000 | 0.0000 | 0.0000 | 0.0000 | 0.0000 |
| *Roseivivax* | 0.56001 | 0.60235 | 0.0000 | 0.0000 | 0.0000 | 0.0000 | 0.0000 | 0.0000 |
| *Sporosarcina* | 0.56022 | 0.60235 | 0.0000 | 0.0000 | 0.0000 | 0.0000 | 0.0000 | 0.0000 |
| *Ruminococcaceae UCG-003* | 0.56058 | 0.60235 | 0.0000 | 0.0000 | 0.0000 | 0.0000 | 0.0000 | 0.0000 |
| *Parasutterella* | 0.57155 | 0.61174 | 0.0001 | 0.0000 | 0.0005 | 0.0000 | 0.0001 | 0.0003 |
| *Oscillospira* | 0.59738 | 0.63689 | 0.0000 | 0.0000 | 0.0000 | 0.0000 | 0.0000 | 0.0000 |
| *Tyzzerella* | 0.61407 | 0.65215 | 0.0018 | 0.0009 | 0.0014 | 0.0016 | 0.0020 | 0.0021 |
| *Prevotella 7* | 0.63234 | 0.66896 | 0.0001 | 0.0000 | 0.0002 | 0.0000 | 0.0000 | 0.0001 |
| *Sulfurovum* | 0.64694 | 0.68177 | 0.0000 | 0.0000 | 0.0000 | 0.0000 | 0.0000 | 0.0000 |
| *Maritimimonas* | 0.66246 | 0.69546 | 0.0000 | 0.0000 | 0.0000 | 0.0000 | 0.0000 | 0.0000 |
| *unidentified* | 0.68099 | 0.71218 | 0.0002 | 0.0001 | 0.0002 | 0.0000 | 0.0000 | 0.0000 |
| *Paracocccus* | 0.69513 | 0.72420 | 0.0000 | 0.0000 | 0.0000 | 0.0000 | 0.0000 | 0.0000 |
| *Subdoligranulum* | 0.70354 | 0.73019 | 0.0000 | 0.0000 | 0.0000 | 0.0000 | 0.0000 | 0.0000 |
| *Micrococcus* | 0.72821 | 0.75294 | 0.0000 | 0.0000 | 0.0000 | 0.0000 | 0.0000 | 0.0000 |
| *Ruminiclostridium 6* | 0.73198 | 0.75400 | 0.0034 | 0.0010 | 0.0010 | 0.0011 | 0.0012 | 0.0039 |
| *Prevotellaceae NK3B31 group* | 0.75013 | 0.76979 | 0.0001 | 0.0001 | 0.0002 | 0.0001 | 0.0001 | 0.0003 |
| *Anaerofustis* | 0.78307 | 0.80060 | 0.0000 | 0.0000 | 0.0000 | 0.0000 | 0.0000 | 0.0000 |
| *Marvinbryantia* | 0.78763 | 0.80227 | 0.0036 | 0.0030 | 0.0020 | 0.0048 | 0.0024 | 0.0022 |
| *Lachnospiraceae Incertae Sedis* | 0.79057 | 0.80228 | 0.0030 | 0.0016 | 0.0010 | 0.0014 | 0.0022 | 0.0012 |
| *Lachnospiraceae UCG-001* | 0.82583 | 0.83498 | 0.0188 | 0.0103 | 0.0239 | 0.0175 | 0.0141 | 0.0204 |
| *Oscillibacter* | 0.89792 | 0.90452 | 0.0041 | 0.0024 | 0.0022 | 0.0030 | 0.0030 | 0.0041 |
| *Tyzzerella 3* | 0.90278 | 0.90609 | 0.0002 | 0.0003 | 0.0002 | 0.0002 | 0.0001 | 0.0002 |
| *Faecalibacterium* | 0.92082 | 0.92082 | 0.0001 | 0.0000 | 0.0000 | 0.0000 | 0.0000 | 0.0001 |
