# Supplementary material for: Alterations of Gut Microbiome and Metabolite Profiling in Mice Infected by Schistosoma japonicum
Source: Front Immunol. 2020 Oct 8;11:569727. doi: 10.3389/fimmu.2020.569727 (PMC7580221; doi:10.3389/fimmu.2020.569727)
Supplement: Supplementary Table 6 — Statistically significantly changed metabolites identified in serum samples, urine samples, liver aqueous extracts and colon aqueous extracts (p < 0.05). [file Table_6.DOCX]

| Source | Mode | No. | m/z | Retention time (min) | Adducts | Formula | Metabolites | VIP value | | | | | |
| --- | --- | --- | --- | --- | --- | --- | --- | --- | --- | --- | --- | --- | --- |
|  |  |  |  |  |  |  |  | 3 dpi vs. 0 dpi | 7 dpi vs. 0 dpi | 14 dpi vs. 0 dpi | 21 dpi vs. 0 dpi | 28 dpi vs. 0 dpi | 42 dpi vs. 0 dpi |
| Serum | ESI+ | 1 | 204.1244 | 0.70 | M+H | C9H17NO4 | Acetylcarnitine |  | 3.854 | 4.178 | 4.694 | 3.443 | 4.081 |
|  |  | 2 | 137.0468 | 1.10 | M+H | C5H4N4O | Hypoxanthine |  | 2.396 |  |  |  |  |
|  |  | 3 | 291.0706 | 1.75 | M+Na, M+K | C10H12N4O5 | Arabinosylhypoxanthine |  | 2.190 |  |  |  |  |
|  |  | 4 | 526.3135 | 7.31 | M+H | C24H48NO9P | PS(18:0/0:0) |  |  | 2.617 |  | 2.285 |  |
|  |  | 5 | 426.3573 | 7.64 | M+H | C25H47NO4 | Oleoylcarnitine |  | 2.397 | 2.038 | 2.650 |  |  |
|  |  | 6 | 756.5518 | 12.51 | M+H | C42H78NO8P | PC(18:3(9Z,12Z,15Z)/16:0) | 4.008 | 4.066 | 3.042 | 2.854 | 2.767 |  |
|  |  | 7 | 729.5881 | 12.72 | M+H, M+Na | C41H81N2O6P | SM(d18:1/18:1(9Z)) | 2.592 | 2.000 |  |  |  | 3.090 |
|  |  | 8 | 705.5872 | 12.89 | M+H, M+Na | C39H81N2O6P | SM(d18:0/16:0) |  |  |  |  |  | 2.787 |
|  |  | 9 | 830.5675 | 13.02 | M+Na | C46H82NO8P | PC(22:5(7Z,10Z,13Z,16Z,19Z)/16:0) | 3.002 | 4.079 | 3.226 | 3.197 | 3.264 |  |
|  |  | 10 | 820.5790 | 13.13 | M+H | C47H82NO8P | PC(22:6(4Z,7Z,10Z,13Z,16Z,19Z)/17:0) | 4.472 | 5.373 | 4.198 | 3.875 | 3.841 | 3.013 |
|  |  | 11 | 563.4048 | 13.15 | M+K | C32H60O5 | DG(14:1(9Z)/15:0/0:0) |  |  |  | 2.357 | 2.391 |  |
|  |  | 12 | 595.4309 | 13.43 | M+Na | C36H60O5 | DG(13:0/20:5(5Z,8Z,11Z,14Z,17Z)/0:0)[iso2] |  |  |  |  | 2.302 |  |
|  |  | 13 | 734.5677 | 13.52 | M+H, M+Na, 2M+H | C40H80NO8P | Colfosceril palmitate | 9.524 | 5.022 | 12.406 | 2.291 | 8.588 |  |
|  |  | 14 | 731.6035 | 13.55 | M+H, M+Na | C41H83N2O6P | SM(d18:0/18:1(11Z)) | 5.846 | 4.783 | 2.559 | 3.237 | 3.690 | 6.829 |
|  |  | 15 | 744.5872 | 13.58 | M+H | C42H82NO7P | PC(P-18:1(9Z)/16:0) |  |  |  |  | 2.602 | 4.798 |
|  |  | 16 | 834.7153 | 13.60 | M+CH3OH+H | C46H94N2O6P+ | SM(d17:1/24:0) | 2.387 | 2.163 |  |  |  |  |
|  |  | 17 | 770.5983 | 13.68 | M+H | C44H84NO7P | PC(o-18:1(9Z)/18:2(9Z,12Z)) |  |  |  |  |  | 3.955 |
|  |  | 18 | 623.4612 | 13.97 | M+Na | C38H64O5 | DG(15:0/20:5(5Z,8Z,11Z,14Z,17Z)/0:0) |  |  |  | 2.132 | 2.117 |  |
|  |  | 19 | 603.4382 | 14.00 | M+K | C35H64O5 | DG(14:1(9Z)/18:1(11Z)/0:0) |  |  | 2.191 | 2.375 | 2.473 |  |
|  |  | 20 | 818.6024 | 14.05 | M+H | C48H84NO7P | PC(P-18:1(9Z)/22:5(4Z,7Z,10Z,13Z,16Z)) | 2.021 |  |  | 3.480 | 2.554 | 2.567 |
|  |  | 21 | 820.6174 | 14.20 | M+H, M+Na | C48H86NO7P | PC(P-18:0/22:5(4Z,7Z,10Z,13Z,16Z)) |  | 4.413 | 5.467 | 6.211 | 5.583 | 5.675 |
|  |  | 22 | 746.6024 | 14.26 | M+H | C42H84NO7P | PC(18:0/P-16:0) |  |  |  |  |  | 4.985 |
|  |  | 23 | 715.5253 | 14.37 | M+H, M+Na | C45H72O5 | DG(20:3n6/0:0/22:5n6) |  |  | 2.611 | 2.363 | 2.995 |  |
|  |  | 24 | 762.5981 | 14.47 | M+H | C42H84NO8P | PC(18:0/16:0) | 4.887 |  | 7.923 | 2.808 | 5.565 |  |
|  |  | 25 | 752.5593 | 14.59 | M+H, M+Na | C43H78NO7P | PE(P-18:0/20:4(8Z,11Z,14Z,17Z)) |  | 2.120 | 3.501 | 3.948 | 3.677 |  |
|  |  | 26 | 689.5091 | 14.76 | M+H, M+Na | C43H70O5 | DG(20:4(8Z,11Z,14Z,17Z)/20:3(5Z,8Z,11Z)/0:0) |  |  |  |  |  | 2.126 |
|  |  | 27 | 717.5395 | 14.84 | M+H | C40H77O8P | PA(22:1(13Z)/15:0) |  |  |  | 2.011 | 2.263 |  |
|  |  | 28 | 846.6347 | 14.94 | M+H-H2O | C50H90NO8P | PC(20:3(8Z,11Z,14Z)/22:2(13Z,16Z)) |  |  |  | 2.730 | 2.087 | 2.073 |
|  |  | 29 | 799.6638 | 15.19 | M+H | C46H91N2O6P | SM(d18:2/23:0) |  |  |  |  |  | 2.498 |
|  |  | 30 | 716.6149 | 15.34 | M+H-H2O, M+H | C40H80N2O6P+ | SM C16:1 |  |  | 2.476 | 2.691 | 2.878 |  |
|  |  | 31 | 790.6340 | 15.36 | M+H | C44H88NO8P | PC(16:0/20:0) | 2.255 |  | 2.491 |  |  |  |
|  |  | 32 | 776.5930 | 15.42 | M+H-H2O | C46H84NO7P | PC(20:4(8Z,11Z,14Z,17Z)/P-18:0) |  |  | 2.675 | 3.033 | 2.651 |  |
|  |  | 33 | 670.6097 | 16.56 | M+H-H2O, M+H, M+Na | C42H81NO3 | Ceramide (d18:1/24:1(15Z)) |  |  |  |  |  | 3.110 |
|  | ESI- | 34 | 151.0258 | 1.29 | M-H | C5H4N4O2 | Xanthine |  | 2.500 | 2.230 |  | 2.261 | 2.338 |
|  |  | 35 | 619.2883 | 7.36 | M-H | C29H49O12P | (2R)-2-Hydroxy-3-[(hydroxy{[(2R,3R,5S,6R)-2,3,4,5,6- pentahydroxycyclohexyl]oxy}phosphoryl)oxy] propyl (5Z,11Z)-5,8,11,14-icosatetraenoate |  |  |  |  |  | 3.547 |
|  |  | 36 | 588.3304 | 7.77 | M+FA-H | C28H50NO7P | LysoPC(20:4(5Z,8Z,11Z,14Z)) |  |  | 2.652 | 2.347 | 2.267 | 3.547 |
|  |  | 37 | 321.2431 | 7.99 | M-H, M+NaFA-H | C20H34O3 | 8-HETrE |  |  |  |  |  | 2.804 |
|  |  | 38 | 850.5603 | 14.38 | M+FA-H | C46H80NO8P | PC(22:6(4Z,7Z,10Z,13Z,16Z,19Z)/16:0) | 9.264 | 10.378 | 10.696 | 10.112 | 10.421 | 7.740 |
|  |  | 39 | 909.5470 | 14.59 | M-H | C49H83O13P | PI(22:6(4Z,7Z,10Z,13Z,16Z,19Z)/18:0) |  |  |  |  |  | 2.146 |
|  |  | 40 | 795.5403 | 14.83 | M-H | C41H81O12P | PI(O-16:0/16:0) |  |  | 3.181 | 4.969 | 5.620 |  |
|  |  | 41 | 878.5912 | 15.51 | M+FA-H | C48H84NO8P | PC(22:6(4Z,7Z,10Z,13Z,16Z,19Z)/18:0) | 8.519 | 7.675 | 7.595 | 8.846 | 6.197 | 2.018 |
|  |  | 42 | 854.5913 | 15.77 | M+FA-H | C46H84NO8P | PC(22:4(7Z,10Z,13Z,16Z)/16:0) | 10.281 | 10.700 | 10.058 | 12.253 | 10.022 | 9.377 |
| Urine | ESI+ | 1 | 388.2531 | 4.53 | M+H | C20H37NO4S | N-linoleoyl taurine |  |  |  | 3.769 | 4.641 | 6.776 |
|  |  | 2 | 371.2269 | 4.53 | M+H, M+Na | C16H34O9 | Octaethylene glycol |  |  |  | 4.633 | 5.640 | 8.576 |
|  |  | 3 | 327.2009 | 4.20 | M+H, M+Na | C14H30O8 | Heptaethylene glycol |  |  |  | 4.553 | 5.475 | 8.561 |
|  |  | 4 | 283.1738 | 3.83 | M+H | C12H26O7 | Hexaethylene glycol |  |  |  |  | 2.141 | 3.314 |
|  |  | 5 | 206.0453 | 3.22 | M+H-H2O, M+H | C10H7NO4 | Xanthurenic acid | 8.159 | 12.518 | 10.979 | 8.124 | 9.387 | 5.641 |
|  |  | 6 | 189.0872 | 2.88 | M+H | C7H12N2O4 | N-Acetylglutamine |  |  |  | 2.464 | 3.900 | 2.880 |
|  |  | 7 | 341.1775 | 4.28 | M+H | C21H24O4 | 2'-O-Methylphaseollidinisoflavan |  |  |  |  |  | 2.616 |
|  |  | 8 | 229.1550 | 1.37 | M+H | C11H20N2O3 | Leucylproline |  | 3.602 |  | 3.162 | 5.494 | 7.006 |
|  |  | 9 | 302.1478 | 4.86 | M+H | C16H19N3O3 | Tryptophyl-Proline |  |  |  |  |  | 2.489 |
|  |  | 10 | 262.0383 | 1.06 | M+H, 2M+H | C9H11NO6S | 2-methoxyacetaminophen sulfate |  | 2.103 | 2.233 |  |  |  |
|  |  | 11 | 331.2246 | 7.89 | M+H-H2O, M+H | C12H26N8O3 | Arginyl-Arginine |  |  |  |  |  | 2.208 |
|  |  | 12 | 149.0964 | 4.97 | M+H-H2O, M+H | C10H14O2 | Perillic acid | 3.477 | 2.613 | 3.328 |  |  |  |
|  |  | 13 | 263.1058 | 4.23 | M+H, M+CH3OH+H | C10H18N2O4S | Hydroxyprolyl-Methionine | 2.604 |  |  |  |  |  |
|  |  | 14 | 366.1931 | 7.85 | M+H-H2O, M+H | C19H27NO6 | senkirkine |  |  |  |  | 2.262 |  |
|  |  | 15 | 262.1649 | 1.56 | M+H | C12H23NO5 | hydroxyisovaleroyl carnitine | 3.812 |  |  |  |  |  |
|  |  | 16 | 228.0628 | 6.11 | M+Na | C11H11NO3 | Cinnamoylglycine |  |  | 2.683 | 2.547 | 3.193 | 2.757 |
|  |  | 17 | 181.0614 | 3.38 | M+H-H2O, M+H, M+K | C8H8N2O3 | Nicotinuric acid |  | 3.948 | 4.236 | 3.753 | 2.972 | 3.880 |
|  |  | 18 | 162.0543 | 3.59 | M+H-H2O, M+H | C9H7NO2 | 1H-Indole-2-carboxylic acid | 2.038 | 5.120 | 4.997 | 3.876 |  | 2.071 |
|  |  | 19 | 190.0505 | 3.59 | M+H-H2O, M+H | C10H7NO3 | 1-ACETYLISATIN | 4.494 | 7.723 | 7.389 | 5.156 |  | 2.982 |
|  |  | 20 | 304.1753 | 3.43 | M+H | C14H25NO6 | Pimelylcarnitine | 5.098 |  |  | 3.876 | 2.602 | 2.284 |
|  |  | 21 | 228.0508 | 2.54 | M+H | C9H9NO6 | Stizolobic acid | 2.256 |  |  |  |  |  |
|  |  | 22 | 131.0497 | 6.11 | M+H-H2O, M+K | C9H8O2 | Cinnamic acid |  | 2.092 | 5.564 | 6.820 | 7.688 | 7.025 |
|  |  | 23 | 180.0885 | 2.81 | M+H | C7H9N5O | 7-Aminomethyl-7-carbaguanine |  | 2.988 | 3.418 |  |  |  |
|  |  | 24 | 719.4871 | 7.91 | M+H | C38H71O10P | PG(16:1(9Z)/16:1(9Z)) |  |  |  | 2.361 |  | 2.099 |
|  |  | 25 | 245.1492 | 1.75 | M+H | C11H20N2O4 | Leucyl-Hydroxyproline |  |  |  | 2.236 | 2.542 | 2.137 |
|  |  | 26 | 531.2984 | 5.53 | M+H-H2O, M+H | C28H42N4O6 | Kukoamine A |  | 2.614 | 2.426 |  |  |  |
|  |  | 27 | 706.5086 | 7.91 | M+H | C37H72NO9P | PS(O-16:0/15:1(9Z)) |  |  |  | 2.346 | 2.220 | 2.144 |
|  |  | 28 | 517.2633 | 5.36 | M+H, M+Na | C25H40O11 | Eriojaposide B |  | 2.140 | 2.402 |  |  |  |
|  |  | 29 | 336.1797 | 7.96 | M+H | C18H25NO5 | Senecionine | 2.293 |  |  |  |  |  |
|  | ESI- | 30 | 209.0941 | 6.88 | M-H | C10H14N2O3 | Aprobarbital |  |  |  | 2.473 | 2.389 | 4.224 |
|  |  | 31 | 365.2324 | 7.92 | M-H | C21H34O5 | Cortolone |  |  |  |  | 2.670 | 4.125 |
|  |  | 32 | 365.1348 | 1.98 | M+FA-H | C16H20N2O5 | Formylfusarochromanone |  | 2.186 |  |  |  | 2.272 |
|  |  | 33 | 225.0874 | 2.55 | M+FA-H | C9H12N2O2 | 3-Hydroxykynurenamine |  |  |  |  |  | 2.789 |
|  |  | 34 | 181.9914 | 3.01 | M-H | C7H5NO3S | Saccharin | 2.034 | 2.836 |  |  |  | 2.685 |
|  |  | 35 | 275.0225 | 3.88 | M-H | C10H12O7S | Dihydroferulic acid 4-sulfate |  |  |  |  | 2.668 | 3.213 |
|  |  | 36 | 160.0400 | 3.23 | M-H | C9H7NO2 | 2-Indolecarboxylic acid |  | 3.705 | 3.245 |  |  |  |
|  |  | 37 | 324.0910 | 0.61 | M-H | C11H19NO10 | NeuNGc |  |  |  |  |  | 2.347 |
|  |  | 38 | 261.0067 | 2.72 | M-H | C9H10O7S | Dihydrocaffeic acid 3-sulfate | 2.415 |  |  |  |  |  |
|  |  | 39 | 449.1083 | 6.89 | M-H | C21H22O11 | Phloretin 2'-O-glucuronide | 2.239 |  |  |  |  |  |
|  |  | 40 | 349.0917 | 4.99 | M+FA-H | C11H16N2O8 | N-Acetylaspartylglutamic acid | 2.391 |  |  | 2.121 |  |  |
|  |  | 41 | 331.1027 | 2.12 | M-H, M+FA-H | C14H20O9 | Leonuriside A | 3.588 |  |  |  |  | 2.454 |
|  |  | 42 | 363.2171 | 8.99 | M+FA-H | C20H30O3 | Oxymesterone |  |  |  |  |  | 2.833 |
|  |  | 43 | 253.0177 | 5.11 | M+FA-H | C10H8O3S | naphthalenesulfonic acid | 5.963 |  |  | 2.249 |  |  |
|  |  | 44 | 431.0981 | 5.09 | M-H | C21H20O10 | Dihydrodaidzein 7-O-glucuronide | 3.015 |  |  | 2.376 |  |  |
|  |  | 45 | 257.0813 | 7.28 | M-H | C15H14O4 | 3-phenyl-3,4-dihydro-2H-1-benzopyran-4,6,7-triol | 2.922 |  | 2.753 |  |  |  |
|  |  | 46 | 341.0859 | 4.43 | M-H | C15H18O9 | 3,4,5-trihydroxy-6-{[3-(4-hydroxyphenyl)propanoyl]oxy} oxane-2-carboxylic acid | 2.051 |  |  |  |  |  |
|  |  | 47 | 429.0491 | 3.77 | M+FA-H | C16H16O9S | {3-[3-(3,4-dihydroxyphenyl)-3-oxopropyl]-6-hydroxy- 2-methoxyphenyl}oxidanesulfonic acid |  |  |  | 2.894 | 2.561 |  |
|  |  | 48 | 529.0653 | 6.45 | M-H | C16H24N2O14P2 | dTDP-4-dehydro-2,6-dideoxy-D-glucose | 3.319 | 2.038 |  |  |  |  |
|  |  | 49 | 715.1720 | 2.76 | M+FA-H | C29H34O18 | 6-[(2-{3-[(6-carboxy-3,4,5-trihydroxyoxan-2-yl)oxy]-4-(methoxymethyl) phenyl}-3,5-dihydroxy-3,4-dihydro-2H-1-benzopyran-7-yl)oxy]- 3,4,5-trihydroxyoxane-2-carboxylic acid | 3.181 |  |  | 2.117 | 2.136 |  |
|  |  | 50 | 160.0764 | 6.13 | M-H | C10H11NO | Tryptophanol |  |  | 2.011 | 3.527 | 2.989 | 3.285 |
|  |  | 51 | 371.1342 | 8.57 | M+FA-H, M+NaFA-H | C16H22O7 | Hinokitiol glucoside | 3.828 |  | 3.488 |  |  | 3.193 |
|  |  | 52 | 380.0616 | 2.62 | M+FA-H | C11H16N2O8P+ | beta-nicotinamide D-ribonucleotide |  | 2.570 |  | 3.276 | 2.334 | 2.472 |
|  |  | 53 | 385.1853 | 6.51 | M-H | C19H30O8 | Corchoionol C 9-glucoside | 2.226 |  |  |  |  |  |
| Liver | ESI- | 1 | 167.0204 | 0.84 | M-H | C5H4N4O3 | Uric acid |  |  |  |  | 2.294 | 2.603 |
|  |  | 2 | 180.0655 | 1.10 | M-H | C9H11NO3 | L-(-)-Tyrosine |  | 2.021 |  |  |  | 2.418 |
|  |  | 3 | 665.2119 | 0.64 | M-H, M+FA-H | C24H42O21 | (1S,2S,3R,4R,5R,6S)-2,3,4,5,6-Pentahydroxycyclohexyl alpha-D-galactopyranosyl-(1->6)-alpha-D-galactopyranosyl- (1->6)-alpha-D-galactopyranoside | 4.556 | 4.643 | 4.222 | 2.638 | 4.237 | 4.296 |
|  |  | 4 | 164.0709 | 2.52 | M-H | C9H11NO2 | L-(-)-Phenylalanine |  |  |  |  |  | 2.028 |
|  |  | 5 | 549.1655 | 0.64 | M-H, M+FA-H | C26H30O13 | Neolicuroside | 3.787 | 3.011 | 2.444 | 2.255 | 3.232 | 3.219 |
|  |  | 6 | 827.2632 | 0.65 | M-H, M+FA-H | C30H52O26 | alpha-maltopentaose | 2.761 | 3.232 | 2.999 |  | 2.815 | 2.758 |
|  |  | 7 | 539.1377 | 0.62 | 2M-H | C12H14O7 | Phenylglucuronide | 10.418 | 8.476 | 6.759 | 6.582 | 8.897 | 9.325 |
|  |  | 8 | 701.1901 | 0.63 | M+FA-H | C29H36O17 | Hellicoside | 6.172 | 6.567 | 6.113 | 3.983 | 5.918 | 6.055 |
|  |  | 9 | 615.1510 | 0.63 | M+FA-H | C32H26O10 | 6,6'-Dihydroxy-5,5',8,8'-tetramethoxy-2,2'-dimethyl- 4H,4'H-7,10'-bibenzo[g]chromene-4,4'-dione | 3.678 | 2.832 |  |  | 3.051 | 3.306 |
|  |  | 10 | 541.1353 | 0.62 | M-H | C27H26O12 | Resveratrol 4'-(2-galloylglucoside) | 6.236 | 5.061 | 4.042 | 3.933 | 5.347 | 5.621 |
|  |  | 11 | 384.0975 | 1.27 | M-H, 2M-H | C14H19N5O6S | S-inosyl-L-homocysteine | 2.262 | 3.711 |  |  | 2.079 |  |
|  |  | 12 | 503.1605 | 0.64 | M-H, 2M-H | C18H32O16 | (1S,2S,3R,4R,5R,6S)-2,3,4,5,6-Pentahydroxycyclohexyl 6-O-alpha-D-galactopyranosyl-alpha-D-galactopyranoside | 3.890 | 3.389 | 2.823 | 2.449 | 3.347 | 3.399 |
|  |  | 13 | 217.0296 | 0.61 | M-H, M+FA-H | C3H9O6P | Glycerol 3-phosphate | 2.495 | 2.530 |  |  | 2.240 |  |
|  |  | 14 | 1197.3617 | 0.79 | M-H, M+FA-H | C42H72O36 | Maltoheptaose |  | 2.874 | 2.249 |  | 2.378 | 2.325 |
|  |  | 15 | 243.0616 | 1.02 | M-H, M+FA-H, 2M-H | C9H12N2O6 | pseudouridine | 3.518 | 2.942 | 2.776 | 3.116 | 4.270 | 3.519 |
|  |  | 16 | 259.0217 | 0.60 | M-H | C6H13O9P | D-Glucose-6-dihydrogen phosphate |  |  |  |  | 4.128 | 2.201 |
|  |  | 17 | 341.1079 | 0.64 | M-H, 2M-H | C12H22O11 | Melibiose |  | 2.053 |  | 2.024 | 2.522 |  |
|  |  | 18 | 357.0883 | 3.65 | M-H, 2M-H | C11H23N2O7PS | Phosphopantetheine | 2.847 | 6.669 | 2.036 | 2.012 |  |  |
| Colon | ESI- | 1 | 459.2714 | 7.15 | M-H, M+FA-H | C27H40O6 | SAE475O6E4 |  | 2.596 | 2.577 | 2.578 |  |  |
|  |  | 2 | 391.2857 | 7.14 | M-H, M+FA-H, 2M-H | C24H40O4 | Deoxycholic acid | 4.847 | 11.517 | 11.415 | 11.475 | 7.260 | 8.672 |
|  |  | 3 | 293.1794 | 11.01 | M-H | C14H30O4S | 6VOC18EIBH |  | 4.699 | 4.660 | 3.122 | 6.313 | 3.240 |
|  |  | 4 | 265.1485 | 7.81 | M-H | C12H26O4S | laurilsulfate |  | 8.521 | 8.411 | 5.826 | 8.350 | 5.739 |
|  |  | 5 | 594.3769 | 13.56 | M+FA-H | C28H56NO7P | LysoPC(20:1(11Z)) | 4.041 | 3.291 | 2.230 | 3.422 | 3.174 | 3.432 |
|  |  | 6 | 566.3464 | 10.68 | M+FA-H, 2M+FA-H | C26H52NO7P | LysoPC(18:1(9Z)) | 6.971 | 2.679 | 3.692 | 5.923 | 5.767 | 6.941 |
